# Supplementary material for: Structure of the human marker of self 5-transmembrane receptor CD47
Source: Nat Commun. 2021 Sep 1;12:5218. doi: 10.1038/s41467-021-25475-w (PMC8410850; doi:10.1038/s41467-021-25475-w)
Supplement: Supplementary file 1 — Supplementary Information [file 41467_2021_25475_MOESM1_ESM.pdf]

SUPPLEMENTARY INFORMATION

Structure of the human marker of self 5-transmembrane receptor CD47

Fenalti *et al.*

<u>Brief description of what is included in this file</u>:

Supplementary Fig. 1 |Crystallographic lattice of $CD47^{BRIL}$-B6H12 and the Fab epitope on CD47 ECD.

Supplementary Fig. 2 |Electron density maps of the $CD47^{BRIL}$-B6H12 crystal structure.

Supplementary Fig. 3 |Analysis of CD47 helix III surface buried area and interactions with other helices.

Supplementary Fig. 4 |CD47 evolutionary pattern within mammalian species.

Supplementary Fig. 5 |Evolutionary analysis of the 5-TM CD47 receptor.

Supplementary Fig. 6 |Viral CD47-like receptors.

Supplementary Fig. 7 |Differential kinetic HDX-MS studies using wild type $CD47^{BRIL}$ and mutants.

Supplementary Fig. 8 |Transition of CD47 ECD macrostates is associated to Y184 movement.

Supplementary Fig. 9 |CD47 ECD s1-s2 transition.

Supplementary Fig. 10 | Cryo-EM reconstruction of the full length $CD47^{BRIL}$-Fab CC-90002 complex.

Supplementary Fig. 11 |The interface between the full length $CD47^{BRIL}$ and Fab B6H12.

Supplementary Table 1. Diffraction data and refinement statistics.

Supplementary Table 2. HDX summary for WT $CD47^{BRIL}$ mutants.

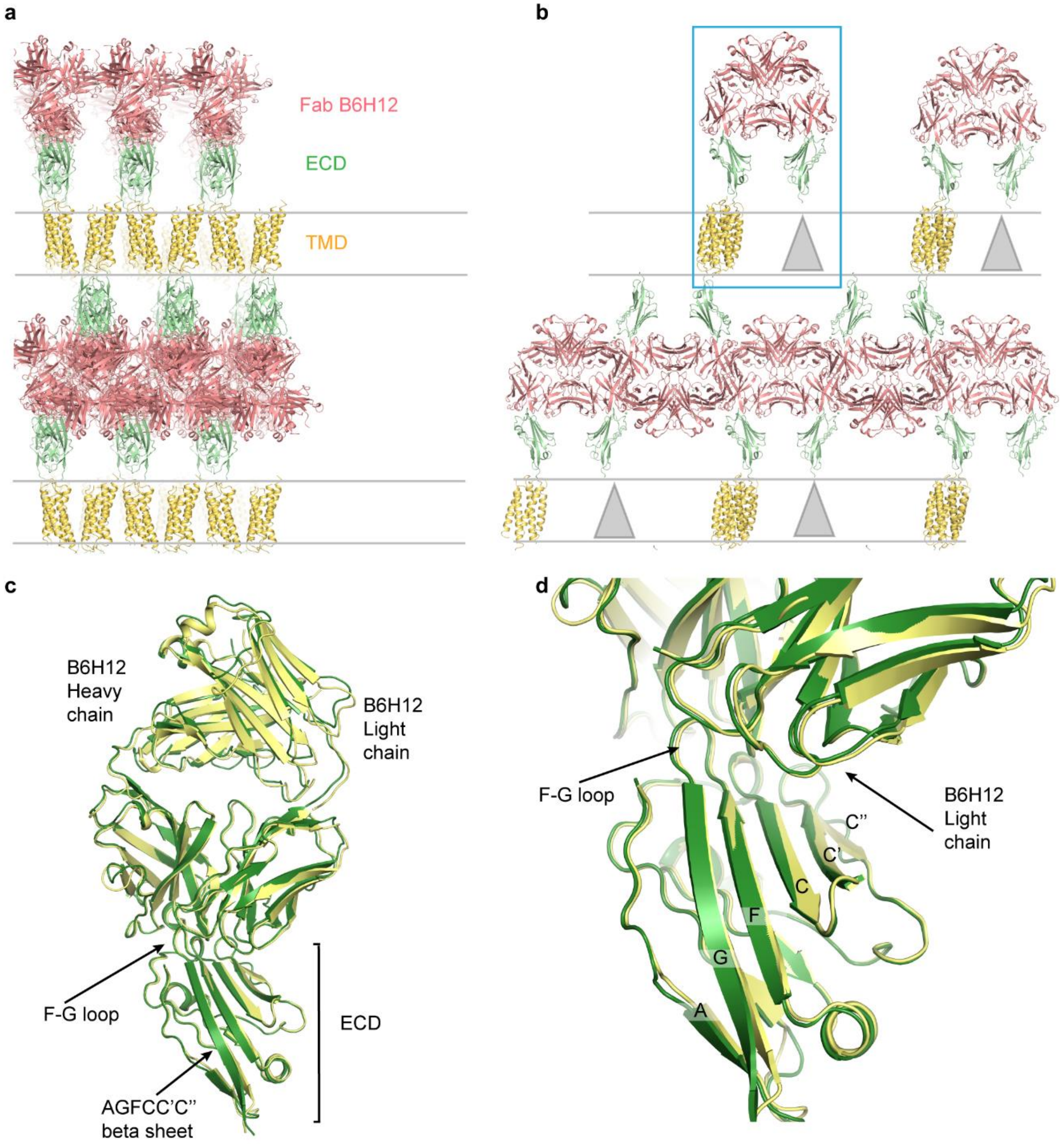

Supplementary Fig. 1 | Crystallographic lattice of CD47^{BRIL}-B6H12 and the Fab epitope on CD47 ECD. a-b Views of a section of the crystallographic lattice packing. Predicted membrane boundaries are indicated by grey lines. The CD47 ECD, TMD and the B6H12 Fab are colored green, yellow and pink respectively. CD47^{BRIL}-B6H12 complex dimer within the crystallographic asymmetric unit is indicated by a blue rectangle. The approximate position of the flexible CD47 TMD not modeled in the structure within the crystallographic dimer is indicated by a grey triangle. **c** The previously published crystal structure of the CD47 ECD in complex with Fab B6H12 (PDB ID 5TZU, yellow cartoon)[1] is superimposed on the equivalent CD47 atoms of the full length CD47^{BRIL}-B6H12 complex structure (green cartoon). **d** Close up view of the ECD-B6H12 interface highlighting the AGFCC'C'' β-sheet, which harbors the SIRPα binding site.

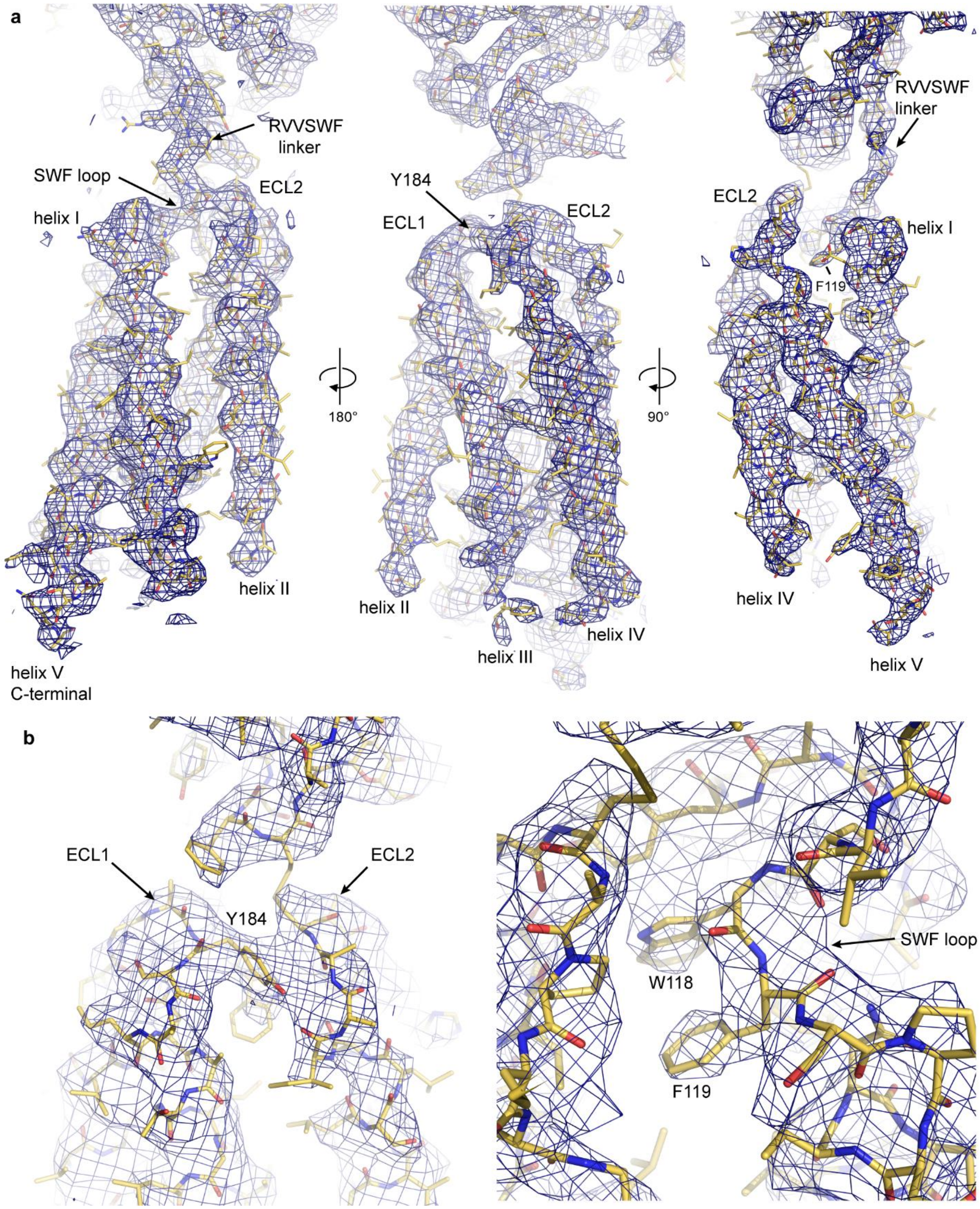

Supplementary Fig. 2 | Electron density maps of the CD47^{BRIL}-B6H12 crystal structure. a Views of an electron density map for helices in the CD47 TMD bundle and the inter-domain ^{114}RVVSWF^{119} linker. CD47 is shown as yellow sticks. Electron density of a feature-enhanced map (FEM)[2] calculated with PHENIX[3] is shown as a blue mesh and is contour at 1.0σ **b** Close up views of a feature-enhanced electron density maps in the Y184 and ^{117}SWF^{119} loop region contour at 1.0σ.

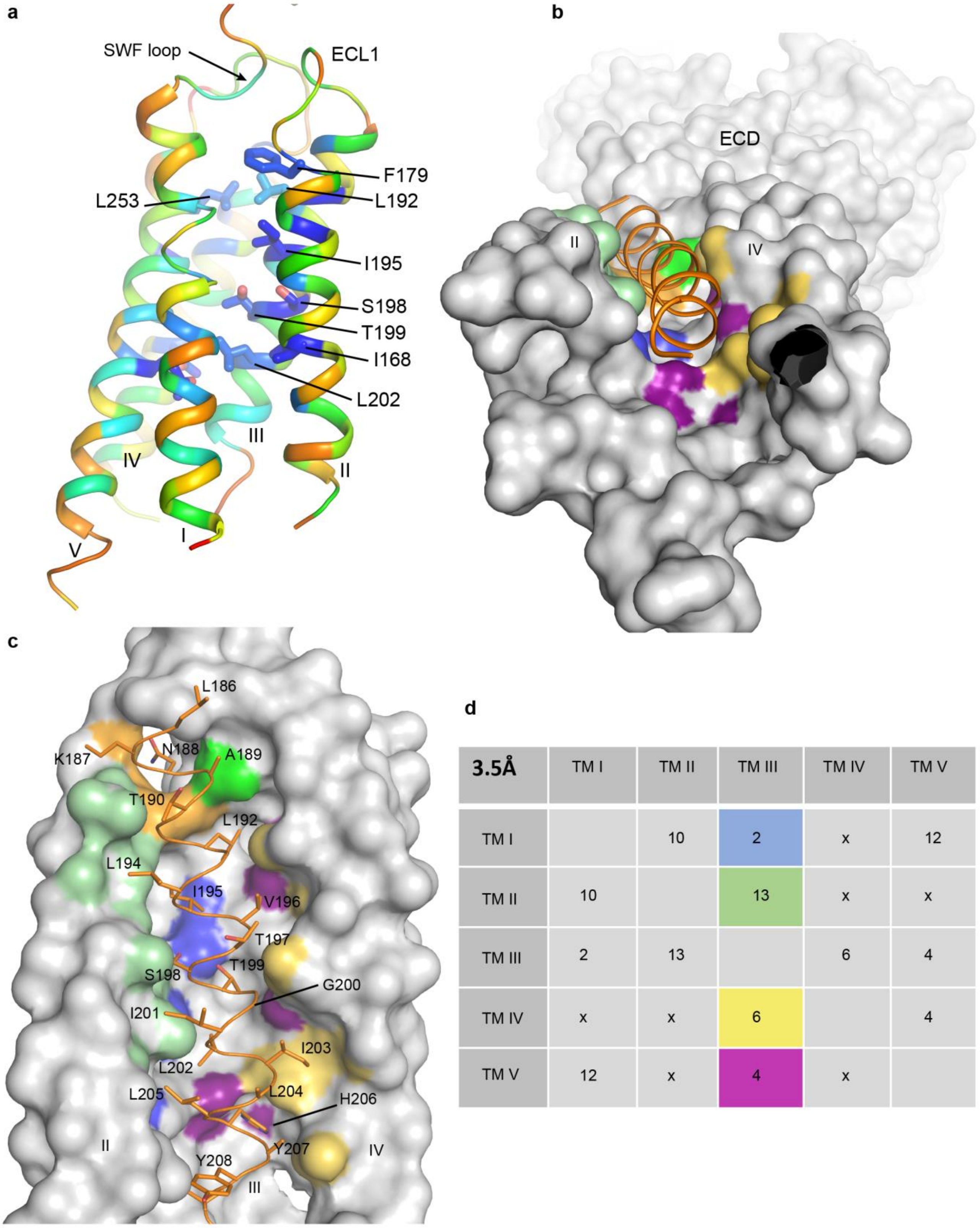

3.5Å	TM I	TM II	TM III	TM IV	TM V
TM I		10	2	x	12
TM II	10		13	x	x
TM III	2	13		6	4
TM IV	x	x	6		4
TM V	12	x	4	x	

Supplementary Fig. 3 | Analysis of CD47 helix III surface buried area and interactions with other helices. a Cartoon representation of CD47^{BRIL}-B6H12 TMD structure colored according to the surface buried area of individual residues using the software CHIMERA[4]. The color scheme follows a graded heat map (blue: buried; red: surface exposed). Labeled residues indicate residues with the largest buried surface within the CD47 TM bundle. **b** View from the intracellular face

toward the extracellular face of CD47^{BRIL}-B6H12 crystal structure. All CD47 atoms except for helix III (orange tube) are shown as a surface representation. Atoms from the TM helices I, II, IV and V, that are within 3.8Å or less from helix III atoms are highlighted by distinct surface colors (helix I: blue; helix II: pale green; helix IV, yellow: helix V purple; inter-domain ^{114}RVVSWF^{119} linker: green; ECL2 light orange); atoms further than 3.8Å are shown as a grey surface. **c** View of the interactions between helix III and the other helices of the TMD bundle shown with the same coloring scheme described in ‘b’. The side chains of helix III are shown and labeled. **d** Table showing the number of atoms from each helix that are within 3.5Å distance or less of atoms in other helices; table is color coded using the same color scheme for the helices as in ‘b’ (see Methods).

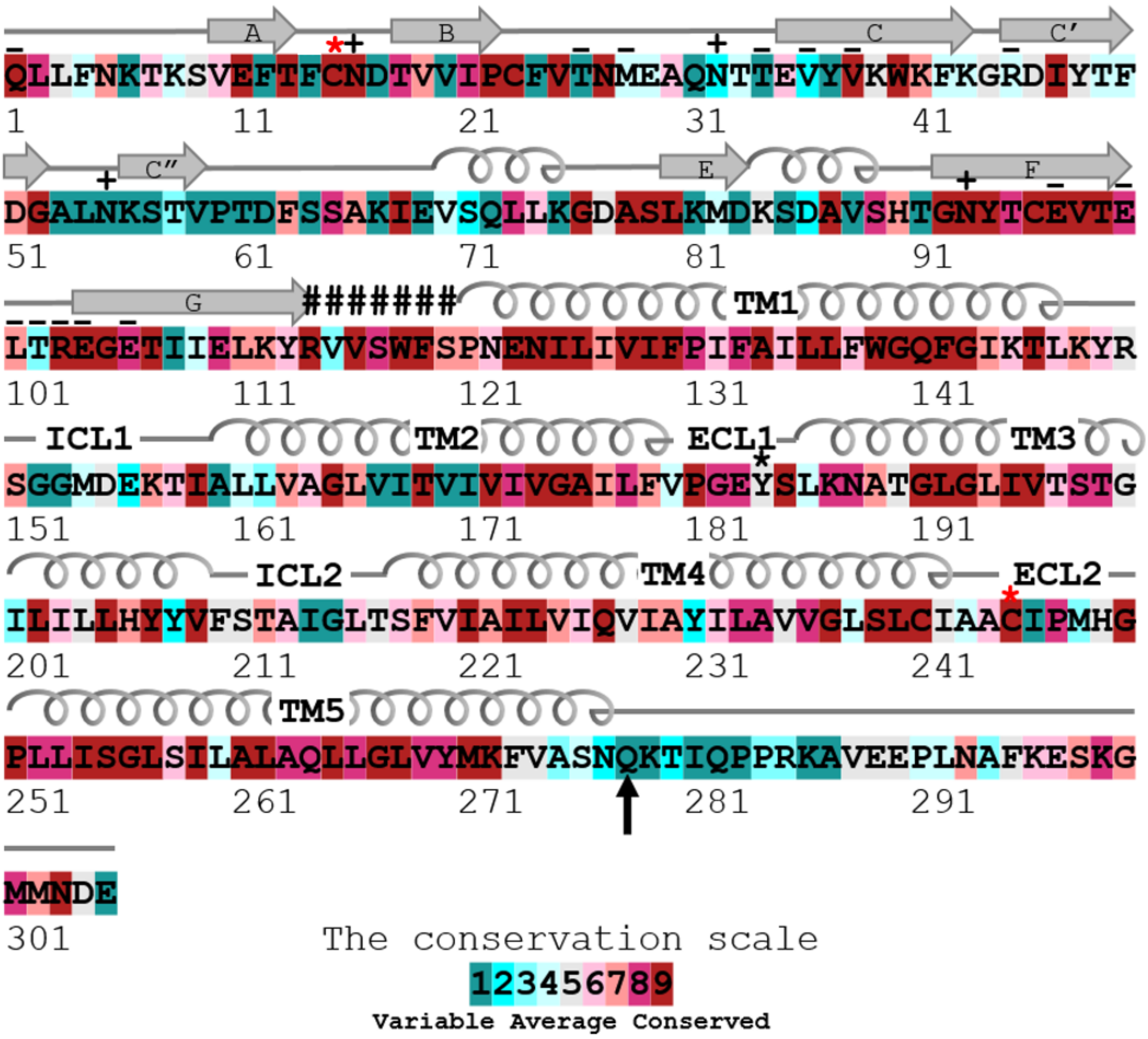

Supplementary Fig. 4 | CD47 evolutionary pattern within mammalian species. Amino acid conservation of CD47 across 72 mammal species mapped onto human CD47 sequence using the CONSURF algorithm[5]. The conservation scale (1-9) is color coded where the least conserved positions are shown with a dark teal background, average conservation with a grey background, and most conserved with a red background. CD47 secondary structure elements are shown above the sequence: straight lines indicate loops, arrows indicate beta strands and spiral lines indicate α-helices. The # symbols denotes the ^{114}RVVSWF^{119} peptide linker and an asterisk indicates the 'conformational switch' Y184. Red stars indicate cysteines involved in the inter-domain disulfide bond. N-linked glycosylated asparagine residues (modeled in the structure) are indicated by a + symbol. Amino acids that are within 3.5Å or less of SIRPα in the CD47 ECD-SIRPα crystal structure (PDB ID 2JJS)[6] are indicated by a line above the residue code. A vertical arrow indicates the last residue (Q278) modeled in the CD47^{BRIL}-B6H12 crystal structure. Amino acid sequence alignment data are provided in Supplementary Data 1. Source data are provided as a Source Data file.

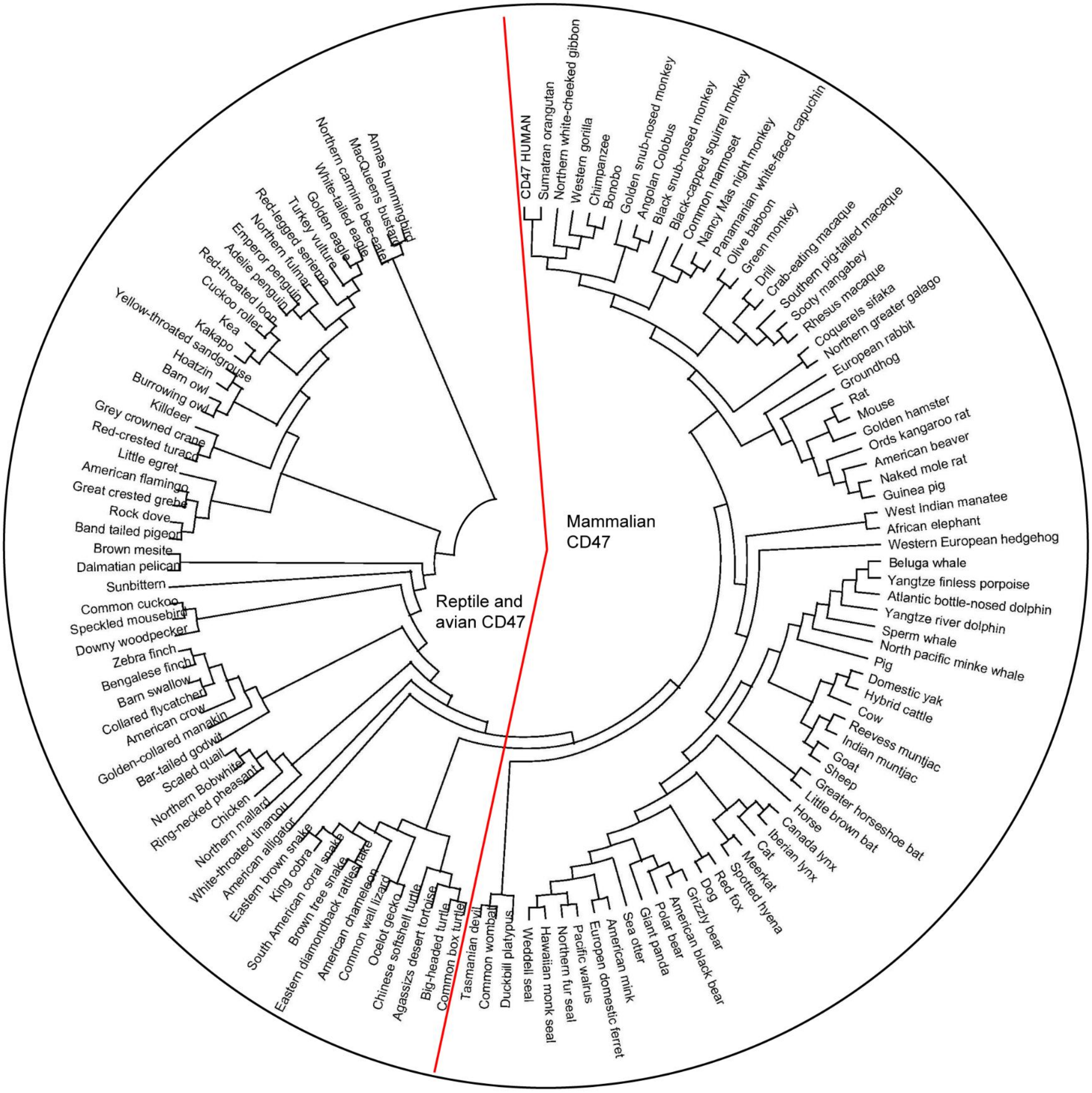

Supplementary Fig. 5 | Evolutionary analysis of the 5-TM CD47 receptor. Phylogenetic tree of CD47 from all vertebrate species. Red lines indicate the division between mammalian and reptile/avian CD47. Source data provided as a Source data file.

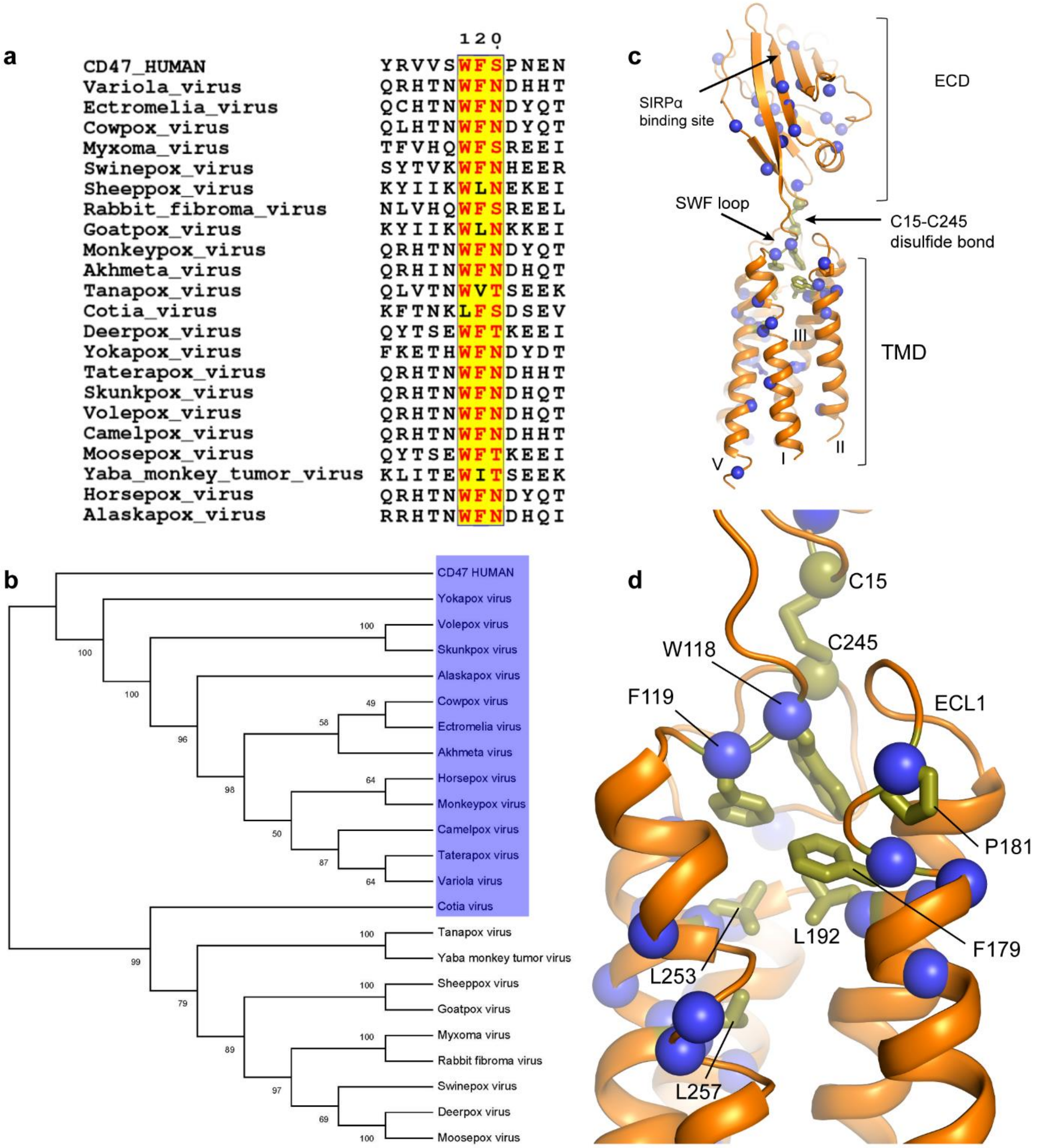

Supplementary Fig. 6 | Viral CD47-like receptors. a Section of a pairwise sequence alignment of viruses from the *Poxyviridea* family that express CD47-like receptors, and human CD47. Amino acid similarity around the ^{114}RVVSWF^{119} linker region is highlighted in yellow. **b** Phylogenetic tree of CD47-like receptors from viral orthologues and human CD47. Viral CD47-like receptors in the closest evolutionary branch to human CD47 are highlighted (blue box). **c** Mapping of conserved amino-acid residues between human CD47 and viral CD47-like receptors from the clade most closely related to human onto the CD47^{BRIL}-B6H12 structure (orange cartoon, B6H12 Fab omitted). The positions of conserved residues are indicated by blue spheres and green sticks. **d** Close up view of 'c' showing amino acid conservation between human and viral CD47-like receptors in the ^{117}SWF^{119} loop, inter-domain C15-C245 disulfide bond and in the HC1. The side chains of key conserved CD47 residues are shown as green sticks and labelled. Amino acid sequence alignments are provided in Supplementary Data 3 and 4. Source data is provided as a Source data file.

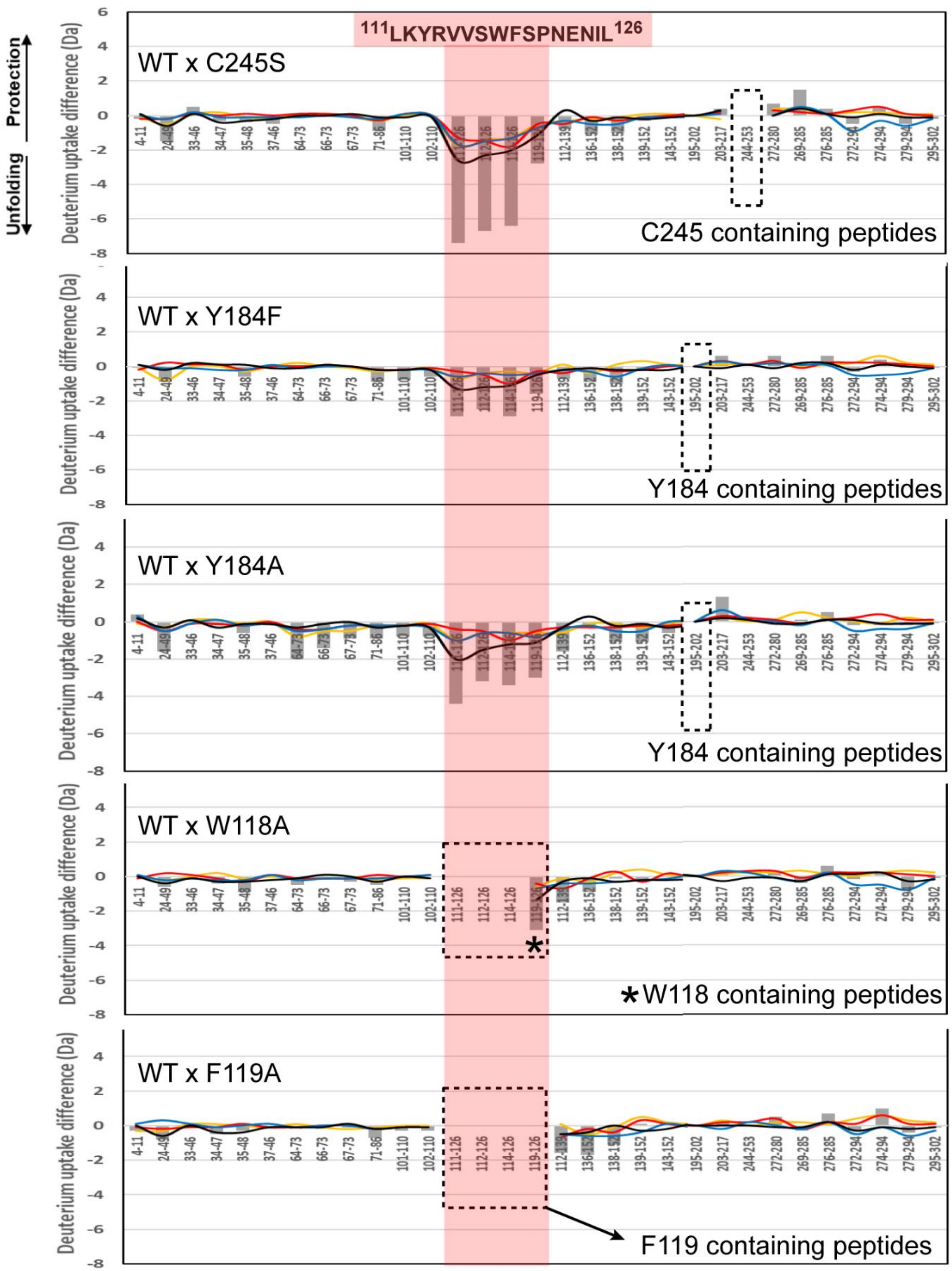

Supplementary Fig. 7 | Differential kinetic HDX-MS studies using wild type CD47^{BRIL} and mutants. Red box indicates peptides from the ^{114R}RVVSWF^{119} loop region and EC portion of helix I (111-126). The figure shows the differential HDX-MS data collected at the 60 min time point and grey boxes indicate average changes in HDX levels. Two independent experiments were performed. Black dotted boxes indicate the position of peptides containing single point mutations. Differential kinetic HDX-MS data are provided in Supplementary Data 5.

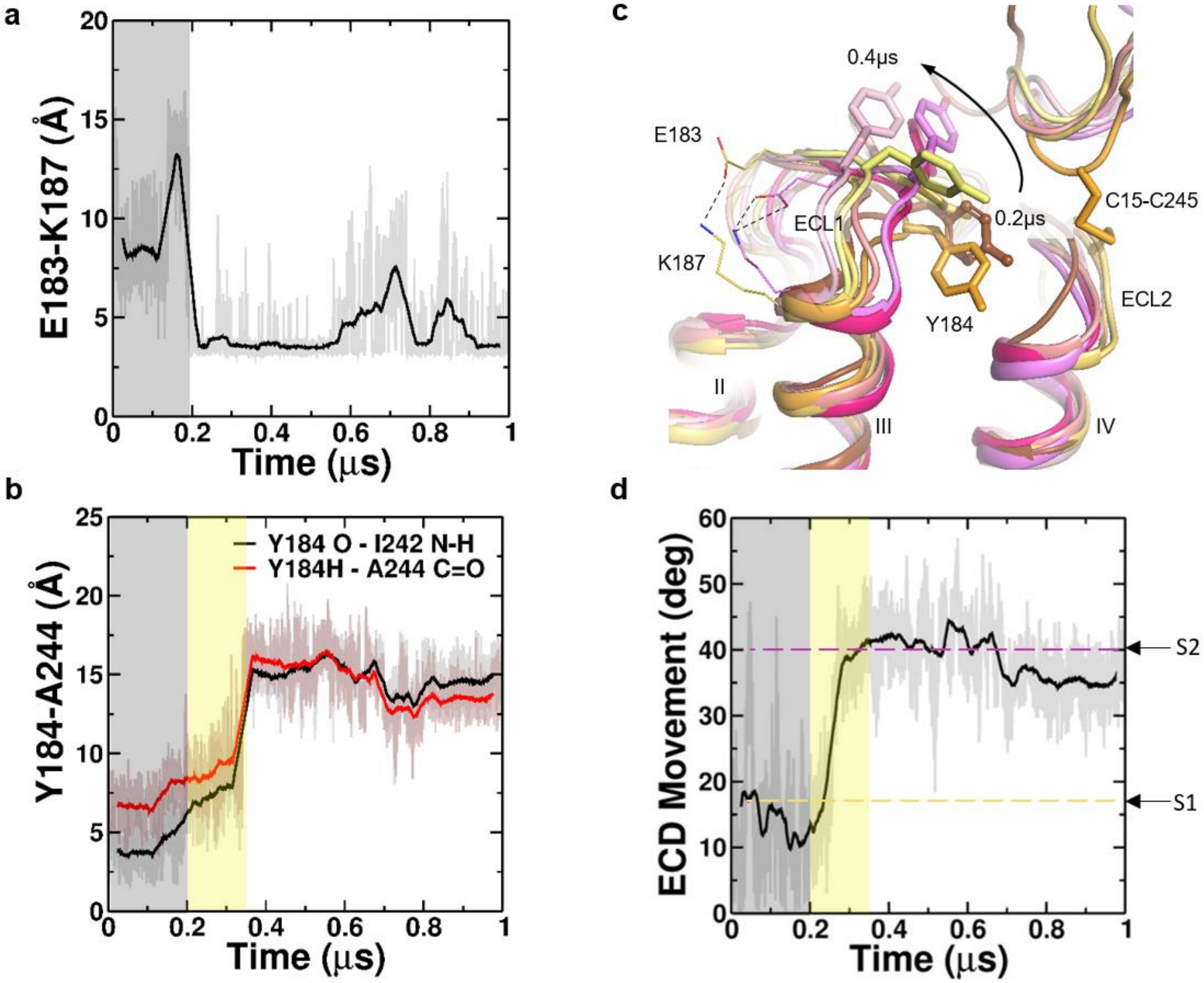

Supplementary Fig. 8 | Transition of CD47 ECD macrostates is associated to Y184 movement. a Plot showing the distance between E183 and K187 side chains during the course of the molecular dynamics simulations. The grey shaded area indicates the timescale required for the salt bridge formation. **b** Plot of the distance between Y184 side chain hydroxyl group to the main-chain carbonyl and amide atoms of A244 and I242 respectively. Grey shaded area indicates Y184 sampling s1 states (Y184 'in' position). The increase in the distance between atoms results in the transition to Y184 'out' position (yellow shaded area), favoring s2 states. **c** Representative CD47 models (cartoon with side chains showed as sticks) during the simulations showing the E183-K187 salt bridge (black dotted lines) formation as a precursor of Y184 egress from the large pocket. The Y184 'in' conformations (s1) are colored brown, orange and yellow; Y184 'out' conformations (s2) are shown in different shades of pink. **d** Average ECD movement with respect to the TMD during the simulations showing the ECD s1-s2 transition (yellow shaded area) occurring in the same timescale as the Y184 side chain switch to an 'out' position (panel 'b'). For plots in panels a, b and d: the grey and pink lines indicate the nanosecond fluctuations and the black and red lines the time average over every 1 ns within the single simulation trace.

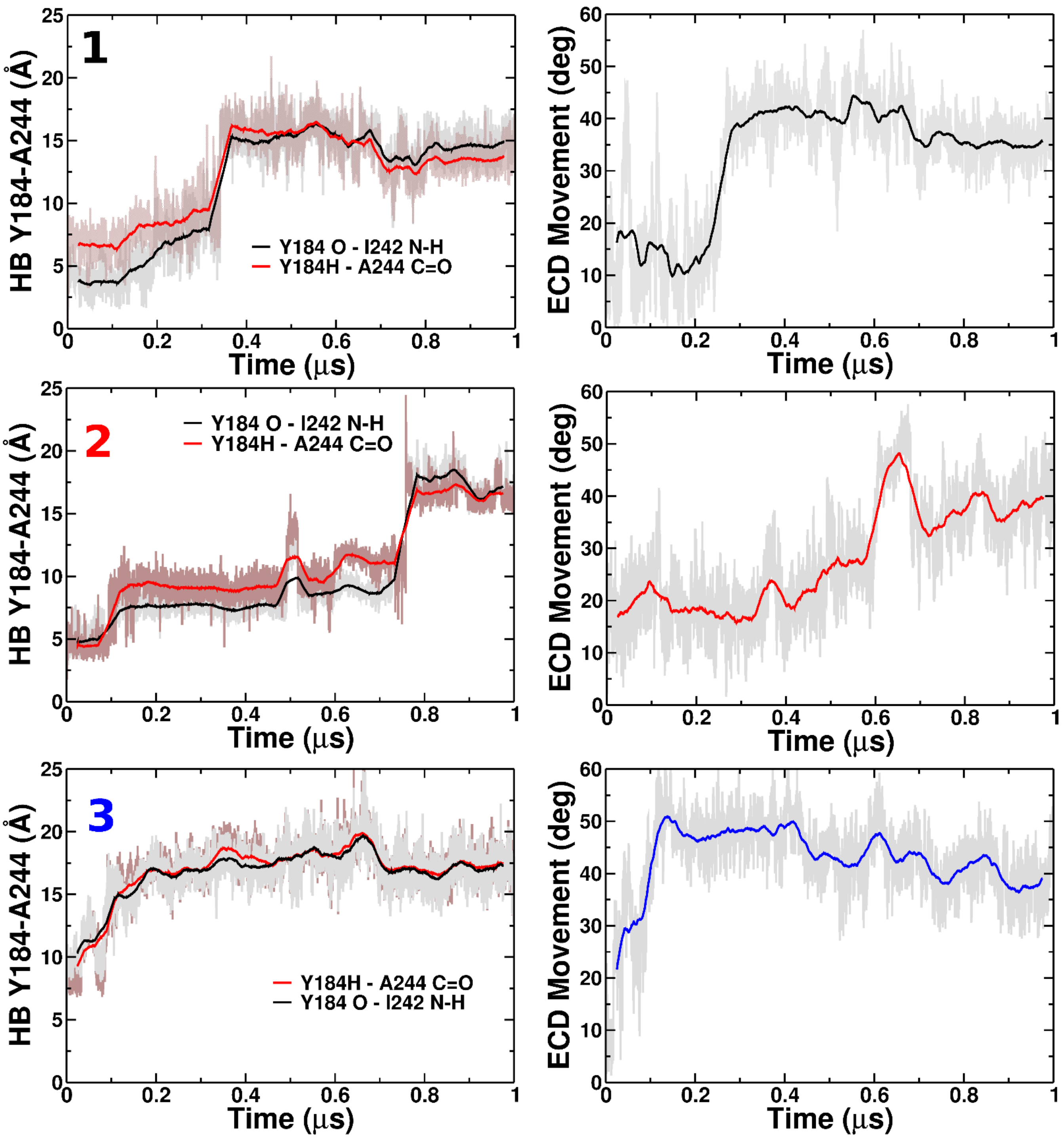

Supplementary Fig. 9 | CD47 ECD s1-s2 transition. Simulation replicates (1-3) of the ECD movement with respect to the TMD during the simulations. The ECD s1-s2 transition occurs in the same timescale as the Y184 side chain switch to an 'out' position. Left panels show the plot of the hydrogen bond (HB) distance between Y184 side chain hydroxyl group to the main-chain carbonyl and amide atoms of A244 and I242 respectively. The grey/pink lines indicate the nanosecond fluctuations and the black/red lines the time average over every 1 ns within the single simulation trace. The right panel shows the corresponding ECD movement during each simulation and the ECD s1-s2 transition.

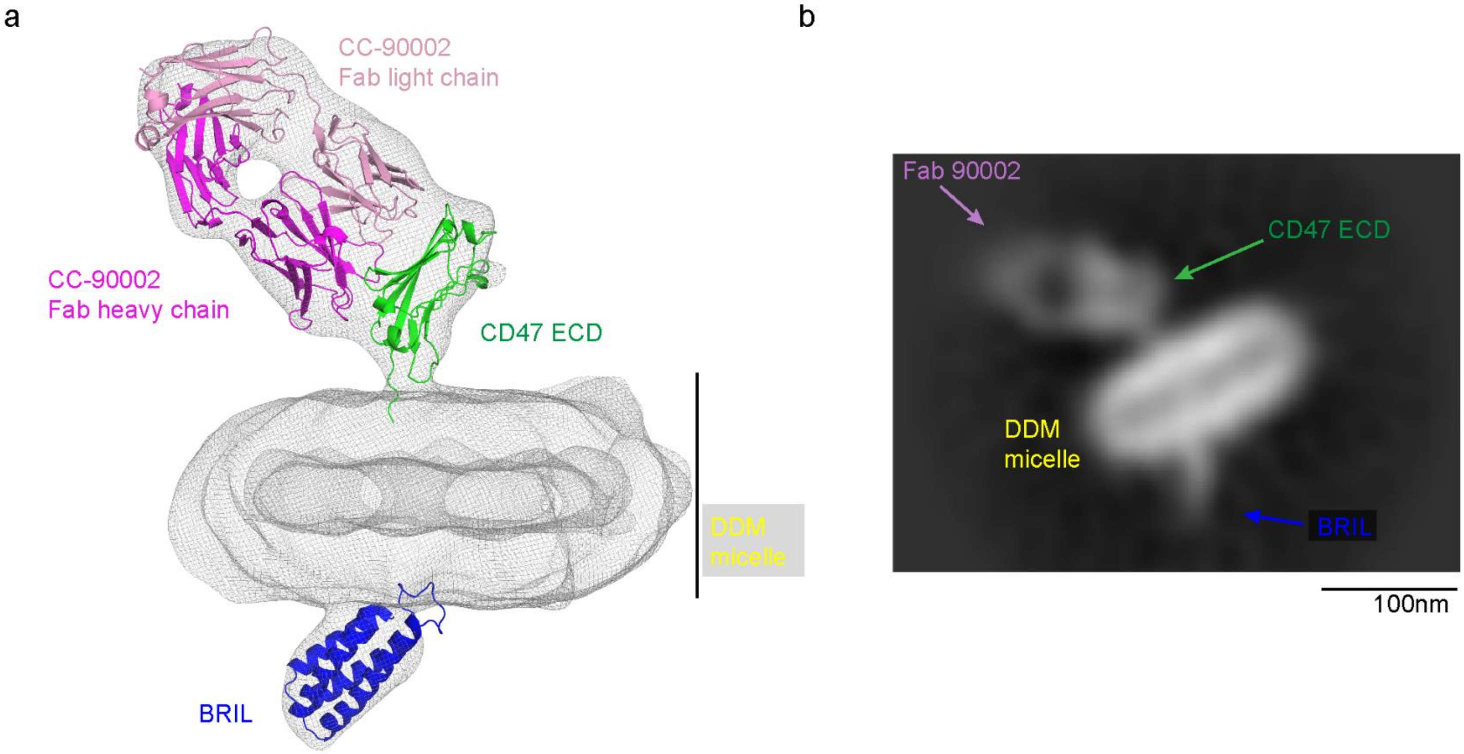

Supplementary Fig. 10 | Cryo-EM reconstruction of the full length CD47^{BRIL}-Fab CC-90002 complex. a A nominal 10Å resolution single particle Cryo-EM map for CD47^{BRIL} in complex with the Fab from CC-90002[7] mAb. The structure of the CD47-ECD in complex with the Fab CC-90002, and the BRIL fusion protein were independently fitted into the electron density map; low resolution hampered reconstruction and placement of the TMD into the electron density envelope. The micelle provides a reference plane mimicking the outer cell membranes. **b** Exemplary class average of the full length CD47^{BRIL}-Fab CC-90002 complex.

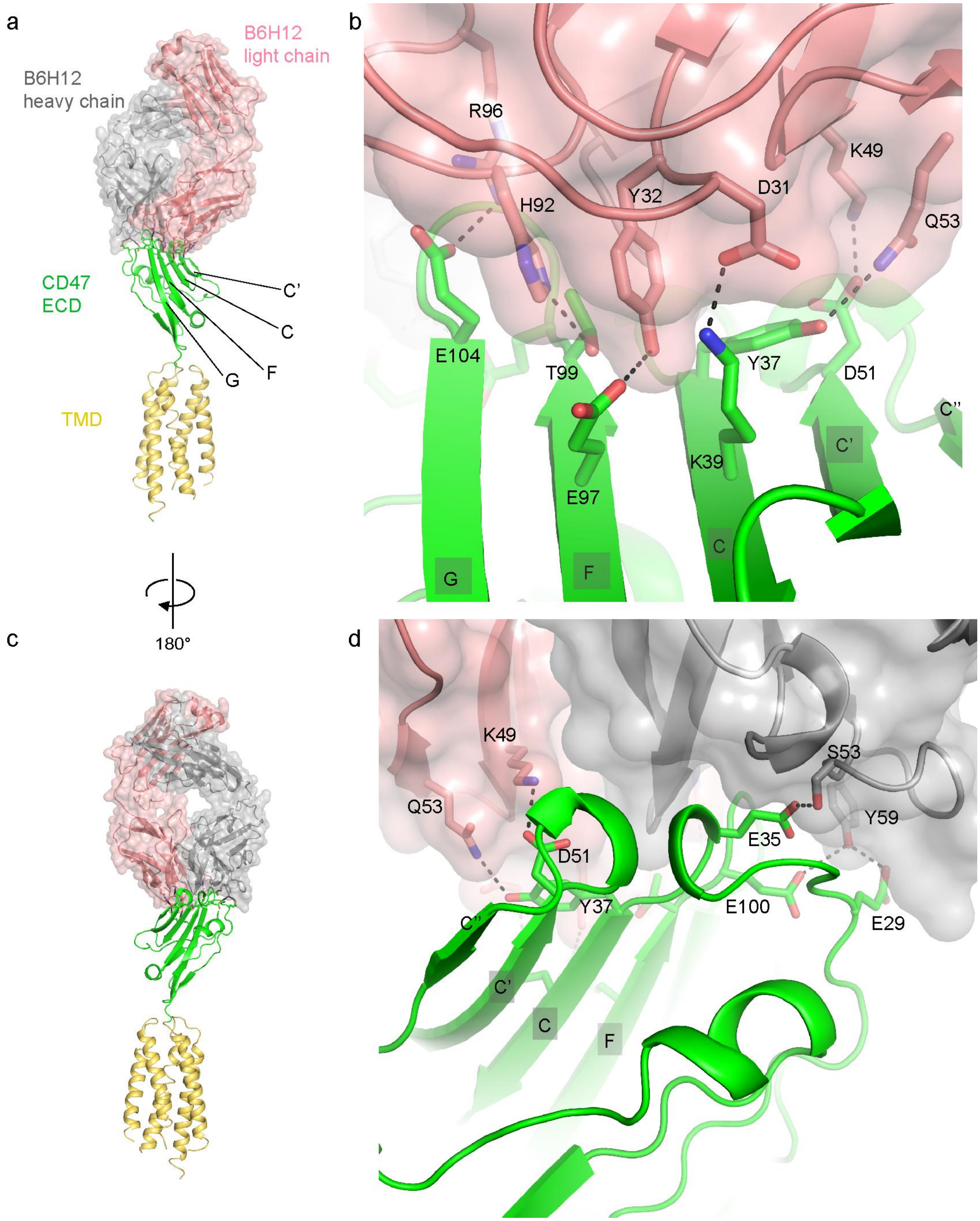

Supplementary Fig. 11 | The interface between the full length CD47 and Fab B6H12. a Overview of the CD47^{BRIL}-B6H12 crystal structure showing the receptor domains as a cartoon and the Fab B6H12 as a cartoon and surface representations. **b** Close view of the interface between CD47 ECD and the light chain of B6H12 (shown in pink) engaging the AGFCC'C" β-sheet. Hydrogen bond interactions between side chains (shown as sticks) are represented by black dotted lines. **c and d** Overview of the complex (180 degree rotation with respect to the position shown in **a**) and close up view of the hydrogen bonds between side chains in the CD47 ECD and B6H12 heavy chain (shown in grey) in the opposite face of the AGFCC'C" β-sheet.

Supplementary Table 1. | Diffraction data and refinement statistics for the CD47^{BRIL}-B6H12 complex

**Structure**	**CD47^{BRIL}-B6H12 (PDB ID 7MYZ)**
**Data collection**	**Diamond light source, 7µm x 7µm minibeam**
Number of crystals	49
Space group	$P2_1$
Cell dimensions	
a, b, c (Å)	141.53, 53.73, 181.7
α, β, γ (°)	90, 92.88, 90
Number reflections measured	118,336
Number of unique reflections	35,285 (2,866)
Resolution (Å)*	48.7-3.4 (3.5-3.4)
Wilson B-factor	80.25
CC1/2	(0.456)
CC*	(0.791)
$I / \sigma I$	3.8 (1.4)
Completeness (%)	91.65 (75.48)
Redundancy	3.3 (2.5)
**Refinement**	
Resolution (Å)	48.7-3.4
Number of reflections (test set)	1781
R_{work} / R_{free}	25.3/28.1
**No. atoms**	
Non-hydrogen	9239
Protein	9074
***B*-factors (Å^2)**	
CD47 ECDs (1-120)	101.7
CD47 TMD (121-278)	161.6
B6H12s	71.1
**R.m.s. deviations**	
Bond lengths (Å)	0.013
Bond angles (°)	1.83
**Ramachandran plot§**	
Favored (%)	94.0
Allowed (%)	5.0
Outliers (%)	1.0

*Values in parentheses are for highest-resolution shell.
§As defined in MolProbity[8]

Supplementary Table 2. | HDX-MS summary for WT CD47^{BRIL} and mutants.

Data Set	WILDTYPE	MUTANT C245S	MUTANT Y184F	MUTANT Y184A	MUTANT W118A	MUTANT F119A
HDX reaction details	10 mM phosphate buffer, 0.05% DDM, pD 7.0, 4 °C	10 mM phosphate buffer, 0.05% DDM, pD 7.0, 4 °C	10 mM phosphate buffer, 0.05% DDM, pD 7.0, 4 °C	10 mM phosphate buffer, 0.05% DDM, pD 7.0, 4 °C	10 mM phosphate buffer, 0.05% DDM, pD 7.0, 4 °C	10 mM phosphate buffer, 0.05% DDM, pD 7.0, 4 °C
HDX time course (min)	0.33, 1, 10, 60	0.33, 1, 10, 60	0.33, 1, 10, 60	0.33, 1, 10, 60	0.33, 1, 10, 60	0.33, 1, 10, 60
HDX control samples	N/A	N/A	N/A	N/A	N/A	N/A
Back-exchange (mean / IQR)	Not available					
# of Peptides	70	69	69	69	67	66
Sequence coverage	68%	65%	65%	65%	62%	61%
Average peptide length / Redundancy	13.07 / 3.66	13.20 / 3.74	13.09 / 3.73	13.09 / 3.73	13.16 / 3.69	13.25 / 3.78
Replicates (biological or technical)	2 (technical)	2 (technical)	2 (technical)	2 (technical)	2 (technical)	2 (technical)
Repeatability	0.128 (average standard deviation)	0.119 (average standard deviation)	0.127 (average standard deviation)	0.163 (average standard deviation)	0.204 (average standard deviation)	0.217 (average standard deviation)
Significant differences in HDX (delta HDX > XD)	0.671 D (99% CI)					

Supplementary References

1. Pietsch, E.C. et al. Anti-leukemic activity and tolerability of anti-human CD47 monoclonal antibodies. *Blood Cancer J* **7**, e536 (2017).
2. Afonine, P.V. et al. FEM: feature-enhanced map. *Acta Crystallogr D Biol Crystallogr* **71**, 646-66 (2015).
3. Liebschner, D. et al. Macromolecular structure determination using X-rays, neutrons and electrons: recent developments in Phenix. *Acta Crystallogr D Struct Biol* **75**, 861-877 (2019).
4. Pettersen, E.F. et al. UCSF Chimera--a visualization system for exploratory research and analysis. *J Comput Chem* **25**, 1605-12 (2004).
5. Ashkenazy, H. et al. ConSurf 2016: an improved methodology to estimate and visualize evolutionary conservation in macromolecules. *Nucleic Acids Res* **44**, W344-50 (2016).
6. Hatherley, D. et al. Paired receptor specificity explained by structures of signal regulatory proteins alone and complexed with CD47. *Mol Cell* **31**, 266-77 (2008).
7. Narla, R.K. et al. Modulation of CD47-SIRPalpha innate immune checkpoint axis with Fc-function detuned anti-CD47 therapeutic antibody. *Cancer Immunol Immunother* (2021).
8. Williams, C.J. et al. MolProbity: More and better reference data for improved all-atom structure validation. *Protein Sci* **27**, 293-315 (2018).
