# Supplementary material for: Structure of the human marker of self 5-transmembrane receptor CD47
Source: Nat Commun. 2021 Sep 1;12:5218. doi: 10.1038/s41467-021-25475-w (PMC8410850; doi:10.1038/s41467-021-25475-w)
Supplement: Supplementary file 3 — Description of Additional Supplementary Files [file 41467_2021_25475_MOESM3_ESM.docx]

Description of Additional Supplementary Files

**Supplementary Data 1:** Pairwise sequence alignment of all available mammalian CD47. Red boxes with a white character denote strict identity. Yellow boxes with red or black bold characters indicate similarities in consensus sequences. Source data are provided as a Source data file.

**Supplementary Data 2:** Pairwise sequence alignment of avian, reptile and human CD47. Red boxes with a white character denote strict identity. Yellow boxes with red or black bold characters indicate similarities in consensus sequences. Source data are provided as a Source data file.

**Supplementary Data 3:** Pairwise sequence alignment of CD47-like receptors from viruses and human CD47. Red boxes with a white character denote strict identity. Yellow boxes with red or black bold characters indicate similarities in consensus sequences. Source data are provided as a Source data file.

**Supplementary Data 4:** Pairwise sequence alignment of human CD47 and viral CD47-like receptors orthologues in the closest evolutionary branch to human CD47. Red boxes with a white character denote strict identity. Yellow boxes with red or black bold characters indicate similarities in consensus sequences. Source data are provided as a Source data file.

**Supplementary Data 5:** Kinetic hydrogen-deuterium exchange mass spectrometry (HDX-MS) analysis of CD47BRIL and mutants. Deuterium uptake is expressed as a percentage for each CD47BRIL peptide at different time points. Differential HDX was conducted based on the common peptides identified across all constructs covering 68% of WT CD47BRIL sequence. Each value represents the mean value across two replicates of a representative experiment. Error bars represent the ± S.D. of duplicate measurements. At least two independent experiments were performed with the full panel of receptor mutants. Peptides or residues corresponding to the BRIL fusion protein are shown in grey.

**Supplementary Data 6:** Kinetic hydrogen-deuterium exchange mass spectrometry (HDX-MS) plots of CD47BRIL and mutants. The deuterium uptake for each CD47BRIL peptide at different time points is shown. Differential HDX was conducted based on the common peptides identified across all constructs covering 68% of WT CD47BRIL sequence. Each value represents the mean value across two replicates of a representative experiment. The values of each duplicate measurement are shown. At least two independent experiments were performed with the full panel of receptor mutants. Peptides corresponding to the BRIL fusion protein are omitted for clarity.
