# Supplementary material for: Structure of the human marker of self 5-transmembrane receptor CD47
Source: Nat Commun. 2021 Sep 1;12:5218. doi: 10.1038/s41467-021-25475-w (PMC8410850; doi:10.1038/s41467-021-25475-w)
Supplement: Supplementary file 4 — Supplementary Data 1 [file 41467_2021_25475_MOESM4_ESM.pdf]

```
1 10
CD47_HUMAN ..........................................................................QLLFNKTKSVEFTF
Northern_greater_galago ..............................TV.TV...........................LS.ASCA.RSAQLLFNKTKSVEYTV
Coquerels_sifaka .....M.......................WPLVAA.LL...........................LA.SACC.GSAQLLFNKTKSVEFTF
Common_marmoset .....M.......................WPLVAA.LL...........................LG.SACC.GSAQLLFNKTKSVEFTF
Black-capped_squirrel_monkey .....M.......................KC.......................................FS.FSFP.GSAQLLFNKTKSVEFTF
Panamanian_white-faced_capuchin .....M.......................WPLVAA.LL...........................LG.SACC.GSAQLLFNKTKSVEFTF
Nancy_Mas_night_monkey .....M.......................WPLVAA.LL...........................LG.SACC.GSAQLLFNKTKSVEFTF
Black_snub-nosed_monkey .....M.......................KY.......................................FS.FSFP.GSAQLLFNKTKSVEFTF
Golden_snub-nosed_monkey .....M.......................WPLVAA.LL...........................LG.SACC.GSAQLLFNKTKSVEFTF
Angolan_Colobus ...........................................................................SAQLLFNKTKSVEFTF
Rhesus_macaque .....M.......................WPLVAA.LL...........................LG.SACC.GSAQLLFNKTKSVEFTF
Green_monkey .....M.......................WPLVAA.LL...........................LG.SACC.GSAQLLFNKTKSVEFTF
Sooty_mangabey .....M.......................WPLVAA.LL...........................LG.SACC.GSAQLLFNKTKSVEFTF
Olive_baboon .....M.......................WPLVAA.LL...........................LG.SACC.GSAQLLFNKTKSVEFTF
Crab-eating_macaque .....M.......................WPLVAA.LL...........................LG.SACC.GSAQLLFNKTKSVEFTF
Southern_pig-tailed_macaque .....M.......................WPLVAA.LL...........................LG.SACC.GSAQLLFNKTKSVEFTF
Drill ...........................................................................SAQLLFNKTKSVEFTF
Northern_white-cheeked_gibbon .....M.......................WPLVAA.LL...........................LG.SACC.GSAQLLFNKTKSVEFTF
Sumatran_orangutan .....M.......................WPLVAA.LL...........................LG.SACC.GSAQLLFNKTKSVEFTF
Western_gorilla .....M.......................WPLVAA.LL...........................LG.SAXX.XSAQLLFNKTKSVEFTF
Chimpanzee .....M.......................WPLVAA.LL...........................LG.SACC.GSAQLLFNKTKSVEFTF
Bonobo ...........................................................................SAQLLFNKTKSVEFTF
African_elephant .....A...........................LL.II...........................LG.ICSI.CSAQITFNVIKSVEYTV
Golden_hamster .....M.......................WPLVAA.LL...........................LG.SCCC.GSAQLLFSKVKSAEFTT
Mouse .....M.......................WPLAAA.LL...........................LG.SCCC.GSAQLLFSNVNSIEFTS
Rat .....M.......................WPLAAA.LL...........................LG.SCCC.GSAQLLLSKVKSVEFTS
Guinea_pig .....M.......................WPLVAT.LL...........................LG.SWCF.GSAQLLLNKTKLVEYTI
Naked_mole_rat .....M.......................WPLAAT.LL...........................LG.SWCF.GSAQLLLNKTKFVEYTI
American_beaver .....M.......................WPLVAA.LL...........................LG.SACC.GSAQLLLNNTKSVEYTA
Ords_kangaroo_rat .....M.......................WPLVAA.LL...........................LG.SSCI.GSAQLLFDKTRLVEYTS
Horse .....M.......................WPLVLL.LL...........................LG.SACC.GSAQLIFNVIKSVEYTV
Western_European_hedgehog .....M.......................WPLVVA.LL...........................LG.SACC.GSAQLIFNVTKSVEYTI
Cat .....M.......................WPLVVL.LLL...........................G.SACC.GSAQLIFNITRSVEYTA
Weddell_seal .....M.......................LPLVV..LL...........................LG.SACC.GSAQLIFNITKSVEYTA
Dog .....M.......................WPLAAL.LLL...........................G.SASC.GSAQLIFNITKSVEYTA
Red_fox .....A.......................A..GAP.LLA...........................P.LFFL.GSAQLIFNITKSVEYTA
Hawaiian_monk_seal .....M.......................LPLVV..LL...........................LG.SACC.GSAQLIFNITKSVEYTA
American_black_bear .....M.......................WPLVV..LL...........................LG.SACC.GSAQLIFNITKSVEYTA
Grizzly_bear .....M.......................WPLVV..LL...........................LG.SACC.GSAQLIFNITKSVEYTA
Northern_fur_seal .....M.......................LPLVV..LL...........................LG.SACC.GSAQLIFNITKSVEYTA
Pacific_walrus .....M.......................LPLVV..LL...........................LG.SACC.GSAQLIFNITKSVEYTA
Giant_panda .....Q.......................FPMFA..CLL..........................CSCTICL.SSAQLIFNITKSVEYTA
Polar_bear .....M.......................FTSTT..ELFE.......EPCS...........K.KEN.SNEQ.GSAQLIFNITKSVEYTA
Europen_domestic_ferret .........................................................................GSAQLIFNITKSVEYTA
Sea_otter .....M.......................LPLVV..LL...........................LG.SASC.GSAQLIFNITKSVEYTA
Little_brown_bat .....L.......................WCFFVLFCF...........................LV.SMKCGSSAQLIFNITKFAEYTI
Hybrid_cattle .....MERGEKCGAAEVPSRGRTVDEGRGEPLWA...P...........................EG.SGLR.GSAQLIFNAIKSVEYTL
Cow .....M.......................WPLVVV.LL...........................LG.SVRC.GSAQLIFNAIKSVEYTL
Sheep .....M..............................................................ER.GSAQLIFNVIKSVEYTL
Goat .....M.......................WPLVVV.LL...........................LA.SVRC.GSAQLIFNVIKSVEYTL
Pig .....M.......................WPLVVV.VL...........................LG.SAYC.GSAQLIFNITKSVEFTV
Atlantic_bottle-nosed_dolphin .....M.......................WPLVVL.LL...........................LG.SARC.GSAQLIFNTTKSVEYTV
Yangtze_finless_porpoise .....M.......................WPLVVV.LL...........................LG.SARC.GSAQLIFNATKSVEYTI
Yangtze_river_dolphin .....M.......................WPLVVL.LL...........................LG.TARC.GSAQLIFNTTKSVEYTV
Beluga_whale .....M.......................WPLVVL.LL...........................LG.SARC.GSAQLIFNTTKSVEYTV
North_pacific_minke_whale .....M.......................WPLVVL.LL...........................LG.SARC.GSAQLIFNTTKSVEYTV
Sperm_whale .....M.......................WPLVVL.LL...........................LG.SARC.GSAQLIFNTTKSVEYTV
Duckbill .....A.......................WPYVKF.FS...........................CF.FALP.GSAQLLFDSPRLVEKNA
Common .....M.......................WSLVAA.LL...........................LG.TVCG.GSAQLTFNSTRFVEETI
Tasmanian .........................................................................GSAQLTFNSTRFVEETI
Indian_muntjac .....M.......................E.......................................DNY.SSAQLIFNVIKSVEYTV
Reevess_muntjac .....M.......................E.......................................DNY.SSAQLIFNVIKSVEYTV
Domestic_yak .....M.......................ESEPCA.FRFHGMIGDVGKSRCSEEGKVEARSFLHCAV.SSEG.SSAQLIFNAIKSVEYTL
Spotted_hyena .........................................................................GSAQLIFNITRSVEYTA
Iberian_lynx .....M.......................KQF....................................S.FSFS.GSAQLIFNITRSVEYTA
Meerkat MAAAKM.......................WPLVV..LLL...........................G.SAGC.GSAQLIFNITRSVEYTA
Canada_lynx .....M.......................WPLVVL.LLL...........................G.SACC.GSAQLIFNITRSVEYTA
Greater_horseshoe_bat .....M.......................WPLVVV.LL...........................LN.SAYC.GSAQLILNVTKFVEYTN
Groundhog .....M.......................WPLVAA.LL...........................LG.SFCS.GSAQLLFNKTKSVEFTF
West_Indian_manatee .....M.......................WPLVGA.LL...........................LG.SACC.GSAQITFNITKSAEFTV
American_mink .....M.......................LPLVV..LL...........................LG.SASC.GSAQLIFNITRSVEYTA
European_rabbit .....M.......................WPLVAA.LL...........................LG.SACC.GSAQLIFNKTKSVEFTF
consensus>70 .....m........................pl.....l..............................s....gsaQl.fn.tksvEyt.
```

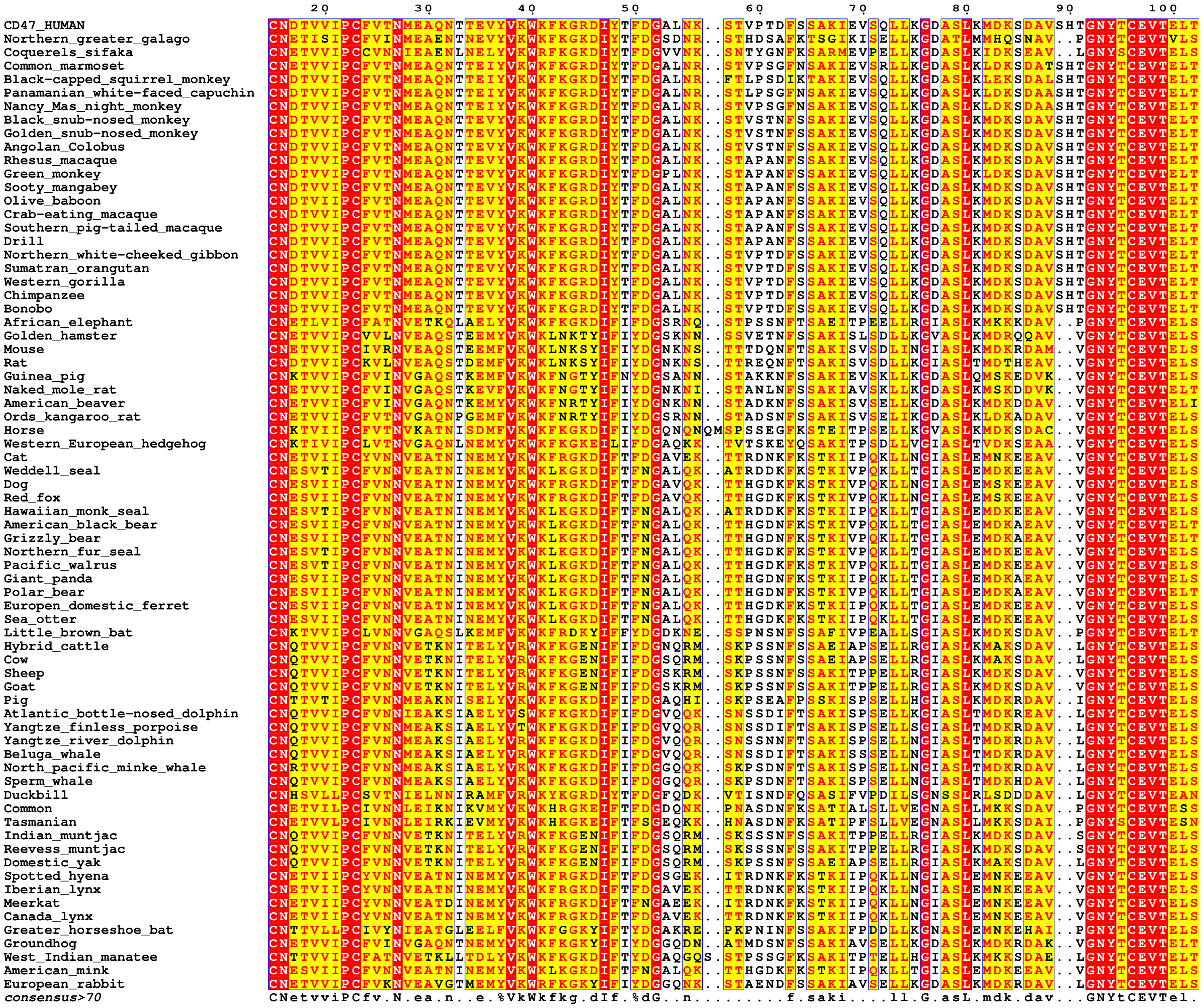
20 30 40 50 60 70 80 90 100
CD47_HUMAN CNDTVVIPCFVTNMEAQNTTEVYVKWKFKGRDIYTFDGALNK..STVPTDFSSAKIEVSQLLKGDASLKMDKSDAVSHTGNYTCEVTELT
Northern_greater_galago CNETISIPCFVINMEAENTNEVYVKWKFKGKDIYTFDGSDNR..STHDSAFKTSGIKISELLKGDATLMMHQSNAV..PGNYTCEVTVLS
Coquerels_sifaka CNETVVIPCCVNNIEAENLNELYVKWRFKGKDIYTFDGVVNK..SNTYGNFKSARMEVPELLKGDASLKIDKSEAV..LGNYSCEVTELS
Common_marmoset CNETVVIPCFVTNMEAQNTTEIYVKWKFKGRDIYTFDGALNR..STVPSGFNSAKIEVSRLLKGDASLKLDKSDATSHTGNYTCEVTELT
Black-capped_squirrel_monkey CNDTVVIPCFVTNMEAQNTTEIYVKWKFKGRDIYTFDGALNR..FTLPSDIKTAKIEVSQLLKGDASLKLEKSDALSHTGNYTCEVTELT
Panamanian_white-faced_capuchin CNDTVVIPCFVTNMEAQNTTEIYVKWKFKGRDIYTFDGALNR..STLPSGFNSAKIEVSQLLKGDASLKLDKSDAASHTGNYTCEVTELT
Nancy_Mas_night_monkey CNDTVVIPCFVTNMEAQNTTEIYVKWKFKGRDIYTFDGALNR..STVPSGFNSAKIEVSQLLKGDASLKLDKSDAASHTGNYTCEVTELT
Black_snub-nosed_monkey CNDTVVIPCFVTNMEAQNTTEVYVKWKFKGRDIYTFDGALNK..STVSTNFSSAKIEVSQLLKGDASLKMDKSDAVSHTGNYTCEVTELT
Golden_snub-nosed_monkey CNDTVVIPCFVTNMEAQNTTEVYVKWKFKGRDIYTFDGALNK..STVSTNFSSAKIEVSQLLKGDASLKMDKSDAVSHTGNYTCEVTELT
Angolan_Colobus CNDTVVIPCFVTNMEAQNTTEVYVKWKFKGRDIYTFDGALNK..STVSTNFSSAKIEVSQLLKGDASLKMDKSDAVSHTGNYTCEVTELT
Rhesus_macaque CNDTVVIPCFVTNMEAQNTTEVYVKWKFKGRDIYTFDGALNK..STAPANFSSAKIEVSQLLKGDASLKMDKSDAVSHTGNYTCEVTELT
Green_monkey CNDTVVIPCFVTNMEAQNTTEVYVKWKFKGRDIYTFDGPLNK..STAPANFSSAKIEVSQLLKGDASLKLDKSDAVSHTGNYTCEVTELT
Sooty_mangabey CNDTVVIPCFVTNMEAQNTTEVYVKWKFKGRDIYTFDGALNK..STAPANFSSAKIEVSQLLKGDASLKMDKSDAVSHTGNYTCEVTELT
Olive_baboon CNDTVIIPCFVTNMEAQNTTEVYVKWKFKGRDIYTFDGALNK..STAPANFSSAKIEVSQLLKGDASLKMDKSDAVSHTGNYTCEVTELT
Crab-eating_macaque CNDTVVIPCFVTNMEAQNTTEVYVKWKFKGRDIYTFDGALNK..STAPANFSSAKIEVSQLLKGDASLKMDKSDAVSHTGNYTCEVTELT
Southern_pig-tailed_macaque CNDTVVIPCFVTNMEAQNTTEVYVKWKFKGRDIYTFDGALNK..STAPANFSSAKIEVSQLLKGDASLKMDKSDAVSHTGNYTCEVTELT
Drill CNDTVVIPCFVTNMEAQNTTEVYVKWKFKGRDIYTFDGALNK..STAPANFSSAKIEVSQLLKGDASLKMDKSDAVSHTGNYTCEVTELT
Northern_white-cheeked_gibbon CNDTVVIPCFVTNMEAQNTTEVYVKWKFKGRDIYTFDGALNK..STVPTDFSSAKIEVSQLLKGDASLKMDKSDAVSHTGNYTCEVTELT
Sumatran_orangutan CNDTVVIPCFVTNMEAQNTTEVYVKWKFKGRDIYTFDGALNK..STVPTDFSSAKIEVSQLLKGDASLKMDKSDAVSHTGNYTCEVTELT
Western_gorilla CNDTVVIPCFVTNMEAQNTTEVYVKWKFKGRDIYTFDGALNK..STVPTDFSSAKIEVSQLLKGDASLKMDKSDAVSHTGNYTCEVTELT
Chimpanzee CNDTVVIPCFVTNMEAQNTTEVYVKWKFKGRDIYTFDGALNK..STVPTDFSSAKIEVSQLLKGDASLKMDKSDAVSHTGNYTCEVTELT
Bonobo CNDTVVIPCFVTNMEAQNTTEVYVKWKFKGRDIYTFDGALNK..STVPTDFSSAKIEVSQLLKGDASLKMDKSDAVSHTGNYTCEVTELT
African_elephant CNETLVIPCFATNVETKQLAELYVKWKFKGKDIFIFDGSRNQ..STPSSNFTSAEITPEELLRGIASLKMKKKDAV..PGNYTCEVTELS
Golden_hamster CNETVVIPCVVLNVEAQSTEEMYVKWKLNKTYIFIYDGSKNN..SSVETNFSSAKISLSDLLKCVASLKMDRQQAV..VGNYTCEVTELS
Mouse CNETVVIPCIVRNVEAQSTEEMFVKWKLNKSYIFIYDGNKNS..TTTDQNFTSAKISVSDLLNGIASLKMDKRDAM..VGNYTCEVTELS
Rat CNDTVVIPCKVLNVEAQSTDEMFVKWKLNKSYIFIYDGNKNS..TTREQNFTSAKISVSDLLKGIASLTMDTHEAV..VGNYTCEVTELS
Guinea_pig CNKTVVIPCFVINVGAQSTKEMFVKWKFNGTYIFNYDGSANN..STAKKNFSSAKIEVSKLLKGDASLQMSKEDVK..VGNYTCEVTELS
Naked_mole_rat CNETVVIPCFVINVGAQSTKEVFVKWKFNGTYIFIYDGNKNI..STANLNFSSAKIAVSKLLKGDASLKMSKDDVK..VGNYTCEVTELT
American_beaver CNETVVIPCFVINVGAQNTKEMYVKWKFNRTYIFIYDGNKNN..STADKNFSSAKISVSELLKGDASLKMDKDDAV..VGNYTCEVTELI
Ords_kangaroo_rat CNETVVIPCFVTNVGAQNPGEMFVKWKFNRTYIFIYDGSRNN..STADSNFSSAKISVSELIKGDASLKLDKADAV..VGNYTCEVTELS
Horse CNKTVIIPCFVTNVKATNISDMFVKWKFKGKDIFIYDGQNQNQMSPSSEGFKSTEITPSELLKGVASLKMDKSDAC..VGNYTCEVTELS
Western_European_hedgehog CNKTIVIPCLVTNVGAQNLNEMYVKWKFKGKEILIFDGAQKK..TVTSKEYQSAKITPSDLLVGIASLTVDKSEAA..VGNYTCEVTELS
Cat CNETVIIPCYVNNVEATNINEMYVKWKFRGKDIFTFDGAVEK..TTRDNKFKSTKIIPQKLLNGIASLEMNKEEAV..VGNYTCEVTELS
Weddell_seal CNESVTIPCFVNNVEATNINEMYVKWKLKGKDIFTFNGALQK..ATRDDKFKSTKIVPQKLLTGIASLEMDKEEAV..VGNYTCEVTELS
Dog CNESVIIPCFVNNVEATNINEMYVKWKFRGKDIFTFDGAVQK..TTHGDKFKSTKIVPQKLLNGIASLEMSKEEAV..VGNYTCEVTELS
Red_fox CNESVIIPCFVNNVEATNINEMYVKWKFRGKDIFTFDGAVQK..TTHGDKFKSTKIVPQKLLNGIASLEMSKEEAV..VGNYTCEVTELS
Hawaiian_monk_seal CNESVTIPCFVNNVEATNINEMYVKWKLKGKDIFTFNGALQK..ATRDDKFKSTKIIPQKLLTGIASLEMDKEEAV..VGNYTCEVTELS
American_black_bear CNESVIIPCFVNNVEATNINEMYVKWKLKGKDIFTFNGALQK..TTHGDNFKSTKIVPQKLLTGIASLEMDKAEAV..VGNYTCEVTELT
Grizzly_bear CNESVIIPCFVNNVEATNINEMYVKWKLKGKDIFTFNGALQK..TTHGDNFKSTKIVPQKLLTGIASLEMDKAEAV..VGNYTCEVTELT
Northern_fur_seal CNESVTIPCFVNNVEATNINEMYVKWKLKGKDIFTFNGALQK..TTHGDKFKSTKIVPQKLLTGIASLEMDKEEAV..VGNYTCEVTELS
Pacific_walrus CNESVTIPCFVNNVEATNINEMYVKWKLKGKDIFTFNGALQK..TTHGDKFKSTKIVPQKLLTGIASLEMDKEEAV..VGNYTCEVTELS
Giant_panda CNESVIIPCFVNNVEATNINEMYVKWKLKGKDIFTFNGALQK..TTHGDNFKSTKIIPQKLLTGIASLEMDKAEAV..VGNYTCEVTELS
Polar_bear CNESVIIPCFVNNVEATNINEMYVKWKLKGKDIFTFNGALQK..TTHGDNFKSTKIVPQKLLTGIASLEMDKAEAV..VGNYTCEVTELT
Europen_domestic_ferret CNESVIIPCFVNNVEATNINEMYVKWKLKGKDIFTFNGALQK..TTHGDKFKSTKIIPQKLLTGIASLEMDKEEAV..VGNYTCEVTELS
Sea_otter CNESVIIPCFVNNVEATNINEMYVKWKLKGKDIFTFNGALQK..TTHGDKFKSTKIIPQKLLTGIASLEMDKEEAV..VGNYTCEVTELS
Little_brown_bat CNKTVVIPCLVNNVGAQSLKEMFVKWKFRDKYIFFYDGDKNE..SSPNSNFSSAFIVPEALLSGIASLKMDKSDAV..PGNYTCEVTELT
Hybrid_cattle CNQTVVIPCFVNNVETKNITELYVRWKFKGENIFIFDGNQRM..SKPSSNFSSAEIAPSELLRGIASLKMAKSDAV..LGNYTCEVTELS
Cow CNQTVVIPCFVNNVETKNITELYVRWKFKGENIFIFDGSQRM..SKPSSNFSSAEIAPSELLRGIASLKMAKSDAV..LGNYTCEVTELS
Sheep CNQTVVIPCFVNNVETKNITELYVRWKFKGENIFIFDGSKRM..SKPSSNFSSAKITPPELLRGIASLKMDKSDAV..VGNYTCEVTELS
Goat CNQTVVIPCFVNNVETKNITELYVRWKFKGENIFIFDGSKRM..SKPSSNFSSAKITPPELLRGIASLKMDKSDAV..VGNYTCEVTELS
Pig CNTTVTIPCFVNNMEAKNISELYVKWKFKGKDIFIFDGAQHI..SKPSEAFPSSKISPSELLHGIASLKMDKRDAV..IGNYTCEVTELS
Atlantic_bottle-nosed_dolphin CNQTVVIPCFVNNIEAKSIAELYVSWKFKGKDIFIFDGVQQK..SNSSDIFTSAKISPSELLKGIASLTMDKREAV..LGNYTCEVTELS
Yangtze_finless_porpoise CNQTVVIPCFVNNMEAKSIAELYVTWKFKGKDIFIFDGVQQR..SNSSDIFTSAKISPSELLSGIASLTMDKRDAV..LGNYTCEVTELS
Yangtze_river_dolphin CNQTVVIPCFVNNMEAKSIAELYVRWKFKGKDIFIFDGVQQR..SNSSNNFTSAKISPSELLNGIASLTMDKRDAV..LGNYTCEVTELS
Beluga_whale CNQTVVIPCFVNNMEAKSIAELYVRWKFKGKDIFIFDGVQQR..SNSSDIFTSAKISSSELLNGIASLTMDKRDAV..LGNYTCEVTELS
North_pacific_minke_whale CNRTVVIPCFVNNMEAKSIAELYVRWKFKGKDIFIFDGGQQK..SKPSDNFTSAKISPSELLNGIASLTMDKRDAV..LGNYTCEVTELS
Sperm_whale CNQTVVIPCFVNNMEAKSIAELYVRWKFKGKDIFIFDGGQQR..SKPSDNFTSAKISPSELLNGIASLTMDKHDAV..LGNYTCEVTELS
Duckbill CNHSVLLPCSVTNIELNNIRAMFVRWKYKGRDIYTFDGFQDK..VTISNDFQSASIFVPDILSGNSSLRLSDDDAV..LGNYTCEVTEAN
Common CNETVILPCIVNNLEIKNIKVMYVKWKHRGKEIFTFDGDQNK..PNASDNFKSATIALSLLVEGNASLLMKKSDAV..PGNYTCEVTESS
Tasmanian CNETVVLPCIVNNLEIRKIEVMYVKWKHKGKEIFTFSGEQKK..HNASDNFKSATIPFSLLVEGNASLLMKKSDAI..PGNYSCEVTESN
Indian_muntjac CNQTVVIPCFVNNVETKNITELYVRWKFKGENIFIFDGSQRM..SKSSSNFSSAKITPPELLRGIASLKMDKSDAV..SGNYTCEVTELS
Reevess_muntjac CNQTVVIPCFVNNVETKNITELYVRWKFKGENIFIFDGSQRM..SKSSSNFSSAKITPPELLRGIASLKMDKSDAV..SGNYTCEVTELS
Domestic_yak CNQTVVIPCFVNNVETKNITELYVRWKFKGENIFIFDGSQRM..SKPSSNFSSAEIAPSELLRGIASLKMAKSDAV..LGNYTCEVTELS
Spotted_hyena CNETVIIPCYVNNVEATNINEMYVKWKFRGKDIFTFDGSGEK..ITRDNKFKSTKIIPQKLLNGIASLEMNKEEAV..VGNYTCEVTELS
Iberian_lynx CNETVIIPCYVNNVEATNINEMYVKWKFRGKDIFTFDGAVEK..TTRDNKFKSTKIIPQKLLNGIASLEMNKEEAV..VGNYTCEVTELS
Meerkat CNETVIIPCYVNNVEATDINEMYVKWKFRGKDIFTFNGAEEK..ITRDNKFKSTKIIPQKLLNGIASLEMNKEEAV..VGNYTCEVTELS
Canada_lynx CNETVIIPCYVNNVEATNINEMYVKWKFRGKDIFTFDGAVEK..TTRDNKFKSTKIIPQKLLNGIASLEMNKEEAV..VGNYTCEVTELS
Greater_horseshoe_bat CNTTVLLPCIVYNIEATGLEELFVKWKFGGKYIFTYDGAKRE..PKPNINFSSAKIFPDDLLKGNASLEMNKEHAI..PGNYTCEVTELS
Groundhog CNETVVIPCFVINVGAQNTNEMYVKWKFKGKYIFIYDGGQDN..ATMDSNFSSAKIAVSELLKGDASLKMDKRDAK..VGNYTCEVTELT
West_Indian_manatee CNTTVVIPCFATNVETKLLTDLYVKWKFKGKDIFTYDGAQGQS.STPSSGFKSAKITPTELLHGIASLKMKKSDAE..LGNYTCEVTELT
American_mink CNESVIIPCFVNNVEATNINEMYVKWKLKGKDIFTFNGALQK..TTHGDKFKSTKIIPQKLLTGIASLEMDKEEAV..VGNYTCEVTELS
European_rabbit CNETVVIPCFVKNVEAVGTMEMYVKWRFKGKYIFIYDGGQNK..STAENDFSSAKIAVSELLKGDASLKMDKRDAV..LGNYTCEVTELS
consensus>70 CNetvviPCfv.N.ea.n..e.%VkWkfkg.dIf.%dG..n........f.saki....ll.G.asL.mdk.dav...GNYtCEVTel.

```
                                 110                             120       130       140       150       160
CD47_HUMAN                       REGETIIELKYRV.........................VSWFSPNENILIVIFPIFAILLFWGQFGIKTLKYRSGGMDEKTIALLVAGLV
Northern_greater_galago          REGETTIEVKYRI.........................VSWFSPNENILIVIFPVFALLLFWGQFGIKTLQYRSGRMDEKTIGLLVAGLL
Coquerels_sifaka                 REGETFIELKYRV.........................VPWFSPNENILIVIFPIFAVLLFWGQFGIKTLKYKSSHMDEKTIAVLVAGLV
Common_marmoset                  REGETIVELKYRVGTLGDRWKSCHLTNKERKEIEGIVQVSWFSPNENILIVIFPIFAILLFWGQFGIKTLKYRSGGMDEKTIALLVAGLT
Black-capped_squirrel_monkey     REGETIIELKYRV.........................VSWFSPNENILIVIFPIFAILLFWGQFGIKTLKYRSGGMDEKTIALLVAGLT
Panamanian_white-faced_capuchin  REGETIVELKYRV.........................VSWFSPNENILIVIFPIFAILLFWGQFGIKTLKYRSGGMDEKTIALLVAGLT
Nancy_Mas_night_monkey           REGETIVELKYRV.........................VSWFSPNENILIVIFPIFAILLFWGQFGIKTLKYRSGGMDEKTIALLVAGLT
Black_snub-nosed_monkey          REGETIIELKYRV.........................VSWFSPNENILIVIFPIFAILLFWGQFGIKTLKYRSGGMDEKTIALLVAGLM
Golden_snub-nosed_monkey         REGETIIELKYRV.........................VSWFSPNENILIVIFPIFAILLFWGQFGIKTLKYRSGGMDEKTIALLVAGLM
Angolan_Colobus                  REGETIIELKYRV.........................VSWFSPNENILIVIFPIFAILLFWGQFGIKTLKYRSGGMDEKTIALLVAGLM
Rhesus_macaque                   REGETIIELKYRV.........................VSWFSPNENILIVIFPIFAILLFWGQFGIKTLKYRSGGMDEKTIALLVAGLM
Green_monkey                     REGETIIELKYRV.........................VSWFSPNENILIVIFPIFAILLFWGQFGIKTLKYRSGGMDEKTIALLVAGLM
Sooty_mangabey                   REGETIIELKYRV.........................VSWFSPNENILIVIFPIFAILLFWGQFGIKTLKYRSGGMDEKTIALLVAGLM
Olive_baboon                     REGETIIELKYRV.........................VSWFSPNENILIVIFPIFAILLFWGQFGIKTLKYRSGGMDEKTIALLVAGLM
Crab-eating_macaque              REGETIIELKYRV.........................VSWFSPNENILIVIFPIFAILLFWGQFGIKTLKYRSGGMDEKTIALLVAGLM
Southern_pig-tailed_macaque      REGETIIELKYRV.........................VSWFSPNENILIVIFPIFAILLFWGQFGIKTLKYRSGGMDEKTIALLVAGLM
Drill                            REGETIIELKYRV.........................VSWFSPNENILIVIFPIFAILLFWGQFGIKTLKYRSGGMDEKTIALLVAGLM
Northern_white-cheeked_gibbon    REGETIIELKYRV.........................VSWFSPNENILIVIFPIFAILLFWGQFGIKTLKYRSGGMDEKTIALLVAGLM
Sumatran_orangutan               REGETIIELKYRV.........................VSWFSPNENILIVIFPIFAILLFWGQFGIKTLKYRSGGMDEKTIALLVAGLI
Western_gorilla                  REGETIIELKYRV.........................VSWFSPNENILIVIFPIFAILLFWGQFGIKTLKYRSGGMDEKTIALLVAGLM
Chimpanzee                       REGETIIELKYRV.........................VSWFSPNENILIVIFPIFAILLFWGQFGIKTLKYRSGGMDEKTIALLVAGLM
Bonobo                           REGETIIELKYRV.........................VSWFSPNENILIVIFPIFAILLFWGQFGIKTLKYRSGGMDEKTIALLVAGLM
African_elephant                 REGETTIQLKYRV.........................VSWFQKENILIIIFPILAILLFWGQFGVLILNSKSSRMNQETISVLVAGLF
Golden_hamster                   REGKTVIELKYRV.........................ASWFSPNEKILIVIFPILAILLFWGKFGILTLKYKSTRTNKRIILLLVAGLV
Mouse                            REGKTVIELKNRT.........................VSWFSPNEKILIVIFPILAILLFWGKFGILTLKYKSSHTNKRIILLLVAGLV
Rat                              REGKTVIELKNRP.........................VSWFSTNEKILIVIFPILAILLFWGKFGILTLKYKSSHTNKRIILLLVAGLA
Guinea_pig                       REGETVVEVKYRM.........................VSWFSPNENILIVIFPILAILLFWGQFGILTLKYKSNCTNMKIILSLIAGLL
Naked_mole_rat                   REGETIIEVKYRM.........................VSWFSPNENILIVIFPILAILLFWGQFGILTLKYKSNCTSTKIILLLIAGLL
American_beaver                  REGETVVELKYRV.........................VSWFSPNENILIVIFPILAILLFWGQFGILTLKYKSNRTNKKIIFLLIAGLF
Ords_kangaroo_rat                REGETVVELKYRV.........................VSWFSPNENILIVIFPILAILLFWGQFGLLTLKYKSNRTNRKIILLLIAGVV
Horse                            REGETIIELKYRV.........................VSWFSPKENILIVIFPILAILLSWGQFGIVTLKYKSSDTKKKIIFIFVVGLV
Western_European_hedgehog        REGETLIELKYRT.........................VPWFSPNENILTVVFAILAILLSWGQFGIVTLKYKSNQTKEKIILLFVAGLL
Cat                              REGETIIELKYRI.........................VSWFSPNENILIVIFPILAVLLSWGQFGIVTLKYKSSLMKEKTILLFVGGLV
Weddell_seal                     REGETVIELKYRI.........................VSWFSPNENILIVIFPILAVLLSWGQFGIVTIKYKASLVKEKTI.LFVGGLV
Dog                              REGETIIELKYRI.........................VSWFSPNENILIVIFPILAVLLSWGQFGIVTIKYKSSIMKEKTIFLFVGGLV
Red_fox                          REGETIIELKYRI.........................VSWFSPNENILIVIFPILAVLLSWGQFGIVTIKYKSSIMKEKTIFLFVGGLV
Hawaiian_monk_seal               REGETVIELKYRI.........................VSWFSPNENILIVIFPILAVLLSWGQFGIVTIKYKSSLVKEKTI.LFVGGLV
American_black_bear              REGETIIELKYRI.........................VSWFSPNENILIVIFPILAVLLSWGQFGIVTIKYKANLVKEKTILLLVGGLL
Grizzly_bear                     REGETIIELKYRI.........................VSWFSPNENILIVIFPILAVLLSWGQFGIVTIKYKANLVKEKAILLLVGGLL
Northern_fur_seal                REGETVIELKYRI.........................VSWFSPNENILIVIFPILAVLLSWGQFGIVTIKYKSSLVKEKTILLFVGGLV
Pacific_walrus                   REGETVIELKYRI.........................VSWFSPNENILIVIFPILAVLLSWGQFGIVTIKYKSNLVKEKTILLFVGGLV
Giant_panda                      REGETIIELKYRI.........................VSWFSPNENILIVIFPILAVLLSWGQFGIVTIKYKANLVKEKTILLLVGGLL
Polar_bear                       REGETIIELKYRI.........................VSWFSPNENILIVIFPILAVLLSWGQFGIVTIKYKANLVKEKTILLLVGGLL
Europen_domestic_ferret          REGETIIELKYRI.........................VSWFSPNENILIVIFPILAVLLSWGQFGIVTIKYKSSLVKEKTILLFVGGLV
Sea_otter                        REGETIIELKYRI.........................VSWFSPNENILIVIFPILAVLLSWGQFGIVTIKYKSSLVKEKTILLFVGGLV
Little_brown_bat                 REGETIVELKYRV.........................VSWFSPNENILIVIFPMLAVLLFWGQFGIVTLKYNASVTKEKTILLFVAGLV
Hybrid_cattle                    REGETIIELKYRV.........................VSWFSPNENILIVIFPVLAILLFWGQFGIVTLKYKSNYTKEKAIFLLVAGLL
Cow                              REGETIIELKYRV.........................VSWFSPNENILIVIFPVLAILLFWGQFGIVTLKYKSNYTKEKAIFLLVAGLL
Sheep                            REGETIIELKYRV.........................VSWFSPNENILIVIFPVLAILLFWGQFGIVTLKYKSNYTKEKAIFLLVAGLL
Goat                             REGETIIELKYRV.........................VSWFSPNENILIVIFPVLAILLFWGQFGIVTLKYKSNYTKEKAIFLLVAGLL
Pig                              REGETIIELKRRF.........................VSWFSPNENILIVIFPILAILLFWGQFGILTLKYKSSYTKEKTIFLLVAGLM
Atlantic_bottle-nosed_dolphin    REGETIVELKYRV.........................VSWFSPNENILTVIFPILAILLFWGQFGVVTLKYKSSYTKEKTIFLLVTGLV
Yangtze_finless_porpoise         REGETIVELKYRV.........................VSWFSPNENILTVIFPILAILLFWGQFGVVTLKYKSSYTKEKTIFLLVTGLV
Yangtze_river_dolphin            REGETIVELKYRV.........................VSWFSPNENILTVIFPILAILLFWGQFGVVTLKYKSSYTKEKTIFLLVTGLV
Beluga_whale                     REGETIVELKYRV.........................VSWFSPNENILTVIFPILAILLFWGQFGVVTLKYKSSYTKEKTIFLLVTGLV
North_pacific_minke_whale        REGETIVELKYRV.........................VSWFSPNENILTVIFPILAILLFWGQFGVVTLKYKSSYTKEKTIFLLVTGLV
Sperm_whale                      REGETIVELKYRV.........................VSWFSPDENILTVIFPILAILLFWGQFGVVTLKYKSSYTKEKTIFLLVTGLV
Duckbill                         REGEIIVELRYHV.........................VSWFNPKENILIVIFPILAILLSWGQFGIVTLKYKANCPSEKTFGLIISGII
Common                           REGETTVELRYHV.........................VSWFYPKENILIVIFPILAILLSWGQFGIVTLKYKSNCTSEKTIGLIIAGLA
Tasmanian                        REGETTVELKYHV.........................VSWFYPKENILIVIFPILAILLSWGQFGIVTLKYKSNCTSEKTIGLIIAGLA
Indian_muntjac                   REGETIIELKYRV.........................VSWFSPNENILIVIFPVLAILLFWGQFGIVS.....................
Reevess_muntjac                  REGETIIELKYRV.........................VSWFSPNENILIVIFPVLAILLFWGQFGIVS.....................
Domestic_yak                     REGETIIELKYRV.........................VSWFSPNENILIVIFPVLAILLFWGQFGIVTLKYKSNYTKEKAIFLLVAGLL
Spotted_hyena                    REGETIIELKYRI.........................VSWFSPNENILIVIFPVLAILLSWGQFGIVTHKYKSSLIKEKTILLFVGGLV
Iberian_lynx                     REGETIIELKYRI.........................VSWFSPNENILIVIFPILAVLLSWGQFGIVTLKYKSSLMKEKTILLFVGGLV
Meerkat                          REGETIIELKYRI.........................VSWFSPNENILIVIFPILAVLLSWGQFGIVTHKYKSSLMKEKTILLFVGGLV
Canada_lynx                      REGETIIELKYRI.........................VSWFSPNENILIVIFPILAVLLSWGQFGIVTLKYKSSLMKEKTILLFVGGLV
Greater_horseshoe_bat            REGDTVVELKYRL.........................VSWFSPNENILIVIFPMLAILLFWGQFGIVTLKLNSSIMKEKTILLFVAGLV
Groundhog                        REGETVIELKYRV.........................VSWFSPNENILVVIFPILAILLFWGQFGILTLKYKSNCTNKKIILLLIAGLL
West_Indian_manatee              REGETVIELKYRV.........................VSWFSPKENILIIIFPVLAILLSWGQFGVVTLKYASDPMKGKTILLFVAGLL
American_mink                    REGETIIELKYRI.........................VSWFSPNENILIVIFPILAVLLSWGQFGIVTIKYKSSLVKEKTILLFVGGLV
European_rabbit                  REGETTVELKYRV.........................VSWFSPNENILIVIFPILAILLFWGQFGILTLKYKSNRTNKKIILLLIAGLL
consensus>70                     REGeti!#lkyrv.........................vsWFspnEnILi!!Fpi.AiLL.WGqFGi.tlky.s....ekti.l.v.gl.
```

```
                                 170       180       190       200       210       220       230       240       250
CD47_HUMAN                       ITVIVIVGAILFVPGEYSLKNATGLGLIVTSTG.ILILLHYYVFST..AIGLTSFVIAILVIQVIAYILAVVGLSLCIAACIPMHGPLLI
Northern_greater_galago          VTIIIIVGAILFVPGEYSVKNATGLGLIVTSTG.LLIILNYYVFKT..AFGITSFVIALLIIQVLVYVLAGVGLSLCISACTPVHGPVLI
Coquerels_sifaka                 TTAVIIVGAILFVPGEYSVKNSTGLGLIVTTTG.LLLLLHYYVFKT..TIGIPSFVIAILVIQLLAYVLAVVGLSLCISACIPMHGPLLI
Common_marmoset                  ITVIVIVGAILFVPGEYSLKNATGLGLIVTSTG.ILILLHYYVFST..AFGLTSFVIAILAIQVIAYVLAVVGLSLCIAACIPMHGPLLI
Black-capped_squirrel_monkey     ITVIVIVGAILFVPGEYSLKNATGLGLIVTSTG.ILILLHYYVFST..AFGLTSFVIAILVIQVIAYVLAVVGLSLCIAACIPMHGPLLI
Panamanian_white-faced_capuchin  ITVIVIVGAILFVPGEYSLKNATGLGLIVTSTG.ILILLHYYVFST..AFGLTSFVIAILVIQVIAYVLAVVGLSLCIAACIPMHGPLLI
Nancy_Mas_night_monkey           ITVIVIVGAILFVPGEYSLKNATGLGLIVTSTG.ILILLHYYVFST..AFGLTSFVIAILVIQVIAYVLAVVGLSLCIAACIPMHGPLLI
Black_snub-nosed_monkey          ITVIVIVGAILFVPGEYSLKNATGLGLIVTSTG.ILILLHYYVFST..AIGLTSFVIAILVIQVIAYILAVVGLSLCIAACIPMHGPLLI
Golden_snub-nosed_monkey         ITVIVIVGAILFVPGEYSLKNATGLGLIVTSTG.ILILLHYYVFST..AIGLTSFVIAILVIQVIAYILAVVGLSLCIAACIPMHGPLLI
Angolan_Colobus                  ITVIVIVGAILFVPGEYSLKNATGLGLIVTSTG.ILILLHYYVFST..AIGLTSFVIAILVIQVIAYILAVVGLSLCIAACIPMHGPLLI
Rhesus_macaque                   ITVIVIVGAILFVPGEYSLKNATGLGLIVTSTG.ILILLHYYVFST..AIGLTSFVIAILVIQVIAYILAVVGLSLCIAACIPMHGPLLI
Green_monkey                     ITVIVIVGAILFVPGEYSLKNATGLGLIVTSTG.ILILLHYYVFST..AIGLTSFVIAILVIQVIAYILAVVGLSLCIAACIPMHGPLLI
Sooty_mangabey                   ITVIVIVGAILFVPGEYSLKNATGLGLIVTSTG.ILILLHYYVFST..AIGLTSFVIAILVIQVIAYILAVVGLSLCIAACIPMHGPLLI
Olive_baboon                     ITVIVIVGAILFVPGEYSLKNATGLGLIVTSTG.ILILLHYYVFST..AIGLTSFVIAILVIQVIAYILAVVGLSLCIAACIPMHGPLLI
Crab-eating_macaque              ITVIVIVGAILFVPGEYSLKNATGLGLIVTSTG.ILILLHYYVFST..AIGLTSFVIAILVIQVIAYILAVVGLSLCIAACIPMHGPLLI
Southern_pig-tailed_macaque      ITVIVIVGAILFVPGEYSLKNATGLGLIVTSTG.ILILLHYYVFST..AIGLTSFVIAILVIQVIAYILAVVGLSLCIAACIPMHGPLLI
Drill                            ITVIVIVGAILFVPGEYSLKNATGLGLIVTSTG.ILILLHYYVFST..AIGLTSFVIAILVIQVIAYILAVVGLSLCIAACIPMHGPLLI
Northern_white-cheeked_gibbon    ITVIVIVGAILFVPGEYSLKNATGLGLIVTSTG.ILILLHYYVFST..AIGLTSFVIAILVIQVIAYILAVVGLSLCIAACIPMHGPLLI
Sumatran_orangutan               ITVIVIVGAILFVPGEYSLKNATGLGLIVTSTG.ILILLHYYVFST..AIGLNSFVIAILVIQVIAYILAVVGLSLCIAACIPMHGPLLI
Western_gorilla                  ITVIVIVGAILFVPGEYSLKNATGLGLIVTSTG.ILILLHYYVFST..AIGLTSFVIAILVIQVIAYILAVVGLSLCIAACIPMHGPLLI
Chimpanzee                       VTVIVIVGAILFVPGEYSLKNATGLGLIVTSTG.ILILLHYYVFST..AIGLTSFVIAILVIQVIAYILAVVGLSLCIAACIPMHGPLLI
Bonobo                           VTVIVIVGAILFVPGEYSLKNATGLGLIVTSTG.ILILLHYYVFST..AIGLTSFVIAILVIQVIAYILAVVGLSLCIAACIPMHGPLLI
African_elephant                 LTMVVIVGAILFAPGEYSTKNASGLGLIVIPMV.ILQVLQYFVFKT..SFEMNYFAIAILILQVLGFVLAVVGFSLCVSECVPVQGPLLI
Golden_hamster                   LTVIVVVGAILLIPGENPVKSASGLGLIVISIG.TLILLQYNVFMT..AFGMTSFTIAILITQVLGYVLALVGLCLCIMACEPVHGPLLI
Mouse                            LTVIVVVGAILLIPGEKPVKNASGLGLIVISTG.ILILLQYNVFMT..AFGMTSFTIAILITQVLGYVLALVGLCLCIMACEPVHGPLLI
Rat                              LTLIVVVGAILFIPGEKPVKNASGLGLIVISTG.ILILLQYNVFMT..AFGMTSFTIAILITQVLGYVLAVVGMCLCIMACEPVHGPLLI
Guinea_pig                       LTIIVIVGAILFIPGEYSIKNSFGLGFIVISTG.VLILLQYNVFMT..AFGMTSFTIAILITQVLGYVLAVVGLCLCVTACVPADGPLLI
Naked_mole_rat                   LTIIVIVGAILFIPGEYSIKNSFGLGFIVISTG.ILILLQYNVFMT..AFGMTSFTIAILITQVLGYVLAVVGLCLCVTACVPVHGPLLI
American_beaver                  LTTVVIVGAIVFIPGEYSIENAAGLGLIVISTG.ILILLQYNVFMT..AFGMSSFTIAILITQVLGYVLAVVGLCLCIMACVPVHGPLLI
Ords_kangaroo_rat                LTAIVIVGAILFIPGEYSVKNASGLGLIVISTGIVLILLQYNVFMA..AFGMTSFTIAILITQVLGCVLAMVGLCLCMFACVPVHGPLLI
Horse                            LTIDVIVGAILFVPGEFSTKNACGLGLLVIPTA.ALILLQYCVFMI..SFRMTAFTIAILILQGLGFVLAVDGLSLCITECTPVHGPLLI
Western_European_hedgehog        LTAVVVVGAILFVPGEYSTKNASGLGLIVIPTV.ILILLQYCVFTI..AIGMSSFTIAILILQVLGYVLSLVGLSLCVSECISKNGPLLI
Cat                              LTIAVIVGAILFVPGEYSTKNSCGLGLIVVPTV.ILILLQYCVFMI..GVWMSSFTIAILILQVLGYVLSVVGLSLCVSECTPVNGPLLI
Weddell_seal                     LTIVVIVGAILFVPGEYSTKNSCGLGLIVIPTV.ILILLQYCVLII..GVRISPFTIAILILQVLGYVLSVVGLSLCVSECTPVHGPLLI
Dog                              LTIVVIVGAILFVPGEYSTKNSCGLGLIVIPTV.ILTLLQYCVFMI..GVWMSPFTIAILILQVLGYVLSVVGLSLCVSECTPVHGPLLI
Red_fox                          LTIVVIVGAILFVPGEYSTKNSCGLGLIVIPTV.ILTLLQYCVFMI..GVWMSPFTIAILILQVLGYVLSVVGLSLCVSECTPVHGPLLI
Hawaiian_monk_seal               LTIVVIVGAILFVPGEYSTKNSCGLGLIVIPTV.ILILLQYCVLII..GVRISPFTIAILILQVLGYVLSVVGLSLCVSECTPVHGPLLI
American_black_bear              LTIVVIVGAILFVPGEYSTKNSCGLGLIVIPTV.ILILLQYCVFII..GIWMSPFTIAILILQVLGYVLSVVGLSLCVSECTPVHGPLLI
Grizzly_bear                     LTIVVIVGAILFVPGEYSTKNSCGLGLIVIPTV.ILILLQYCVFII..GIWMSPFTIAILILQVLGYVLSVVGLSLCVSECTPVHGPLLI
Northern_fur_seal                LTIVVIVGAILFVPGEYSTKNSCGLGLIVIPTV.ILILLQYCMFVI..GGWISPFTIAILILQVLGYVLSVVGLSLCVSECTPVHGPLLI
Pacific_walrus                   LTIVVIVGAILFVPGEYSTKNSCGLGLIVIPTV.ILILLQYCMFVI..GGWISPFTIAILILQVLGYVLSVVGLSLCVSECTPVHGPLLI
Giant_panda                      LTIVVIVGAILFVPGEYSTKNSCGLGLIVIPTV.ILILLQYCVFII..GVWMSPFTIAILILQVLGYVLSVVGLSLCVSECTPVHGPLLI
Polar_bear                       LTIVVIVGAILFVPGEYSTKNSCGLGLIVIPTV.ILILLQYCVFII..GIWMSPFTIAILILQVLGYVLSVVGLSLCVSECTPVHGPLLI
Europen_domestic_ferret          LTIVVIVGAILFVPGEYSTKNSCGLGLIVIPTV.ILILLQYCVFII..GVWISPFTIAILILQVLGYVLSVVGLSLCVSECNPVHGPLLI
Sea_otter                        LTIVVIVGAILFVPGEYSTKNSCGLGLIVIPTV.ILILLQYCVFII..GVCMSPFTIAILILQVLGYVLSVVGLSLCVSECTPVHGPLLI
Little_brown_bat                 LTTFVIVGAILFVPGEYSTKRASGLGLIVVPTV.ILILLQYSVFMIAPAIGMSSFSITILILQVLGFVLSVVGLSLCVSECTPMHGPLLI
Hybrid_cattle                    LTVLVIVGAFLFIPGEYSTKNASGLGLIVLPTI.ILILLHYCVFMI..AMGMSSFTISILILQLLGYVLSVVGFSLCVSECIPVHGPLLI
Cow                              LTVLVIVGAFLFIPGGYSTKNASGLGLIVLPTI.ILILLHYCVFMI..AMGMSSFTISILILQLLGYVLSVVGFSLCVSECIPVHGPLLI
Sheep                            LTVLVIVGAFLFIPGEYSTKNASGLGLIVLPTV.ILILLHYCVFMI..AMGMSSYTISILILQILGYVLSVVGFSLCVSECIPVHGPLLI
Goat                             LTVLVIVGAFLFIPGEYSAKNASGLGLIVLPTV.ILILLHYCVFMI..AMGMSSYTISILILQILGYVLSVVGFSLCVSECIPVHGPLLI
Pig                              LTIIVIVGAILFIPGEYSTKNACGLGLIVIPTA.ILILLQYCVFMM..ALGMSSFTIAILILQVLGHVLSVVGLSLCVSECTPVHGPLLI
Atlantic_bottle-nosed_dolphin    LTIVVIVGAILFIPGEYSTKNACGLGLIVIPTA.ILILLQYCVFMI..AVGMSFFTIAILILQVLGHVLSVVGFSLCVSECTPVHGPLLI
Yangtze_finless_porpoise         LTIVVIVGAILFIPGEYSTKNACGLGLIVIPTA.ILILLQYCVFMI..AVGMSFFTIAILILQVLGHMLSVVGFSLCVSECTPVHGPLLI
Yangtze_river_dolphin            LTIVVIVGAILFIPGEYSTKNACGLGLIVIPTA.ILILLQYCVFMI..AVGMSFFTIAILILQVLGHVLSVVGFSLCVSECTPVHGPLLI
Beluga_whale                     LTIVVIVGAILFIPGEYSTKNACGLGLIVIPTA.ILILLQYCVFMI..AVGMSFFTIAILILQVLGHVLSVVGFSLCVSECTPVHGPLLI
North_pacific_minke_whale        LTIVVIVGAILFIPGEYSTKNACGLGLIVIPTA.ILILLQYYVFMI..TMGMSFFTIAILILQVLGHVLSVVGFSLCVSECTPVHGPLLI
Sperm_whale                      LTIVVIVGAILFIPGEYSTKNACGLGLIVIPTA.ILILLQYYVFMI..AVGMSFFTIAILILQVLGHVLSVVGFSLCVSECTPVHGPLLI
Duckbill                         LTLFVIIGAALFIPGEYSIKNASGLGLIVVPTL.MLVLLQYCVFMT..AFGMTSFAIAILILQLGGYILAAVGLSLCVMVCAPTQGPLLI
Common                           LTVMVIVGAVLFVPGEYSVKNASGLGLIVIPTV.TLVLLQYCVFMT..AFGMTLFAIGILILQLLGYVLAVIGLSLCITKCSPTQGPLMI
Tasmanian                        LTVVVIVGAVLFVPGEYSVKNAAGLGLIVIPTV.TLVLLQYCVFMT..AFGMTLFAIGILILQLLGYIFAVIGLSLCITKCSPMQGPLMI
Indian_muntjac                   ...............EYSTKNASGLGLIVLPTV.ILILLHYCVFMI..AMGMSSITISILILQILGYVLSVVGFSLCVSECVPVHGPLLI
Reevess_muntjac                  ...............EYSTKNASGLGLIVLPTV.ILILLHYCVFMI..AMGMSSITISILILQILGYVLSVVGFSLCVSECVPVHGPLLI
Domestic_yak                     LTVLVIVGAFLFIPGEYSTKNASGLGLIVLPTI.ILILLHYCVFMI..AMGMSSFTISILIIQLLGYVLSVVGFSLCVSECIPVHGPLLI
Spotted_hyena                    VTIAVIVGAILFIPGEYSTKNSCGLGLIVVPTV.ILILLQYCVFMI..GVWMSSFTIAILILQVLGYVLSVVGLSLCVSECTPVNGPLLI
Iberian_lynx                     LTIAVIVGAILFVPGEYSTKNSCGLGLIVVPTV.ILILLQYCVFMI..GVWMSSFTIAILILQVLGYVLSVVGLSLCVSECTPVNGPLLI
Meerkat                          LTIAVIVGAILFVPGEYSTKNSCGLGLIVVPTV.ILILLQYCVFMI..GVWMSSFTIAILILQVLGYVLSVVGLSLCVSECTPVNGPLLI
Canada_lynx                      LTIAVIVGAILFVPGEYSTKNSCGLGLIVVPTV.ILILLQYCVFMI..GVWMSSFTIAILILQVLGYVLSVVGLSLCVSECTPVNGPLLI
Greater_horseshoe_bat            LTIVVIVGAILFIPGEYSTKRASGLGLIVVPTV.VLILLQYAVFMIAPAIGMSSFSIAILILQVLGFVLSVVGLSLCVSVCAPVHGPLLI
Groundhog                        LTTIVIVGAILFVPGEYSVKNASGLGFIVISTG.VLILLQYNVFMT..AFGMTSFTIAILITQVLGYVLAVVGLCLCIMACIPVHGPLLI
West_Indian_manatee              LTIVVIVGAILFIPGEYSTKNASGLGLIVIPTV.ILILLQYFVLMT..AFGMTSFAIAILILQVLGFVLAVVGLSLCIPECTPVQGPLLI
American_mink                    LTIVVIVGAILFVPGEYSTKNSCGLGLIVIPTV.ILILLQYCVFII..GVWISPFTIAILILQVLGYVLSVVGLSLCVSECNPVHGPLLI
European_rabbit                  LTVIVIVGAILFVPGEYSIKNASGLGFIVISTG.ILISLQYNVFMT..AFGMTSFTVAILITQVLGYVLAVVGLCLCIMACVPVHGPLLI
consensus>70                     .tiivivgailfvpgeys.kna.GLGliV..t..iLilL.Y.vf.....gm..f.!aiLi.Qvl.yvl.vvGlsLCv..C.p.hGPl$I
```

```
                                   260       270                                                280       290        300
CD47_HUMAN                      SGLSILALAQLLGLVYMKFVA.SNQ.....................................K.TIQPPR..KAVEEPLNAFKESKGMMN
Northern_greater_galago         SGLGIFALVQLLGLIYMKFVG.SKK.....................................C.IDNPPN..EKI...............
Coquerels_sifaka                AGLVIFALIQLLGLVYMKFVA.SNQ.....................................K.TMNPPR..NN................
Common_marmoset                 SGLSILALAQLLGLVYMKFVE.FRT.....................................TPTVS.......................
Black-capped_squirrel_monkey    SGLSILALAQLLGLVYMKFVA.SNQ.....................................K.TIQPPR..KAVEEPLNAFKESKGMMN
Panamanian_white-faced_capuchin SGLSILALAQLLGLVYMKFVA.SNQ.....................................K.TIQPPR..KAVEEPLNAFKESKGMMN
Nancy_Mas_night_monkey          SGLSILALAQLLGLVYMKFVA.SNQ.....................................K.TIQPPR..KAVEEPLNAFKESKGMMN
Black_snub-nosed_monkey         SGLSILALAQLLGLVYMKFVA.SNQ.....................................K.TIQPPR..KAVEEPLNAFKESKGMMN
Golden_snub-nosed_monkey        SGLSILALAQLLGLVYMKFVA.SNQ.....................................K.TIQPPR..KAVEEPLNAFKESKGMMN
Angolan_Colobus                 SGLSILALAQLLGLVYMKFVA.SNQ.....................................K.TIQPPR..KAVEEPLNAFKESKGMMN
Rhesus_macaque                  SGLSILALAQLLGLVYMKFVE.FRGT....................................P.TV........................
Green_monkey                    SGLSILALAQLLGLVYMKFVA.SNQ.....................................K.TIQPPR..KAVEEPLNAFKESKGMMN
Sooty_mangabey                  SGLSILALAQLLGLVYMKFVA.SNQ.....................................K.TIQPPR..KAVEEPLN..........
Olive_baboon                    SGLSILALAQLLGLVYMKFVA.SNQ.....................................K.TIQPPR..KAVEEPLNAFKESKGMMN
Crab-eating_macaque             SGLSILALAQLLGLVYMKFVA.SNQ.....................................K.TIQPPR..KAVEEPLNAFKESKGMMN
Southern_pig-tailed_macaque     SGLSILALAQLLGLVYMKFVA.SNQ.....................................K.TIQPPR..KAVEEPLNAFKESKGMMN
Drill                           SGLSILALAQLLGLVYMKFVA.SNQ.....................................K.TIQPPR..KAVEEPLNAFKESKGMMN
Northern_white-cheeked_gibbon   SGLSILALAQLLGLVYMKFVA.SNQ.....................................K.TIQPPR..KAVEEPLNAFKESKGMMN
Sumatran_orangutan              SGLSILALAQLLGLVYMKFVA.SNQ.....................................K.TIQPPR..KAVEEPLNAFKESKGMMN
Western_gorilla                 SGLSILALAQLLGLVYMKFVA.SNQ.....................................K.TIQPPR..KAVEEPLNAFKESKGMMN
Chimpanzee                      SGLSILALAQLLGLVYMKFVA.SNQ.....................................K.TIQPPR..KAVEEPLNAFKESKGMMN
Bonobo                          SGLSILALAQLLGLVYMKFVA.SNQ.....................................K.TIQPPR..KAVEEPLNAFKESKGMMN
African_elephant                SGLGIIALAELLGLVYMKFVE.NKI.....................................K.TIVFSN..N.................
Golden_hamster                  SGLGIIALAELLGLVYMKFVA.SNQ.....................................R.TIQPPR..KAVEEPLNAFKESKGMMN
Mouse                           SGLGIIALAELLGLVYMKFVA.SNQ.....................................R.TIQPPR..N.................
Rat                             SGLGIIALAELLGLVYMKFVA.SNQ.....................................R.TIQPPR..N.................
Guinea_pig                      SGLGIVALAELLGLVYMKFVA.SNQ.....................................R.TIQPPR..KAVEEPLNAFKESKGMMN
Naked_mole_rat                  SGLGIIALAELLGLVYMKFVA.SNQ.....................................R.TIQPPR..KAVEEPLNAFKESKGMMN
American_beaver                 SGLGIIALAELLGLVYMKFVA.SNQ.....................................R.TIQPPR..KAVEEPLNAFKESKGMMN
Ords_kangaroo_rat               SGLGIIALAELLGLVYMKFVA.SNQ.....................................R.TIQPPR..KAVEEPLNAFKESKGMMN
Horse                           SGLGIIALAELFGLIYMKLAA.SNQ.....................................T.TIQPPR..KAVEEPLNAFKESKGMMN
Western_European_hedgehog       SGLGIIALAELLGLVYMKFVA.SNQ.....................................K.TIQPPR..KAVEEPLNAFKEPKGMMN
Cat                             SGLGIIALAELLGLVYMKLVA.SNQ.....................................K.TIQPPR..S.................
Weddell_seal                    SGLGIIALAELLGLVYMKLVG.KSV.....................................L.FLLASA..KNFKYIIWKIL.LIGSYN
Dog                             SGLGIIALAELLGLVYMKLVA.SNQ.....................................K.TIQPPR..KAVEEPLNAFKESKGMMN
Red_fox                         SGLGIIALAELLGLVYMKLVA.SNQ.....................................K.TIQPPR..KAVEEPLNAFKESKGMMN
Hawaiian_monk_seal              SGLGIIALAELLGLVYMKLVA.SSQ.....................................K.TIQPPR..KAVEEPLN..........
American_black_bear             SGLGIIALAELLGLVYMKLVA.SNQ.....................................K.TIQPPR..KAVEEPLNAFKESKGMMN
Grizzly_bear                    SGLGIIALAELLGLVYMKLVA.SNQ.....................................K.TIQPPR..KAVEEPLNAFKESKGMMN
Northern_fur_seal               SGLGIIALAELLGLVYMKLVA.SNQ.....................................K.TTQPPR..KAVEEPLNAFKESKGMMN
Pacific_walrus                  SGLGIIALAELLGLVYMKLVA.SNQ.....................................K.TIQPPR..KAVEEPLNE.........
Giant_panda                     SGLGITALAELLGLVYMKLVA.SNQ.....................................K.TIQPPR..KAVEEPLNAFKESKGMMN
Polar_bear                      SGLGIIALAELLGLVYMKLVA.SNQ.....................................K.TIQPPR..KAVEEPLNAFKESKGMMN
Europen_domestic_ferret         SGLGIIALAELLGLVYMKLVA.SNQ.....................................K.TIQPPR..KAVEEPLNAFKESKGMMN
Sea_otter                       SGLGITALAELLGLVYMKLVA.SNQ.....................................K.TIQPPR..KAVEEPLNAFKESKGMMN
Little_brown_bat                SGLGIIALAELLGLVYMKFVA.SNQ.....................................R.AIQPPR..NN................
Hybrid_cattle                   SGLGIIALAELLGLVYMKCVG.KSALFLLAYARNFKYIIWIMLLVDSYCLGPKKRMSNSKNEK.TQQMPL..Y.......FLN.......
Cow                             SGLGIIALAELLGLVYMKCVA.SNH.....................................R.TIQPPR..N.................
Sheep                           SGLGIIALAELLGLVYMKCVA.SNH.....................................R.TIQPPK..SN................
Goat                            SGLGIIALAELLGLVYMKCVA.SNH.....................................R.TIQPPK..KAVEEPLNAFKESKGMMN
Pig                             SGLGIIALAELLGLVYMKCVA.SDH.....................................K.TIQPPR..NN................
Atlantic_bottle-nosed_dolphin   SGLGILALAELLGLVYMKCVA.SNH.....................................K.TIQPPR..SN................
Yangtze_finless_porpoise        SGLGIIALAELLGLVYMKCVA.SNH.....................................K.TIQPPR..KAVEEPLN..........
Yangtze_river_dolphin           SGLGIIALAELLGLVYMKCVA.SNH.....................................K.TIQPPR..SN................
Beluga_whale                    SGLGIIALAELLGLVYMKCVA.SNH.....................................K.TIQPPR..KAVEEPLNAFKESKGMMN
North_pacific_minke_whale       SGLGIIALAELLGLVYMKCVA.SNH.....................................K.TIQPPR..KAVEEPLNAFKESKGMMN
Sperm_whale                     SGLGIIALAELLGLVYMKCFA.SNH.....................................K.TLQPPR..SN................
Duckbill                        SGLAIIALAELLGLVYMKFKA.SNQ.....................................K.PPQPPR..KAVEEPLNAFKESKGMMN
Common                          SGLGIIALAELLGLVYMKFVA.SNQ.....................................K.TIPPSR..KAVEEPLNAFKESKGMMN
Tasmanian                       SGLGIIALAELLGLVYMKFVA.SNQ.....................................K.TIPPSR..KAVEEPLNAFKESKGMMN
Indian_muntjac                  SGLGIIALAELLGLVYMKCVGNSNH.....................................K.T.........................
Reevess_muntjac                 SGLGIIALAELLGLVYMKCVG.NSK.....................................H.KTQDFCSRMACAH.SNGRQ.ITVIL.
Domestic_yak                    SGLGIIALAELLGLVYMKCVG.PKK.....................................R.MSNSKN.EKTQQMPLYFLN.......
Spotted_hyena                   SGLGIIALAELLGLVYMKLV..........................................................................
Iberian_lynx                    SGLGIIALAELLGLVYMKLFE.FRG.....................................TPSV........................
Meerkat                         SGLGIIALAELLGLVYMKLVA.SNQ.....................................K.TIQPPR..KAVEEPLNAFKESKGMMN
Canada_lynx                     SGLGIIALAELLGLVYMKLFA.SNQ.....................................K.TIQPPR..KAVEEPLNAFKESKGMMN
Greater_horseshoe_bat           SGLGIVALAELLGLVYMKFTA.TNQ.....................................R.TIQPPR..KAVEEPLNAFKESKGMMN
Groundhog                       SGLGIIALAELLGLVYMKFVA.SNQ.....................................R.TIQAPR..KAVEEPLNAFKESKGMMN
West_Indian_manatee             SGLGIIALAELLGLVSMKVVA.SSQ.....................................G.TIQPPR..KAVEEPLNAFKESKGMMN
American_mink                   SGLGIIALAELLGLVYMKLV..........................................................................
European_rabbit                 SGLGIIALAELLGLVYMKFVA.SNQ.....................................K.TIQPPR..KAVEEPLNAFKESKGMMN
consensus>70                    sGL.I.ALa#LlGL!yMK.va.sn........................................tiqppr..k.................
```

```
CD47_HUMAN                        DE................
Northern_greater_galago           ..................
Coquerels_sifaka                  ..................
Common_marmoset                   ..................
Black-capped_squirrel_monkey      DE................
Panamanian_white-faced_capuchin   DE................
Nancy_Mas_night_monkey            DE................
Black_snub-nosed_monkey           DGKYKLHLIIIDQLKHGH
Golden_snub-nosed_monkey          DE................
Angolan_Colobus                   DE................
Rhesus_macaque                    .S................
Green_monkey                      DE................
Sooty_mangabey                    .E................
Olive_baboon                      DE................
Crab-eating_macaque               DE................
Southern_pig-tailed_macaque       DE................
Drill                             DE................
Northern_white-cheeked_gibbon     DE................
Sumatran_orangutan                DE................
Western_gorilla                   DE................
Chimpanzee                        DE................
Bonobo                            DE................
African_elephant                  .K................
Golden_hamster                    DE................
Mouse                             .R................
Rat                               .N................
Guinea_pig                        EE................
Naked_mole_rat                    EE................
American_beaver                   DE................
Ords_kangaroo_rat                 DE................
Horse                             DAARCSRCVCLSN.....
Western_European_hedgehog         DE................
Cat                               .N................
Weddell_seal                      L.................
Dog                               DE................
Red_fox                           DE................
Hawaiian_monk_seal                E.................
American_black_bear               DE................
Grizzly_bear                      DE................
Northern_fur_seal                 DE................
Pacific_walrus                    ..................
Giant_panda                       DG................
Polar_bear                        DE................
Europen_domestic_ferret           DE................
Sea_otter                         DE................
Little_brown_bat                  ..................
Hybrid_cattle                     .N................
Cow                               .N................
Sheep                             ..................
Goat                              DE................
Pig                               ..................
Atlantic_bottle-nosed_dolphin     ..................
Yangtze_finless_porpoise          .........E........
Yangtze_river_dolphin             ..................
Beluga_whale                      DGKYKMHLQL........
North_pacific_minke_whale         DE................
Sperm_whale                       ..................
Duckbill                          DG................
Common                            DE................
Tasmanian                         DG................
Indian_muntjac                    ..................
Reevess_muntjac                   ..................
Domestic_yak                      .N................
Spotted_hyena                     ..................
Iberian_lynx                      .S................
Meerkat                           DE................
Canada_lynx                       DE................
Greater_horseshoe_bat             DE................
Groundhog                         DE................
West_Indian_manatee               DGKYTMYLQ...F.....
American_mink                     ..................
European_rabbit                   DE................
consensus>70                      ..................
```
