# Supplementary material for: Structure of the human marker of self 5-transmembrane receptor CD47
Source: Nat Commun. 2021 Sep 1;12:5218. doi: 10.1038/s41467-021-25475-w (PMC8410850; doi:10.1038/s41467-021-25475-w)
Supplement: Supplementary file 5 — Supplementary Data 2 [file 41467_2021_25475_MOESM5_ESM.pdf]

```
CD47_HUMAN                       ..........................................................................................
American_chameleon               MWVLR........VCWVL....LGTL................................................................
King_cobra                       MWMLC........VWWVL....LGTL................................................................
Chinese_softshell_turtle         LFNIS........V.AFF....LRRC................................................................
Agassizs_desert_tortoise         MWALG........A.WVL....LGAV................................................................
Chicken                          MWLLT........V.GAL....LAVL................................................................
Northern_mallard                 MSLPAPCPPLLLLL.LLL....LGAL................................................................
American_alligator               MWAPG........L.WVL....LGAV................................................................
Band_tailed_pigeon               MWLLA........A.WVL....LSAV................................................................
Rock_dove                        ..........................................................................................
Collared_flycatcher              MCQFS........F...........F................................................................
Bengalese_finch                  MWLLA........A.SVL....LAAL................................................................
Zebra_finch                      MWLLA........A.SVL....LAAL................................................................
Burrowing_owl                    MCQFS........F.F..........................................................................
Bar-tailed_godwit                MVRGC........S.SIPPPPPPPPG................................................................
Golden_eagle                     MWPLA........A.WVL....LSAV................................................................
Kakapo                           MWLLP........A.WVL....LSAV................................................................
Northern_Bobwhite                MWLLT........A.GAL....LAVL................................................................
Barn_swallow                     MWLLA........A.SVL....LGAL................................................................
Ring-necked_pheasant             MWLLT........A.GTF....FAVL................................................................
Common_box_turtle                MWALG........T.WVL....LGAV................................................................
Big-headed_turtle                MWALG........T.WVL....LGAV................................................................
Eastern_brown_snake              MWMLC........VWWVL....LGTL................................................................
Eastern_diamondback_rattlesnake  MWMLC........VWWVL....LGTL................................................................
South_American_coral_snake       MWMLC........VWWVL....LGTL................................................................
Brown_tree_snake                 MWMLC........VWWVL....LGTL................................................................
MacQueens_bustard                ..........................................................................................
Cuckoo_roller                    ..........................................................................................
Hoatzin                          ..........................................................................................
Adelie_penguin                   ..........................................................................................
Speckled_mousebird               ..........................................................................................
Golden-collared_manakin          ..........................................................................................
White-throated_tinamou           ..........................................................................................
Red-throated_loon                ..........................................................................................
Red-crested_turaco               ..........................................................................................
Great_crested_grebe              ..........................................................................................
Northern_carmine_bee-eater       ..........................................................................................
Northern_fulmar                  ..........................................................................................
Emperor_penguin                  ..........................................................................................
White-tailed_eagle               ..........................................................................................
Dalmatian_pelican                ..........................................................................................
Grey_crowned_crane               ..........................................................................................
Common_cuckoo                    ..........................................................................................
Sunbittern                       ..........................................................................................
American_crow                    ..........................................................................................
Brown_mesite                     ..........................................................................................
Barn_owl                         ..........................................................................................
American_flamingo                ..........................................................................................
Annas_hummingbird                ..........................................................................................
Red-legged_seriema               ..........................................................................................
Gouldian_finch                   MWHLQ........D.DNL....RDTWRRGAHPVIHCLQELLGGWWWKEHLQEALGLPLVGEQPRNLSRETRWVEGHRTRKNVNVLALKKE
Little_egret                     ..........................................................................................
Yellow-throated_sandgrouse       ..........................................................................................
Turkey_vulture                   ..........................................................................................
Downy_woodpecker                 ..........................................................................................
Kea                              ..........................................................................................
Killdeer                         ..........................................................................................
Scaled_quail                     MWLLT........A.GAL....LAVL................................................................
Ocelot_gecko                     LNNPC........V.WFA....SCLT................................................................
Common_wall_lizard               MWALS........VCWVL....LGTL................................................................
consensus>70                     ..........................................................................................
```

```
                                           1         10        20        30        40        50        60        70
CD47_HUMAN                          .......QLLFNKTKSVEFTFCNDT.VVIPCFVTNMEAQNTTEVYVKWKFKGRDIYTFDGALNKS..TVPTDFSSAKI.EVSQLLKGDAS
American_chameleon                  ..AAGSAQLYFEKVRSVTLDICNTS.AIIPCRVTNLILHRTNAIFVKWKLQGSEFFSYDGVENKV..TRNSTFQSANLLDLSLLPNGTAS
King_cobra                          ..GAGSAQLSFQNVPFLEINYCNGPQFVLPCVVTNLKENNSVSMFVSWQFQGNPFFIYDGVSDNI..TKNNSFHTAQFLNRSMLPYGIVS
Chinese_softshell_turtle            ..CISSSQLMFNATKSVESSICNTT.VMLPCIVNNLELKRTERMFIKWKLGQELVFAYEGIKRYS..HRTKDFPSAMLAFEDRLPQGIAS
Agassizs_desert_tortoise            ..CAGSAQLQFNVIKSVEQTICNQT.VILPCIVTNLAQKNTKVMFVKWKLEKKEFFSFSGETEMS..HTNENFSSAKLESLQNLKLGVAS
Chicken                             ..GAGSTQLVFNAVDFVEKYACNDT.VVLPCIVTNLKENNDSSMHVSWKRQGQVIFSFNGPEQRI..YRHESVPSANFLSKADLFKGIAS
Northern_mallard                    ..RHGSAQLVFSPVDFVERDACNTT.VVLPCHVVNLKEHNDSNMFVAWKKQGIVLFSFDGAQQRV..YRHETVPSANLLSQQDLSKGTAS
American_alligator                  ..GTGSAQLLFNATKSVNLSTCNES.AILPCIVNNLAFHSAGDMFVKWKFNGTEFFAFNGFSKKL..RRSDKFSSANFTSEKDLPKGIAS
Band_tailed_pigeon                  ..GAGSAQLTFSGPDVVERTDCNRT.VTLPCYVNNLKENNIKVMFVTWKKQGKIIFSFDGAKQAF..LRDETVPSANLTSQEDLPKGIAS
Rock_dove                           .......AQLTFSGPDVVERTDCNRT.VTLPCYVNNLKENNIKVMFVTWKKQGKKIFSFDGAKQEF..LRDETVPSANLTSQEDLPKGIAS
Collared_flycatcher                 ..FIGSAQLILIGTSVIEKSGCNET.VVLPCHVTNLRQKNENAMFVIWKKEGDTIFSYRGGNKKY..NVDNSFSSAKFLHPENLTDGDAS
Bengalese_finch                     ..RAGSAQLSLYGTSVVEKNDCNKT.VVLPCYVTDLKQNNENVMSVTWKRQGVTIFFYHGGNKKF..NIDPSFSSARFLSKAELIKGVAS
Zebra_finch                         ..RAGSAQLSLYGTSVVEKNDCNKT.VVLPCYVTDLKQNNENVMSVTWKRQGVTIFFYHGGNKNF..NIDPSFSSARFLSKAELIKGVAS
Burrowing_owl                       ..FLGSAQLMFSVTDVVEKTECNKT.VILPCHVINLKGNNANVMFVTWKKQGKRIFSFDGARQQF..YRDPTVPSANLASLADLPKGIAS
Bar-tailed_godwit                   ..SGGSAQLLFNATDVVKKTDCNET.VILPCYVINLNENNPNIMFVTWKREGKIIFSFDGAGKEF..FRDPTVPSANLLSEADLPKGVAS
Golden_eagle                        ..GAGSAQLIFSVTDVVEKTDCNKT.VVLPCYVTNLKENNANVMFVTWKKQGKVIFSFDGARQEF..FRDPAVPSANLVSRADLPKGIAS
Kakapo                              ..GAGSAQLIFSGTDVVERTDCNKT.VILPCYVTNLKDNNPNVTFVTWKKQRKIIFSFDGKNQEF..FRDPEFQSADLVSRADLPKGIAS
Northern_Bobwhite                   ..GAGSTQLVFNVVDLVEKNACNET.VILPCIVTNLKENNDSSMHVSWKRQGQVLFSFNGPEQRI..YRHESVPSANFLSKSDLSKGIAS
Barn_swallow                        ..RAGSAELTLEGGGIIEKNDCNET.VVLPCYVTNLEEKNENALFVTWKKQETPIFSYRGATKKF..TIDPSFSSARFLSQGKLIDGDAS
Ring-necked_pheasant                ..GAGSTQLVFNAVDLVEKYACNDT.VVLPCIVINLNDNNDSSMHVSWKRQGQVIFSFNGPEQHI..YRHDSVPSANFLSKMDLSKGIAS
Common_box_turtle                   ..CAGSAQLQFNVTKSVEQTACNKT.VILPCIVTNLIKNNIQVIFVKWKLEKKAFFIFEGSTGKI..NRNESFSSAALESPQNLTSGIAS
Big-headed_turtle                   ..CAGSAQLQFNVIKSVEQTICNKT.VTLPCIVTNLAQNNTKVMFVKWKLEKKEFFSFDGSNGKF..DRNVNFSSAMLGSLQNLKLGIAS
Eastern_brown_snake                 ..GAGSAQLSFQNVPFLEINYCNGPQFVLPCVVTNLEENNSVTMFVSWKFQGNPLFTYDGVSGNI..TKGNNFETAKFLNHSMLPYGIAS
Eastern_diamondback_rattlesnake     ..GAGSAQLVLDSIPFLEIKHCNGGEIILPCVVTNLKEHSSLSMFVKWHFQGNQFFLYDGVTDNI..TKNNSFDTATFLNRSMVPYGIVS
South_American_coral_snake          ..GAGSAQLSFQNVPFLEINYCNGPQFVLPCVVTNLKENNSLSMFVSWQFQGNPFFSYDGVSDNI..TKNNRFDSAKFLNRSMFPYGIAS
Brown_tree_snake                    ..GAGSAQLSFAKVPFLEMNNCNGPKIILPCVVTNLKENSSMAMFVSWQFQGNQFFIYDGVSDNV..TKNNSFNTATFLNRSMVPYGIVS
MacQueens_bustard                   ....GSAQLTFNVTDPVEINNCNKT.VLLPCYVNNLRENNVHVMFIRWRKGREKVLSFDGENEKT..V..VKVPSARLPPVADILKGNAS
Cuckoo_roller                       ....GSAQLIFSGTDVVERTDCNKT.VILPCYVTNLKENNANVMFVRWKKEGKIIFSFDGARQEF..FRDPTVPSANLVSQADLPKGFAS
Hoatzin                             ....GSAQLVFSVTEVVERTVCNKT.VTLPCHVTNLKENNTYAMFVTWKKQGKIIFSFDGATQAF..FKDPMVPSADLVSQADLPKGIAS
Adelie_penguin                      ....GSAQLIFSVTDVVERTDCNKT.VILPCYVTNLKEDNADVMFVTWKKQGKIIFSFDGAKRQF..FRDAAVPSANLVSQADLPKGIAS
Speckled_mousebird                  ....GSARLYFHTISFVERTACNKT.VILPCYVSNLQLFSVKYIFVTWKKEGNRIFAFDGSKNEF..FRHSAYQSASLLSDEELAKGIAS
Golden-collared_manakin             ....GSAKLNLTGTDVVEKNDCNKT.VILPCYVTNLMKNNETLMFVKWKKEGKVIFSFHGGRNEF..YRDGDFSSAKLLSRADLTKGVAS
White-throated_tinamou              ....GSAQLLFNVTSFVEKNICNET.ILLPCLVYNLQANDKTVMYVKWKRQGNLFFSFNGEHQS...VTDSNFSSARLLSESDLSKGVAS
Red-throated_loon                   ....GSAQLIFSATDVVERSDCNKT.VILPCYVTNLKENNANVMFVTWKKQGKIIFSFDGAKEEF..FNDPMFLSAGLVSHADLPKGIAS
Red-crested_turaco                  ....GSAQLKFVVTDVVERTDCNKT.VILPCIVTNLKDNNPEVMFVRWKRQGKIIFSFDGPNQKF..LRHPTVPSANFVSQADLPKGVAS
Great_crested_grebe                 ....GSAQLTFNVTDVVERTDCNKT.VVLPCYVTNLKENNVNIMFVAWKKQGKVIFSFDGAKGEF..FRDPSVPSANL.SQADLPKGVAS
Northern_carmine_bee-eater          ....GSAQLIFTVTDVVEMTACNRT.VLLPCNVTNLQEKNSKVMFVTWKKQGKVIFSFDGARQET..FIDPTVPSAKLASEMDLPNGNAS
Northern_fulmar                     ....GSARLILSATDVVERTDCNKT.VILPCYVTNLKENNANVMFVTWKKQGKIIFSFDGARQEF..FRDPAVPSANLVSQADLPKGIAS
Emperor_penguin                     ....GSAQLIFSVTDVVERTDCNKT.VILPCYVTNLKEDNANVMFVTWRKQGKIIFSFDGAKQQF..FRDPAVPSANLVSQADLPKGIAS
White-tailed_eagle                  ....GSAQLIFSVTDVVEKTDCNKT.VVLPCYVTNLKENDANVMFVTWKKQGKVIFSFDGARQEF..FRDPAVPSANLLSRADLPKGIAS
Dalmatian_pelican                   ....GSAQLIFRVTDFVGKSDCNKT.VILPCYVTNLKENDVSVMFVTWKKQGKGFFSFDGARQEF..FRDQAFQSANLVSQADLPKGDAS
Grey_crowned_crane                  ....GSAQLIFSGTNVVEKTDCNKT.VILPCHVTNLKENNINVMFVTWKKQGNRIFSFNGANQKF..LRDPTVPSANLVSQADLPKGVAS
Common_cuckoo                       ....GSAQLELTTRSPVESTSCNRT.VTLPCYVVDLKLTSAKGMFVTWQKHDKLIFSYDGATQEF..FRDPVVPSANLASLVDLPKGVAS
Sunbittern                          ....GSAQLLFNATDVVERTVCNQT.VVLPCNVINLKENSTKVMFVTWKKQGKIIFSFDGAKQDF..FRDPAFSSANLVSQADLPKGIAS
American_crow                       ....GSAQLSLVGTGVIEKNDCNET.VVLPCYVTNLKENNENLMLVTWKKQGDIIFSYHGGKKEF..NINPSFSSARLLSQPDLIKGVAS
Brown_mesite                        ....GSAQLIFNVTDVVERTICNKT.VILPCYVTNLKENNASVMFVTWKKQGKRIFSFDGGKVEF..FKDPAFSSANLVSQADLPKGVAS
Barn_owl                            ....GSAQLMFSVTDVVEKTDCNKT.VILPCYVTNLKENNANAMFVMWKKQGKIIFSFDGARQGF..FRDPAVPSANLVSLADLPKGTAS
American_flamingo                   ....GSAQLTFSAIDVVERTVCNKT.VVLPCYVTNLKENNASVMFVMWKKQGETIFSFDGGRREF..FRHQEFPSANFVSQVDLPRGVAS
Annas_hummingbird                   ....GSAQLLFNVTDFVEITQCNKT.VILPCYVTNLKENSTKVMFVNWRKEDKTIFSFDGEREEF..LRDPSVPSANLVSQANLPKGTAS
Red-legged_seriema                  ....GSAQLAFNVTDVVERTDCNKT.VILPCYVTNLHEKNASVMFVTWKKQGKIIFSFGGARQEY..FRDPAVPSANLVSLADLPKGVAS
Gouldian_finch                      KNPHSSAQLSLSGTSVVEKNDCNKT.VVLPCYVTDLKQNNENVMSVTWKKQGVKIFFYHGGNKKF..NIEPSFSSARFLSHPDLIKGVAS
Little_egret                        ....GSAQLMFSATDVLEVTKCNKT.VILPCYVTNLKENNSSVMFVTWKKQRKTIFSFDGEKQQF..VRDPAVPSANLLSQKDLPKGVAS
Yellow-throated_sandgrouse          ....GSAQLIFSVTDVVEKTDCNKT.VTLPCYVTNLKENNVEAMFVTWKKEGKIIFSFDGANRQF..YRDPTVPSADLVSQEDLPKGIAS
Turkey_vulture                      ....GSAQLIFSVTDVVERTDCNKT.VILPCYVTNLKENNANVMFVTWKKQGKIIFSFDGARREF..FRDPAVPSANLVSQADLPKGFAS
Downy_woodpecker                    ....GSAQLALTTTD.VETTNCNKT.VILPCYVTDLHENNEKTMIVLWALNEREIFSYEGANRTV..FRDNAFPSANLLSPGDLLKGVAS
Kea                                 ....GSAQLILSGTDVVERTDCNKT.VILPCYVTNLKENNANVMFVTWKKQRKIIFSFDGKKQEV..FRDPEFQSADLLSRADLPKGVAS
Killdeer                            ....GSAQLMFNVTDVVEKTDCNKT.VILPCHVINLKENNADIMFVTWKRQGKRIFSFDGAGRKS..LRDPAVPSANLVSEADLPKGVAS
Scaled_quail                        ..GAGSTQLVFNVVDLVEKNACNET.VILPCIVTNLKENNDSSMHVSWKRQGQVLFSFNGPEQRI..YRHESVPSANFLSKSDLSKGIAS
Ocelot_gecko                        ..PTGSAQLVTEKFTSVLMDNCNAS.VILPCIIRNLDKNNTRAMYVKWKLGGNEFFSFDGYATPPIHKKNSTFQSAKFVSLPKLTAGIAS
Common_wall_lizard                  ..GAGSAQLMFKKIHNVSFDVCNTTEIIIPCIVTNLQRFSREEMFLNWKIDGKEFFTYDGYENRY..FKNNTFLSVDLLDILNLTAGVAS
consensus>70                        ....gsaqL.f.....ve...CN.t.vilPC.!t#$.enn...mfv.Wk.qg..ifs%dG........d....sa...s..dl.kGiaS
```

```
                                    80        90        100       110                                              120
CD47_HUMAN                          LKMDKSDAVSHTGNYTCEVTELTREGETIIELKY..............R.............V................VSWFSPNENIL
American_chameleon                  LILSREEA..PPGNYTCDVAESSREGDTIVELIHI...........................S................TSWFQPTENSF
King_cobra                          LVISTNET..VLGNYTCEVTEVNREGKTRFELRAS...........................S................VPWFQPVERFS
Chinese_softshell_turtle            LKLSRTEA..PAGNYTCEVTESNREGETIIELTYR...........................Q................ESWFTSLENIL
Agassizs_desert_tortoise            LTISKTQA..VPGNYSCEVTESNREGETVIELVYR...........................I................VSWFTPIENSL
Chicken                             LRLKNAEA..PDGNYSCEVTELNREGETKMKLRT..............H.............MVDSCDEEKPPTPVDKCERQFDFIQSIV
Northern_mallard                    LKLKSSEA..EDGNYSCEVTELNREGETKVDLRT..............V.............TVDNCGEEKAPTPDNKCGRQFDFMESVV
American_alligator                  LSILAAQA..LPGNYTCEVTESNREGEIVVELKISVGNESDCRDRLPCP.............S................GSWFPPVESVF
Band_tailed_pigeon                  LTLISAEA..GVGNYSCEVTESNREGETRVELRH..............S.............S................GPWFLLVESAI
Rock_dove                           LTLISAEA..SVGNYSCEVTESNREGETRVELRH..............S.............S................GPWFLLVESAI
Collared_flycatcher                 LVLSSKQA..VVGNYSCEVTESNREGELRMELRN..............GSVASCGDETPEEKC................GSWFLLVERAV
Bengalese_finch                     LVIDSVQA..TVGNYSCKVTESNSEGEIKMELKN..............G.............S................GSWFLLMERVG
Zebra_finch                         LVIDSAQA..TVGNYSCKVTESNSEGEIKMELKS..............S.............S................GSWFLLMERVG
Burrowing_owl                       LMLDGAEA..AVGNYSCEVTELNREGETTVELRQ..............R.............I................GSWFLLVEKAI
Bar-tailed_godwit                   LRLNNAEA..EVGNYSCEVTESNREGETKLELKK..............Y.........................................
Golden_eagle                        LMLNSAEA..DVGNYSCEVTESNREGETRVELRK..............H.............SVVSCGEENTPTPKEKCGSWFLLVERAI
Kakapo                              LMLSGAEA..TVGNYSCEVTESNREGETRIELRN..............H.............S................GSWFLLMERAI
Northern_Bobwhite                   LRLKNAEA..TDGNYSCEVTELNREGETKFELRT..............R.............M................ERQFDLVQSIV
Barn_swallow                        LVLNSAQA..TVGNYSCKVTESNREGEIKMELKS..............S.............S................GSWFLLVERAV
Ring-necked_pheasant                LRLKNAEA..TDGNYSCEVTELNREGETKMELRT..............H.............MVDSCDEEKPPTPVDKCERQFDLIQSIV
Common_box_turtle                   LTISKTQA..VRGNYSCEVTESSREGETVIELIYH...........................R................VPWFTPVENIL
Big-headed_turtle                   LTISKTQA..VPGNYSCEVAESNREGETVIELIYR...........................I................VSWFTPVENIL
Eastern_brown_snake                 LVISTNQA..VLGNYTCEVTEGNREGKTRFELRAS...........................SACL..NY.....SSESVPWFQPVERFS
Eastern_diamondback_rattlesnake     LVISTKQA..LLGNYTCEVTEVNREGRTKFELRES...........................SAC..........PSECVPWFQPVERFS
South_American_coral_snake          LVILTNEA..VLGNYTCEVTEGNREGKTRFELRTN...........................SACL..NN.....SSESEPWFQPVERFS
Brown_tree_snake                    LSMSTKQA..LLGNYTCEVTEGNREGKTRVELRES...........................S................VPWFNPVERFS
MacQueens_bustard                   LTLNSAEA..DIANYSCEVTESNREGAVTVEIRN..............R.............S................GSWFLLVERAI
Cuckoo_roller                       LMLNGAEA..DAGNYTCEVTESNREGQTRFELRN..............I.............S................GSWFLLVERAI
Hoatzin                             LKLNSAEA..EDGNYSCKVTESSREGETRVELRN..............R.............S................GSWFLLLHRAI
Adelie_penguin                      LMLKSAEA..DAGNYSCEVTESNREGETRVELRN..............H.............S................GSWFLLVERAI
Speckled_mousebird                  LKLKGTEA..GVGNYSCEVTESNREGETTVELRS..............R.............P................GSWFLLMERAI
Golden-collared_manakin             LVLTTRQA..TVGNYSCEVTESNTEGDTKMELRQ..............S.............S................GSWFLLVERAI
White-throated_tinamou              ILLNKKEA..ILGNYSCEVTESNREGETTVELRN..............S.............S................GSWFLPVESAC
Red-throated_loon                   LKLNSAEA..DVGNYSCEVTESNREGETRVELRN..............H.............S................GSWFLLVERAI
Red-crested_turaco                  LMLNGAEA..EVGNYSCEVTESNTEGESRVELIN..............R.............S................GSWFLLVEKAV
Great_crested_grebe                 LTLNSAEA..SAGNYSCEVTESNSEGETRVELRN..............R.............S................GSWFLLLEMAI
Northern_carmine_bee-eater          LILNSAQA..DVGNYSCEVTESNREGETRVELRN..............R.............S................GSWFLLVERAI
Northern_fulmar                     LTLNSAEA..DVGNYSCEVTESIRDGETRIELRN..............H.............S................GSWFLLVERAI
Emperor_penguin                     LMLKSAEA..DAGNYSCEVTESNREGETRVELRN..............H.............S................GSWFLLVERAI
White-tailed_eagle                  LMLNSAEA..DVGNYSCEVTESNREGETRFELRK..............H.............S................GSWFLLVERAI
Dalmatian_pelican                   LMLDSAEA..DFGNYSCEVTESNREGETTVELKN..............L.............S................GSWFLLVESVV
Grey_crowned_crane                  LMLNSAEA..DVGNYSCEVTESNREGETRVELRN..............G.............S................GSWFLLAERAV
Common_cuckoo                       LTFKNGEG..VDGNYSCKVTVSNSEGKITVELRS..............I.............T................GPWFVLMERAF
Sunbittern                          LMLNTSEA..EVGNYSCEVTESNREGETKVELRD..............H.............S................GSWFMLVDTAI
American_crow                       LVLSSAQA..TVGNYSCEVIESNSVGEIKMELRN..............S.............S................GSWFLLVERAV
Brown_mesite                        LTLNSAEA..DAGNYSCEVTESSREGETRVELRN..............H.............T................GSWFVLVERAI
Barn_owl                            LMLDSAEA..DVGNYSCEVTESSREGETRVELRH..............R.............L................GSWFLLVERAI
American_flamingo                   LMLNGGEA..GAGNYSCEVTESNREGETRVELRN..............R.............S................GSWFLQVEMAI
Annas_hummingbird                   LKLSSAEA..KPGNYSCQVTELNREGRTTVELRN..............I.............S................GSWFLLVERAI
Red-legged_seriema                  LMLNSAEA..DVGNYSCEVTESNREGETRVELRN..............H.............S................GSWFLLLERAI
Gouldian_finch                      LVLDSAQA..TVGNYSCKVTESNSEGEIKMELKS..............SSVVSCGDGTPEDKC................GSWFLLMERVG
Little_egret                        LILNSAEA..DAGNYSCEVTESNREGETTVELRK..............P.............S................GSWFLLMERAI
Yellow-throated_sandgrouse          LTLDSTEA..DVGNYSCEVTESNREGETRVELRN..............L.............S................GSWFLLMERAI
Turkey_vulture                      LMLNSAEA..DVGNYSCEVTESNREGETRVELRN..............Q.............S................GSWFLLVERAI
Downy_woodpecker                    LTLSSAEA..EVGNYTCQVTESNREGKIRVELKS..............R.............V................GSWFPLVERAI
Kea                                 LVLNGAEA..AVGNYSCEVLELSREGETRIELRN..............H.............S................GSWFLLMERAI
Killdeer                            LRLNSAEA..VVGNYSCEVTESNREGETRVELRK..............Y.............S................GSWFLLVERAI
Scaled_quail                        LRLKNAEA..TDGNYSCEVTELNREGETKFELRT..............R.............M................ERQFDLVQSIV
Ocelot_gecko                        LSLSRKEA..ILGNYSCEVTESNREGMATIELKER...........................L................GTWFQPVENVL
Common_wall_lizard                  IKMSLKEA..VPGNYTCIVVERNREGEHVIELRYG...........................T................PFWFQPVESFL
consensus>70                        l.l...#a....gNYsCeVtesnreGet.velr..............................................gswf..ve...
```

```
                                  130         140         150         160         170         180         190         200         210
CD47_HUMAN                        IVIFPIFAILLFWGQFGIKTLKYRSGGMDEKTIALLVAGLVIT.VIVIVGAILFVPGEYSLKNATGLGLIVTSTGILILLHYYVFSTAI.
American_chameleon                IIATVIVAAVLYFSQSVVVAMRFDMT..LSKKIGLFLAELIVI.FLAVIGVILLIPAGYESSQQIGLALIVVPAFILVPILHFLFTSVFE
King_cobra                        IISIIVLIVILYWCQFITVAQKFDMT..FKKKIGFTVTGLLIS.IFAVIGTILYAQGEYTTSVKTGIGLIVIPAIIVVPLLYFLFTSVFE
Chinese_softshell_turtle          IIFFLVLAILSYWIHLGITVSKFETA..FWKKRLLFIGGVVIT.VLALIGFILLVPGGFGPKNQAGLSLIVLPSVILVPLQYFLLQIVSG
Agassizs_desert_tortoise          IILFMVLAILFYWIQLAITVSKFETT..L.LKISLFIAGVVIS.VFAVVGFSLLISDEFKPENQIGLGLIVLPAVILVPSQYFLLQKVCE
Chicken                           IAVLLFFIIILCWAQLGVIALKCETV..RKKKRHITIACSIFT.VVAIVAVVLFIQDGSISMNQIGLAFTILPAGLLMVLQYSIFKMVLD
Northern_mallard                  ISVLLFLIVVFYWAQIGVIALKCETV..HKNKTYVAVGCAIFT.VLAVIAVVLFIQGGNITINKVGLGFVVLPAVILMVLQYYIFKIVLV
American_alligator                IILFLFLAIIFSWAQLCVVGSKFEIG..LGKKVGLIITGSIII.IAGVVGAILLVPDGYTPKNRAGLGLLVVPAVIFLPLQYLLLGIVFE
Band_tailed_pigeon                IIVLLLLVIIFCSAQFSFIALKYEIE..PQKKTGIIVGGVIFI.VAAVVGTVLFAQDGYAAQNQAGLGLIVVPAMIAVPLQYFMFGIVFD
Rock_dove                         IIVLLLLVIIFCSTQFSFIALKYEIE..PQKKTGIIVAGVIFI.VAAIVGTVLFAQDGYTAQNQAGLGLIVVPAMIAVPLQYFLFKIVLD
Collared_flycatcher               IVSLVILLVILCAAQLTFIGLKYEIE..SQRKVCIIVALVIFA.VVAAVGAALFIQDGYTVQNQAGLGLMVIPAVILVPLQYVMFGIVFD
Bengalese_finch                   IVSLMCLLVILCVAQLCVIGLKYEIE..SQRLVYMIVALVIFA.VVAGVGAALFIPDGYTVRNQAGLGLIVIPALILVPLQYFMFGIVFD
Zebra_finch                       IVSLMCLLVILCVAQLCVIGLKYEIE..SQRLVYMIVALVIFA.VVAGVGAALFFPDGYTVRNQAGLGLIVIPAVILVPLQYFMFGIVFD
Burrowing_owl                     IIALVFLVIILCSAQFSIIALKYEIV..PQKKRGIIVAGIIFT.AAAVVGTVLFVQDDYNAQNQSGLGLIVLPAVILVPLQYFMFGMVFD
Bar-tailed_godwit                 ................AALKYEIE..TQKRMGIIIPGVIAT.VAAIVGTALFVQDGYTVQHQTGLGLIVFPAVIFIPLQYFMFGIVFD
Golden_eagle                      IIALLFLVIILCSAQLSVIALKYEIV..PKKKTGILVASFIFT.VAAVVGTVLFVQGGYTAQNQAGLGLIVVPAVILVPLQYLMFGIVFE
Kakapo                            IIALLFLVITLCAAQSSVIVLKYETV..PQKKTGIIVAVIIFV.VAAVVGTVLFVQDGYTAQNQAGLGLIVVPAVILVPLQYVMFGIVFD
Northern_Bobwhite                 IAALLVFIIVLCWTQLGVVAFKCETV..RKKKKHITIPCSIFT.VIAVVAVVLFIQDGSISMNQVGLAFTVLPAGMLMVLQYSIFKLILD
Barn_swallow                      IISLVCLLVILCAAQLSVIGLKYEIE..SQRKVCIIVALVIFA.VVAGVGAALFIQDGYTAQNQAGLGLIVIPAVILVPLQYVMFGIVFD
Ring-necked_pheasant              IAVLLVFIIVLCWAQLGVIAFKCETV..RKKKKHITIACSIFT.VIAVVAVVLFIQDGSISMNQIGLAFTVIPAGMLMVLQYSIFKMVLD
Common_box_turtle                 IILSMVLAILFYWIQLAVTVSKFETT..L.LKIGLFIAGVVIT.ISAVVGFSLLISDDFKPENQIGLGLIVLPAVILVPCQYFLLQIVYE
Big-headed_turtle                 IILSMVLAILIYWIQLAVTGSKFETT..L.LKIGLFIAGVVIT.VFAVVGFSLLISDEFKPENQIGLGLIVLPAVILVPPQYFLLKIVRE
Eastern_brown_snake               IISIIVLIVILYWCQFITVAQKFDMT..FEKKIGFTVTGLLIS.IFAVIGTILYAQGGCTTSVRTGIGLIVIPAVIVVPLLYFLFTSVFE
Eastern_diamondback_rattlesnake   IISIIILIVILYWCQFITVAIKFDME..FEKKIGLSFTGLLIT.IFAVTGTILFAHGGYIALVKAGIGIIVIPVIIVVPLLYFLFTSVFE
South_American_coral_snake        IISIIVLIVILYWCQFITVAQKFDMT..FKKKIGFTVTGLLIS.IFAVIGTILYAQGRYTASVRTGIGLIVIPAIIVVPLLYFLFTSVFE
Brown_tree_snake                  IISIIILIVVLYWCQFIIVAKKFDMT..FKKKIGLSVTGLLIS.IFAVIGTIFYAQGGYTALVKTGIGLIVIPAIIVVPLLYFLFTSVFE
MacQueens_bustard                 IITLLFSVIILCSAQLSAIALKYEVV..T.KKMSLIVAGIIFTATAAVVGTVLFVPDGYTAQNQAGLGLIVVPAVTLVPLQYFMFGIVSD
Cuckoo_roller                     IIALLFLVVILCLAQLSVIVLKYDIE..SQKKVGIIVTGVIFT.VAAVVGTVLFVQDGYTAQSQAGLGLIVVPAVILVPLQYFMFGIVFD
Hoatzin                           IIALLFLVLVLCSAQLSVIALKYEIV..PQKKMGIIVAGIIFT.VAAVVGTVLFVQDGYTAQNQAGLGLIVVPAVILVPLQYFMFGIVFD
Adelie_penguin                    IIALLFLVIILCSAQLSVIALKYEIV..PQKKMGMIVAVTIFT.VAAVVGTVLFVQDGYTAQNQAGLGLIVVPAVILVPLQYFVFGIVFD
Speckled_mousebird                IIALLFLVNVLCLAQLSVIVLKYDTV..TQKKIVIILASIIVT.VAAVVGTVLFVQEGYSVQNQTGLGLVVVPAVFLVLVQYFMFGIVFD
Golden-collared_manakin           IISLLCIVIILCAAQLTVIALKYDIE..FQRKVCIIVAVVILA.VVAGVGTALFVQDGYTALNQAGLGLIVVPAGILVPLQYFMFGIVFD
White-throated_tinamou            IIVLLFLAIIFCWTQLSVIASKFETV..FQKKISIIIACSIFT.IVAIAGVILFVPDGYTPGTQAGLGLIVIPALILLPLQCLLFGMVLD
Red-throated_loon                 IIALLFLVVILCLAQLSVIALKYEIE..PQKKTGIIVAGIIFA.VAAVVGTVLFLQDGYTAQNQAGLGLIVVPAVILVPLQYFMFGIVFD
Red-crested_turaco                IIALLFLVIILCWAQLSVIGLKYEIV..PPKKMGIIAAGIISI.VAAVVGTAFFIQDGYTAQSQAGLSLIIVPAVILVPLQWSMFGIVFD
Great_crested_grebe               IIVLLFLVIILCSAQLSVIALKYEIE..PPKKMGIIVAGVILT.VAAVVGTVLFVQDGYTVQNQAGLGLIVLPAVILVLLQCCMFQIVFD
Northern_carmine_bee-eater        IIALLFLVIILCSAQLSVLALKYE.....KKVGLILGVAIFT.VAAVVGTVLFVQDGYTAQNQAGLGLIVVPAVILVPLQYFMFGIVFD
Northern_fulmar                   IIALLFLVIILCSAQLSVIALKYEIV..PQKKLGFIVGGIIFT.AAAVVGTVLFVQDGYAAQNQAGLGLIVVPAVILVPLQYFMFGIVFD
Emperor_penguin                   IIALLFLVIILCSAQLSVIALKYEIV..PQKKMGMIVAGIIFT.VAAVVGTVLFVQDGYTAQNQAGLGLIVVPAVILVPLQYFMFGIVFD
White-tailed_eagle                IIALLFLVIILCSAQLSVIALKYEIV..PQKKTGILVASFIFT.VAAVVGTVLFVQGGYTAQNQAGLGLIVVPAVILVPLQYLMFGVVFE
Dalmatian_pelican                 IIALLILAIILCLAQLRVTAFKYDIV..PQKKMSFIVAGVVVT.IAAVVGTVLFVQDGYIVQKQAGLGLIVGPAVILVPLQYFIFGIVFD
Grey_crowned_crane                IIVLLFSVVVLCAAQFIVITLKYEIV..PQKKVGIIVPVIIFT.VAAVVGTVLFVQDGYSAQNQAGLGLIVVPAVIFVPLQYFIFGLIFD
Common_cuckoo                     IIALLLFVAGLCLAQFIFVAIKYGLG..PQKKICFIGTGFIFT.VAAVVGIVFFVQDGYNTQSQAGFGLLVVPVVVAVPLQYLMFGIVFD
Sunbittern                        IVALLFLVTILCLVQLCFIALKYEIV..SQKKMGIIAAGVIFT.IAAVVGTVLFVQEGYTAQNQAGLGLIVVPAVILVPLQYFMFGIVFD
American_crow                     IISLMSLLVVLCAAQLSVIGLKYEIE..SQRKVCSIVALVIFA.VVAGVGAALFVQDGYTVQSQAGLGLTVIPAVILVPLQYVMFGIVFD
Brown_mesite                      IIALLISVIILCAAQLSVIALKYEIV..PQKKTVTITAGIIIV.VSAVVGTILFAQDGYTAQNQAGLGLIVIPAVIVVLLQYFMFGMVFD
Barn_owl                          IIALLFLVIILCSAQFSVIALKYEIV..PQKKRGIIVAGVIFT.VAAVVGTVLFVQDGYTAQNQAGLGLIVVPAVILVPLQYFMFGFVFD
American_flamingo                 IIVLLFLVIILCSAQFSVTVLKYEIV..HQKKMGIIVAGVIFT.VAAVVGTVLFVRDGYTAQNQAGLGLIVVPAVIVVPLQFFIFGIVFD
Annas_hummingbird                 IIVLLFLVIILCSAQLIVIVLKYEIE..TSMKTGIFVAGIIFT.VAAVIGTVLFVQDGYAAQNQAGLGLIVVPAVILVPLQYLMFGIVFN
Red-legged_seriema                IIALLFLVIVLCSAQLSVLVLKYEIV..PQKKTGILVAGIILT.AAAVVGTVLFVQDGYTAQNQAGLGLIVVPAVVLVPLQYFMFGIVFD
Gouldian_finch                    VVSLMCLLVILCVVQLCVIGLKYEIE..SQRLVYMIVALVIFA.VVAGVGAALFIPDGYTVQNQAGLGLIVIPAVILVPLQYVMFGIVFD
Little_egret                      IIVLLFLVIIFCSAQLSVIALKYGTL..PQKKMGIVVAGITIT.LAAVVGTVLFVQDGYNVQNQAGLGLIVVPALILVPLQYFMFGYVFD
Yellow-throated_sandgrouse        IIALLFLVIILCSAQFTVIALKYEIE..SQKKMGIIVAGIIFT.AAAVVGTVLFVQDGYTAQNQAGLGLIVVPAVILVPLQYFMFGIVFD
Turkey_vulture                    IIALLFLVIVLCSAQLSVIALKYEIV..SQKKIGILVAGFIFT.VAAVVGTILFVQDGYTAQNQAGLALIVVPAVILVPLQYFMFGIVFD
Downy_woodpecker                  IIVLLFLIIVLCSAQLSVVAFKYEIV..PQKKVSIAVAGFIFT.VAAVIGTVLYVQDGFTAQNQAGLGLIVIPAVSMVPLQYFIFRIAFD
Kea                               IIALLFLVITLCVAQSSVIVLKYETV..SQKKTGIIVAVIIFI.VAAVVGTVLFVQDGYTAQNQAGLGLIVVPAVILVPLQYVMFGIVFD
Killdeer                          IIMLLFLVIILCSAQLIVIALKYEIA..PQKRMGVIVAGVIST.VAAVVGTVLFVQDGYTAQNQAGLGLIVVPAVILVPLQYFMFGMVFD
Scaled_quail                      IAALLVFIIVLCWTQLGVVAFKCETV..RKKKKHITIPCSIFT.VIAVVAVVLFIQDGSISMNQVGLAFTVLPAGMLMVLQYSIFKLILD
Ocelot_gecko                      IIVTMILAAIFYLIQLGFVAIKYGIP..VRKKITLTIAGLIVI.TAAVIGYVLLVPEGFTESNQIGLSLIVLPAVILVPLLYILFDLVFE
Common_wall_lizard                VIGAAILAVALFLLQVGYVAVKFEMS..LLKKISFGLVALIVM.IIVVPGAALLVKEGFTLASQFGLGLVVLPTILLVPPLFFVFQSVFE
consensus>70                      ii.ll.lviilc..q..vi..kye......kk....va..i...v.avvg.vlfvqdgy...nq.Glgli!vpavilvplqy.mf..vfd
```

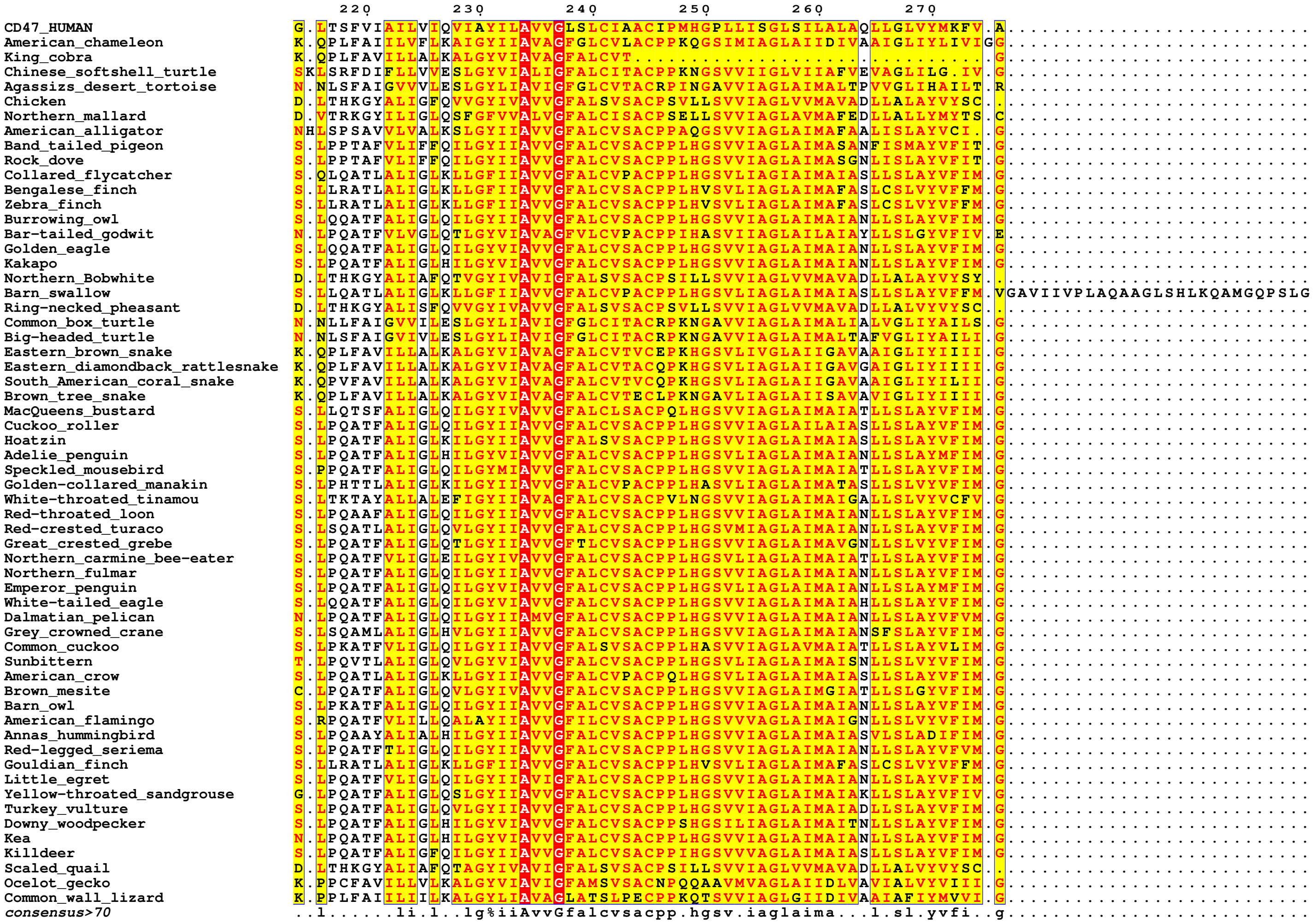
220
230
240
250
260
270
CD47_HUMAN
American_chameleon
King_cobra
Chinese_softshell_turtle
Agassizs_desert_tortoise
Chicken
Northern_mallard
American_alligator
Band_tailed_pigeon
Rock_dove
Collared_flycatcher
Bengalese_finch
Zebra_finch
Burrowing_owl
Bar-tailed_godwit
Golden_eagle
Kakapo
Northern_Bobwhite
Barn_swallow
Ring-necked_pheasant
Common_box_turtle
Big-headed_turtle
Eastern_brown_snake
Eastern_diamondback_rattlesnake
South_American_coral_snake
Brown_tree_snake
MacQueens_bustard
Cuckoo_roller
Hoatzin
Adelie_penguin
Speckled_mousebird
Golden-collared_manakin
White-throated_tinamou
Red-throated_loon
Red-crested_turaco
Great_crested_grebe
Northern_carmine_bee-eater
Northern_fulmar
Emperor_penguin
White-tailed_eagle
Dalmatian_pelican
Grey_crowned_crane
Common_cuckoo
Sunbittern
American_crow
Brown_mesite
Barn_owl
American_flamingo
Annas_hummingbird
Red-legged_seriema
Gouldian_finch
Little_egret
Yellow-throated_sandgrouse
Turkey_vulture
Downy_woodpecker
Kea
Killdeer
Scaled_quail
Ocelot_gecko
Common_wall_lizard
consensus>70
..l......li.l..lg%iiAvvGfalcvsacpp.hgsv.iaglaima...l.sl.yvfi..g

```
                                           280        290        300
CD47_HUMAN                          .......SNQKTIQPPRKAVEEPLNAFKESK.G................MMND......E...
American_chameleon                  .......SSLRDHQPPRKAVEESLND...AK.GV................MLE..........
King_cobra                          .......SNLKDHQPPRKAVEESLNG...RCTGYEVIESNYGHGNKWRETFVD....TPWP..
Chinese_softshell_turtle            .......SLKAYI.GPLTPN...........................................
Agassizs_desert_tortoise            .......SSKKDQ.ASTKAVEEPLNG...RW................................
Chicken                             .......SRMKDHQTPRY.............................................
Northern_mallard                    .......SRMKDHQTPRKAVEEPLNE...LK.T................ETESTAEMNDG...
American_alligator                  .......SNTKDHQPPRNNL...........................................
Band_tailed_pigeon                  .......SRMKDHPRPGKAVEEPLND...AK.G................VML.......E...
Rock_dove                           .......SRMKDHPRPGVSLK.LINL.................................G...
Collared_flycatcher                 .......SRMKDHPRPGKAVEEPLND...AK.G................VML.......E...
Bengalese_finch                     .......SRMKDHPRPGKAVEEPLND...AK.G................VML.......E...
Zebra_finch                         .......SRMKDHPRPGKAVEEPLND...AK.G................VML.......E...
Burrowing_owl                       .......SRMKDRPRPGKAVEEPLND...AK.G................VML.......E...
Bar-tailed_godwit                   .......LKSYVIREKYTVVDTPWP......................................
Golden_eagle                        .......SRMKDHPRPGKAVEEPLND...AK.G................VML.......E...
Kakapo                              .......SRMKDHPRPGKAVEEPLND...AK.G................VML.......E...
Northern_Bobwhite                   .......R.IAEAIQ................................................
Barn_swallow                        SVELDTGKRITHGIKYGRSV...........................................
Ring-necked_pheasant                .......SRMKDHQTPRKAVEEPLND...TK.G................VML.......E...
Common_box_turtle                   .......YSKKEQ.PPRKAVEEPLNA...KV.TG...............ESDR..ICYRGGDM
Big-headed_turtle                   .......SSKKEQ.HPRKAVEEPLNG.....................................
Eastern_brown_snake                 .......SNLKDHQPPRKAVEESLND...AK.G................VMLE..........
Eastern_diamondback_rattlesnake     .......SNLKDHQPPRHWM...........................................
South_American_coral_snake          .......SNLKDHQPPRKAVEESLND...AK.G................VMLE..........
Brown_tree_snake                    .......SNLKDHQPPRKAVEESLND...AK.G................VMLE..........
MacQueens_bustard                   .......SRMQDHPRPGKAVEEPLN......................................
Cuckoo_roller                       .......SRMKDHHRPGKAVEEPLN......................................
Hoatzin                             .......SRMKDHPRPG..............................................
Adelie_penguin                      .......SRMKDHPRPGKAVEEPL...................................N...
Speckled_mousebird                  .......SRMKDHPRPGKAVEEPLN......................................
Golden-collared_manakin             .......SRMKDHPRPGKAVEEPLN......................................
White-throated_tinamou              ...............................................................
Red-throated_loon                   .......SRMKDHPRPGKAVEEPLN......................................
Red-crested_turaco                  .......SRMKDHPRPGKAVEEPLN......................................
Great_crested_grebe                 ...............................................................
Northern_carmine_bee-eater          .......PRMKDHPRPGKAVEEPLN......................................
Northern_fulmar                     .......SRVKDHPRPGKAVEEPL...................................N...
Emperor_penguin                     .......SRMKDHPRPGKAVEEPL...................................N...
White-tailed_eagle                  .......SRMKDHPRPGKAVEEPLN......................................
Dalmatian_pelican                   .......SRMKDHPRPG..............................................
Grey_crowned_crane                  .......SRMKDHHRPGKAVEEPLN......................................
Common_cuckoo                       .......SRMKDHPRPG..............................................
Sunbittern                          .......SRMKDHPRPGKAVEEPLN......................................
American_crow                       .......SRMKDHPHPGKAVEEPL...................................N...
Brown_mesite                        .......SSMKDHPRPGKAVEEPLN......................................
Barn_owl                            .......SRMKDHPRPGKAVEEPLN......................................
American_flamingo                   ...............................................................
Annas_hummingbird                   .......SRMKDHPRPGKAVEEPL...................................N...
Red-legged_seriema                  .......SRMKDHPRPGKAVEEPLN......................................
Gouldian_finch                      .......SRMKDHPRPGNCRVM.........................................
Little_egret                        .......SSMKDHPRPGKAVEEPL...................................N...
Yellow-throated_sandgrouse          .......SRMKDHPRPGKAVEEPLN......................................
Turkey_vulture                      .......SRMKDHPRPGKAVEEPL...................................N...
Downy_woodpecker                    .......SRMKDHPRPGKAVEEPL...................................N...
Kea                                 .......SRMKDHPRPGKAVEEPLN......................................
Killdeer                            .......SRMKDHPRPGKAVEEPLN......................................
Scaled_quail                        ..............K................................................
Ocelot_gecko                        .......FNFKEHQPPRHVMS..V....................................N..
Common_wall_lizard                  .......TRFKDHQPPKQKSKAIWQ....WT.WK................QVE....KDFC..
consensus>70                        .......s.mkdh..p.kavee.l.......................................
```
