# Supplementary material for: Structure of the human marker of self 5-transmembrane receptor CD47
Source: Nat Commun. 2021 Sep 1;12:5218. doi: 10.1038/s41467-021-25475-w (PMC8410850; doi:10.1038/s41467-021-25475-w)
Supplement: Supplementary file 7 — Supplementary Data 4 [file 41467_2021_25475_MOESM7_ESM.pdf]

```
                                      1         10        20        30        40        50        60          70
CD47_HUMAN         .................QLLFNKTKSVEFTFCNDTVVIPCFVTNMEAQNTTEVYVKWKFKGRDIYTFDGALNKSTVPTDF.SSAKIEVSQ
Variola_virus      MLRV...RILL.IYLCTFVVITSTKTIEYTACNDTIIIPCTIDNP......TKYIRWKLDNHNILTYNKTS.KTIILSKWHTSAKL..HS
Ectromelia_virus   MLRV...RISL.IYLYTLVVITTTKTIEYTACNDTIIIPCTIDNP......TKYIRWKLDNHDILTYNKTS.KTTILSKWHTSARL..HS
Cowpox_virus       MSRV...RISL.IYLYTLVVITTTKTIEYTACNDTIIIPCTIDNP......TKYIRWKLDNHDILTYNKTS.KTTILSKWHTSARL..HS
Monkeypox_virus    MLRV...RILL.IYLCTFVVITSTKTIEYTACNDTIIIPCIIDNP......TKYIRWKLDNHDILTYNKTS.KTTILSKWHTSARL..HS
Akhmeta_virus      MLRV...RISL.IYLYTLFVITTTKTIEYTACNDTIIIPCTIDNP......TKYIRWKLDNHDILTYNKTS.KTTILSKWHTSARL..HS
Yokapox_virus      ML......LKL.FYLYTVTYIANSKTIEYTPCNDTIIIPCYISNP......TKYITWKLDNHVIIIYNKTT.SSTMLSKWHKSAKL..RL
Taterapox_virus    MLRV...RILL.IYLCTFVVITSTKTIEYTACNDTIIIPCTIDNP......TKYIRWKLDNHDILTYNKTS.KTTILSKWHTSAKL..HS
Skunkpox_virus     MENS...KLLLVIYFTLFVDIIKTKTIEYTACNDTIIIPCTIDNP.......KYIRWKLDNHDILIYNMTS.KTTILSKWHTSARL..HS
Volepox_virus      MHKITNSKLLLVIYLTL.FDIVTTKTIEYTACNNTVIIPCTIDNP.......TYVRWRLDNHDILIYNRTS.KTTILSKWHTSARL..HS
Camelpox_virus     MLRV...RILL.IYLCTFVVITATKTIEYMACNDTIIIPCTIDNP......TKYIRWKLDNHDILTYNKTS.KTTILSKWHTSAKL..HS
Horsepox_virus     MLRV...RILL.IYLCTFMIITSTKTIEYTACNNTIIIPCTIDNP......TKYIRWKLDNHDILTYNKTS.KTTILSKWHTSARL..HS
Alaskapox_virus    MIR.....ISL.IYIYMLIVMTTTKNIEYTACNDSIIIPCTIDNP......TKYIRWKFDNHDILTYNKTS.KMSILSKWHTSAKL..HS
consensus>70       m.........l.iyl.....i..tKt!E%taCN#t!!IPCt!dNp......tkY!rWkldnhdIlt%#kts.kttilskwhtSA.l..hs

                       80        90        100       110       120       130       140       150       160
CD47_HUMAN         LLKGDASLKMDKSDAVSHTGNYTCEVTELTREGETIIELKYRVVSWFSPNENILIVIFPIFAILLFWGQFGIKTLKYRSGGMDEKTIALL
Variola_virus      LSDNDVSLIIKYKDIL..PGTYTCEDNTGI...KSTVKLVQRHTNWFNDHHTMLMFIFTGITLFLLFLEIAYTSISVV...FSTNLGILQ
Ectromelia_virus   LSDNDVSLIMEYKDIL..PGTYTCGDNTGI...KSTVKLVQCHTNWFNDYQTTLMFIFTGITLFLLFLEIAYTSISVV...FSTNLGILQ
Cowpox_virus       LSDSDVSLIMEYKDIL..PGTYTCEDNTGI...KSTVKLVQLHTNWFNDYQTMLMFIFTGITLFLLFLEITYTSISVV...FSTNLGILQ
Monkeypox_virus    LSDSDVSLIMEYKDIL..PGTYTCGDNTGI...KSTVKLVQRHTNWFNDYQTMLMFIFTGITLFLLFLEIAYTSISVV...FSTNLGILQ
Akhmeta_virus      LSDSDVSLIMEYKDIL..PGTYTCEDNTGI...KSTVTLIQRHINWFNDHQTMLMFIFTGVTLFLLFLEITYTSISVV...FSTNLGILQ
Yokapox_virus      ASENDMSLIIKYKDIL..PGNYTCSDSTGV...KSNIQLVFKETHWFNDYDTMLIFIFSGVTLVLLLQVIYTSISIR...FTNNVGILQ
Taterapox_virus    LSDNDVSLIIEYKDIL..PGTYTCEDNTGI...KSTVKLVQRHTNWFNDHHTMLMFIFTGITLFLLFLEIAYTSISVV...FSTNLGILQ
Skunkpox_virus     LADNDISLIMEYKDIL..PGNYTCENNKGI...KHTVKLIQRHTNWFNDHQTMLMFIFTGVTLLLLMSQIAYTSISVI...YSTNLGILQ
Volepox_virus      LADNDISLIMEYKDIL..PGNYTCDNNKGI...KYTVNLIQRHTNWFNDHQTMLMFIFTGITLLLLVAQIAYTSISVI...YSTNMGILQ
Camelpox_virus     LSDNDVSLIIEYKDML..PGTYTCEDNTGI...KSTVKLVQRHTNWFNDHHTMLMFIFTGITLFLLFLEIAYTSTSVV...FSTNLGILQ
Horsepox_virus     LSDSDVSLIIEYKDIL..PGTYTCGDNTGI...KFTVKLVQRHTNWFNDYQTMLMFIFTGITLFLLFLEIAYTSISVV...FSTNLGILQ
Alaskapox_virus    LSDNDVSLIMEYKDIL..PGTYTCEDNTGI...KSNVTLIRRHTNWFNDHQIMLMFIFTGVTLFLLALEIMYTSISVV...FSTNLGILQ
consensus>70       lsd.DvSLi.eykDil..pG.YTC.dntgi...k.t!.LvqrhtnWFnd.qtmLmfIFtgitl.Ll.l#i.ytsisvv...fstnlgiLq

                        170       180       190       200       210       220       230       240       250
CD47_HUMAN         VAGLVITVIVIVGAILFVPGEYSLKNATGLGLIVTSTGILILLHYYVFSTAIGLTSFVIAILVIQVIAYILAVVGLSLCIAACIPMHGPL
Variola_virus      VFGCIIAMIELCGAFLFYPSMFTLRHIIGLLMMTLPSIFLIITKVFSFWLLCKLSCAVHLIIYYQLAGYILTVLGLGLSLKECV..DGTL
Ectromelia_virus   VFGCVIAMIELCGAFLFYPSMFTLRHIIGLLMITLPSIFLIITKVFSFWLLCKLSCAVHLIIYYQLAGYILTVLGLGLSLKECV..DGTL
Cowpox_virus       VFGCVIAMIELCGAFLFYPSMFTLRHIIGLLMMTLPSIFLIITKVFSFWLLCKLSCAVHLIIYYQLAGYILTVLGLGLSLKECV..DGTL
Monkeypox_virus    VFGCVIAMIELCGAFLFYPSMFTLRHIIGLLMMTLPSIFLIITKVFSFWLLCKLSCAVHLIIYYQLAGYILTVLGLGLSLKECV..DGTL
Akhmeta_virus      VFGCVIAMIELCGAFLFYPSMFTLRHIIGLLMMTLPSIFLIITKVFSFWLLCKLSCAVHLIIYYQLAGYILTVLGLGLSLKECV..DGTL
Yokapox_virus      FFCVIIIMIELSGSLLFYPSKFNMRHIIGLLMLTIPSIFLIVTKIFSFWVLNKLSFGVHVIIYYQLAGYVLTILGLGLSLKECV.IDGSL
Taterapox_virus    VFGCIIAMIELCGAFLFYPSMFTLRHIIGLLMMTLPSIFLIITKVFSFWLLCKLSCAVHIIIYYQLAGYILTVLGLGLSLKECV..DGPL
Skunkpox_virus     VFGCIIAMIEVSGAFLFYPSMFTLRHIIGLLMMTLPSIFLIITKVFSFWILSKLSCGVHLIIYYQLAGYILTVLGLGLSLKECV..DGTL
Volepox_virus      VFGCIVVMIEVSGVFLFYPPMFTLRHIIGLLMMTLPSIFLIITKVFSFWILSKLSCGVHLIIYYQLAGYILTVLGIGLSLKECV..DGTL
Camelpox_virus     VFGCIIAMIELCGAFLFYPSMFTLRHIIGLLMMTLPSIFLIITKVFSFWLLCKLSCAVHLIIYYQLAGYILTVLGLGLSLKECV..DGTL
Horsepox_virus     VFGCIIAMIELCGAFLFYPSMFTLRHIIGLLMMTLPSIFLIITKVFSFWLLCKLSCAVHLIIYYQLAGYILTVLGLGLSLKECV..DGTL
Alaskapox_virus    VFGCVIAMVELCGAFLFYPPMFTLRHIIGLLMMTLPSIFLIITKVFSFWLLCKLSCSVHLIIYYQLSGYILTVLGLGLNLKECA..DGTL
consensus>70       vfgc!!am!el.GafLFyPsm%t$rhiiGLl$mtlpsifLIitkv%sFw.l.kLsc.VhlIiyyQlagY!Lt!lGlgLslkeCv..dGtL

                        260       270       280       290       300
CD47_HUMAN         LISGLSILALAQLLGLVYMKFVASNQKTIQPPRKAVEEPLNAFKESKGMMNDE
Variola_virus      LLSGLGTIMVSEHFSLLFLVCFPSTQRDYY.......................
Ectromelia_virus   LLSGLGTIMVSEHFSLLFLVYFPSTQRDYY.......................
Cowpox_virus       LLSGLGTIMVSEHFSLLFLVCFPSTQRDYY.......................
Monkeypox_virus    LLSGLGTIMVSEHFGLLFLVCFPSTQRDYY.......................
Akhmeta_virus      LLSGLGTIMVSEHFSLLFLVCFPSTQRDYY.......................
Yokapox_virus      LLSGLGIISISEHFSLLLLVCFQSIHREYY.......................
Taterapox_virus    LLSGLGTIMVSEHFSLLFLVCFPSTQRDYY.......................
Skunkpox_virus     LLSGLGTIMVSEHFSLLFLVCFPSAQRDYY.......................
Volepox_virus      LLSGLGTIMVSEHFSLLFLVCFPSTQRDYY.......................
Camelpox_virus     LLSGLGTIMVSEHFSLLFLVCFPSTQRDYY.......................
Horsepox_virus     LLSGLGTIMVSEHFSLLFLVCFPSTQRDYY.......................
Alaskapox_virus    LLSGLGTIMVSEHFSLLFLVCFPSTQRDYY.......................
consensus>70       LlSGLgtimvs#hfsLlf$vcfpStqrdyy.......................
```
