# Supplementary figures and images for: Structure of the human marker of self 5-transmembrane receptor CD47
Source: Nat Commun. 2021 Sep 1;12:5218. doi: 10.1038/s41467-021-25475-w (PMC8410850; doi:10.1038/s41467-021-25475-w)

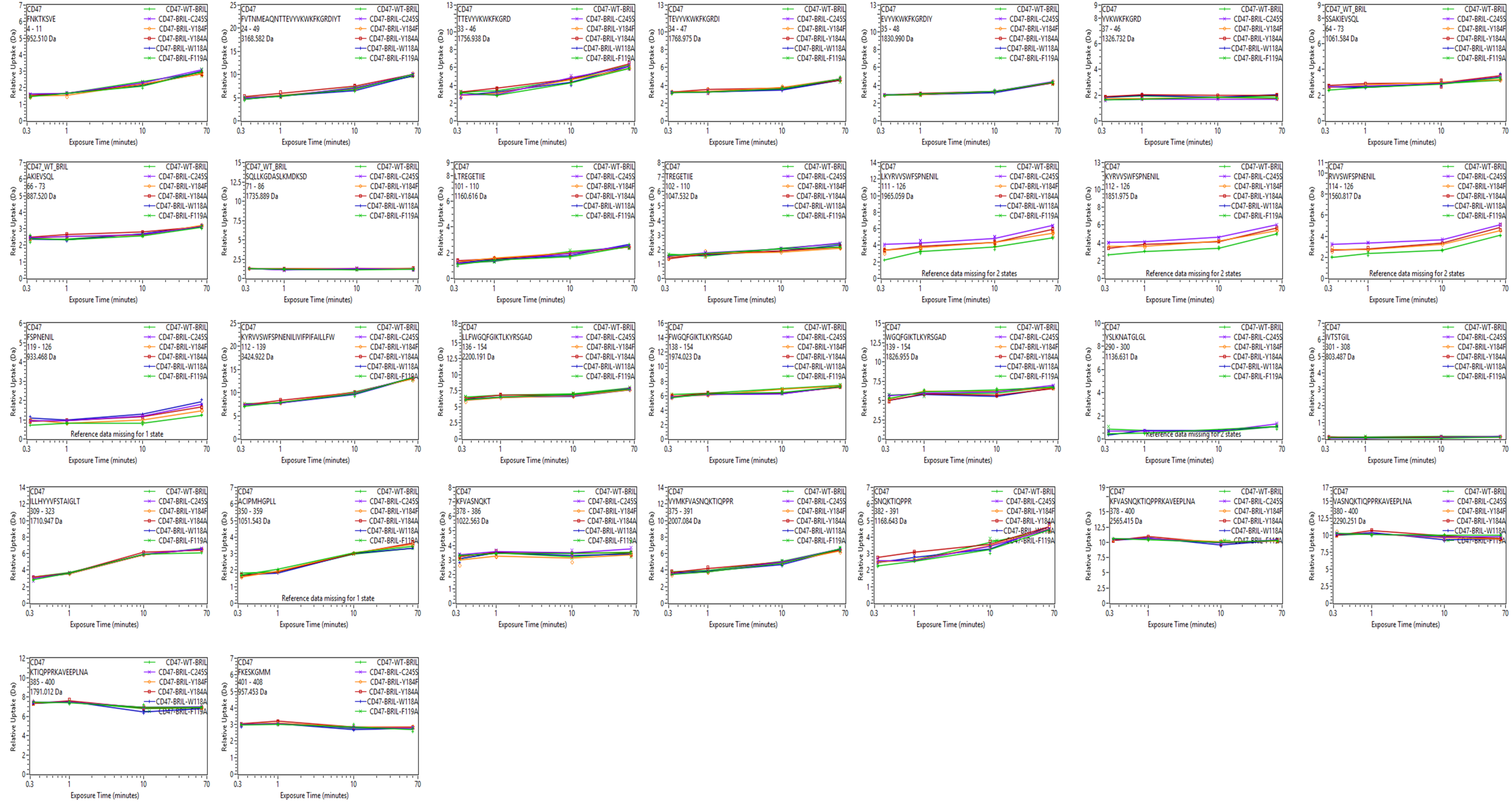

Supplement: Supplementary file 9 — Supplementary Data 6 [file 41467_2021_25475_MOESM9_ESM.pdf]
