# Supplementary material for: Structure of the human marker of self 5-transmembrane receptor CD47
Source: Nat Commun. 2021 Sep 1;12:5218. doi: 10.1038/s41467-021-25475-w (PMC8410850; doi:10.1038/s41467-021-25475-w)
Supplement: Supplementary file 6 — Supplementary Data 3 [file 41467_2021_25475_MOESM6_ESM.pdf]

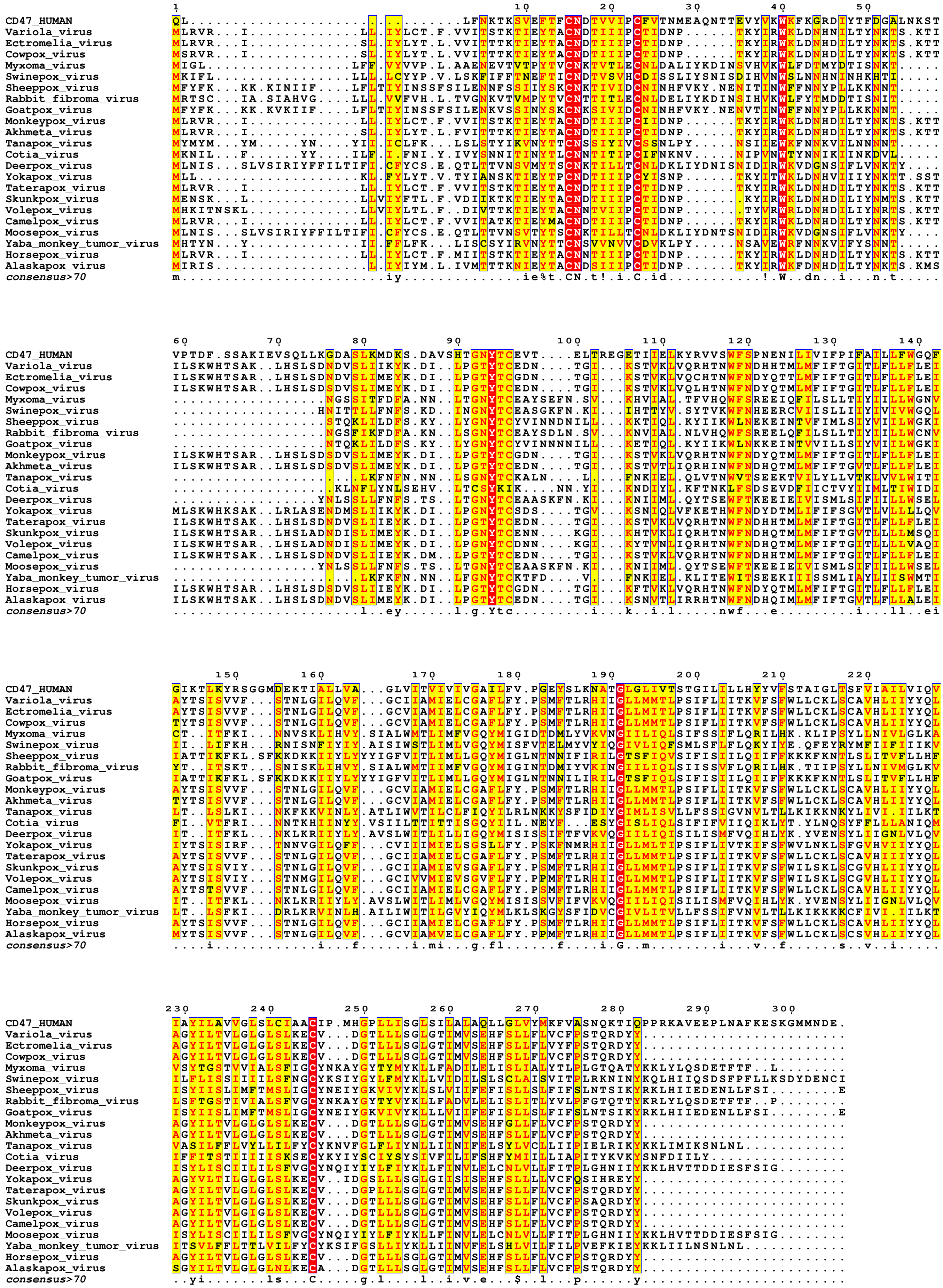

1 10 20 30 40 50
CD47_HUMAN QL....................L.L.......LFNKTKSVEFTFCNDTVVIPCFVTNMEAQNTTEVYVKWKFKGRDIYTFDGALNKST
Variola_virus MLRVR...I.............LL.IYLCT.F.VVITSTKTIEYTACNDTIIIPCTIDNP......TKYIRWKLDNHNILTYNKTS.KTI
Ectromelia_virus MLRVR...I.............SL.IYLYT.L.VVITTTKTIEYTACNDTIIIPCTIDNP......TKYIRWKLDNHDILTYNKTS.KTT
Cowpox_virus MSRVR...I.............SL.IYLYT.L.VVITTTKTIEYTACNDTIIIPCTIDNP......TKYIRWKLDNHDILTYNKTS.KTT
Myxoma_virus MIGL................LFF.VYVVP.L.AAENEVTVTPYTVCNKTVTLECNLDALIYKDINSVHVKWLFDTMYDTISNKT.....
Swinepox_virus MKIFL...............LLL.LCYYP.V.LSKFIFFTNEFTICNDTVSVHCDISSLIYSNISDIHVNWSLNNHNINKHTI.....
Sheeppox_virus MFYFK...KK.KINIIF....LFLTIYINSSFSILENNFSSIIYSKCNKTIVIDCNINHFVKY.NENITINWFFNNYPLLKKNNT....
Rabbit_fibroma_virus MRTSC...IA.SIAHVG....LLL.VVFVH.L.TVGNNKVTVMPYTVCNTTITLECNLDELIYKDINSIHVKWLFYTMDDTISNIT.....
Goatpox_virus MFYFK...KK.KVKIIF....LFLTIYINSSFSILENKVSSINYSKCNKSIVIDCNINHFVKY.NENVTINWFFNNYPLLKKNNT....
Monkeypox_virus MLRVR...I.............LL.IYLCT.F.VVITSTKTIEYTACNDTIIIPCIIDNP......TKYIRWKLDNHDILTYNKTS.KTT
Akhmeta_virus MLRVR...I.............LL.IYLYT.L.FVITTTKTIEYTACNDTIIIPCTIDNP......TKYIRWKLDNHDILTYNKTS.KTT
Tanapox_virus MYMYM...YM.....YN....YII.ICLFK...LSLSTYIKVNYTTCNSSIYIVCSSNLPY.....NSIIEWKFNNKVILNNNNT.....
Cotia_virus MKNIL...F......YY....ILF.I.FNI.Y.IVYSNNITINYTLCNNTITIPCIFNKNV.....NIPVNWTYNNIKIINKDVL.....
Deerpox_virus MLNIS...SLVSIRIYFFILTIFI.CFYCS.E.QTLTTVNSVMYTSCNKTILLTCNLDKLIYDNISNIDIRWKVDGNSIFLVNKTY....
Yokapox_virus MLL..................KL.FYLYT.V.TYIANSKTIEYTPCNDTIIIPCYISNP......TKYITWKLDNHVIIIYNKTT.SST
Taterapox_virus MLRVR...I.............LL.IYLCT.F.VVITSTKTIEYTACNDTIIIPCTIDNP......TKYIRWKLDNHDILTYNKTS.KTT
Skunkpox_virus MENSK...L.............LLVIYFTL.F.VDIIKTKTIEYTACNDTIIIPCTIDNP......KYIRWKLDNHDILIYNMTS.KTT
Volepox_virus MHKITNSKL.............LLVIYLTL.F..DIVTTKTIEYTACNNTVIIPCTIDNP......TYVRWRLDNHDILIYNRTS.KTT
Camelpox_virus MLRVR...I.............LL.IYLCT.F.VVITATKTIEYMACNDTIIIPCTIDNP......TKYIRWKLDNHDILTYNKTS.KTT
Moosepox_virus MLNIS...SLVSIRIYFFILTIFI.CFYCS.E.QTLTTVNSVTYTSCNKTILLTCNLDKLIYDNTSNIDIRWKVDGNSIFLVNKTY....
Yaba_monkey_tumor_virus MHTYN...Y.............II.FFLFK...LISCSYIRVNYTTCNSVVNVVCDVKLPY.....NSAVEWRFNNKVIFYSNNT.....
Horsepox_virus MLRVR...I.............LL.IYLCT.F.MIITSTKTIEYTACNNTIIIPCTIDNP......TKYIRWKLDNHDILTYNKTS.KTT
Alaskapox_virus MIRIS.................L.IYIYM.L.IVMTTTKNIEYTACNDSIIIPCTIDNP......TKYIRWKFDNHDILTYNKTS.KMS
consensus>70 m.......................iy.............ie%t.CN.t!.i.C.id..........!.W..dn..i...n.t.....

60 70 80 90 100 110 120 130 140
CD47_HUMAN VPTDF.SSAKIEVSQLLKGDASLKMDKS.DAVSHTGNYTCEVT....ELTREGETIIELKYRVVSWFSPNENILIVIFPIFAILLFWGQF
Variola_virus ILSKWHTSAK..LHSLSDNDVSLIIKYK.DI..LPGTYTCEDN....TGI...KSTVKLVQRHTNWFNDHHTMLMFIFTGITLFLLFLEI
Ectromelia_virus ILSKWHTSAR..LHSLSDNDVSLIMEYK.DI..LPGTYTCGDN....TGI...KSTVKLVQCHTNWFNDYQTTLMFIFTGITLFLLFLEI
Cowpox_virus ILSKWHTSAR..LHSLSDSDVSLIMEYK.DI..LPGTYTCEDN....TGI...KSTVKLVQLHTNWFNDYQTMLMFIFTGITLFLLFLEI
Myxoma_virus ..................NGSSITFDFA.NN..LTGNYTCEAYSEFN.SV...KHVIAL.TFVHQWFSREEIQFILSLLTIYIILLWGNV
Swinepox_virus .................HNITTLLFNFS.KD..INGNYTCEASGKFN.KI...IHTTYV.SYTVKWFNHHEERCVIISLLIIYIVIVWGQL
Sheeppox_virus ..................STQKLILDFS.KY..LYGNYTCYVINNDNILL...KKTIQL.KYIIKWLNEKEINTVFIMLSIYVIILWGKI
Rabbit_fibroma_virus ..................NGSFIKFDFA.KN..LSGNYTCEAYSDLN.SV...KNVIAL.NLVHQWFSREELQFILSLLTIYIILLWCNV
Goatpox_virus ..................NTQKLILDFS.KY..LYGNYTCYVINNNNILL...KETIQL.KYIIKWLNKKEINTVVILLSIYVIILWGKI
Monkeypox_virus ILSKWHTSAR..LHSLSDSDVSLIMEYK.DI..LPGTYTCGDN....TGI...KSTVKLVQRHTNWFNDYQTMLMFIFTGITLFLLFLEI
Akhmeta_virus ILSKWHTSAR..LHSLSDSDVSLIMEYK.DI..LPGTYTCEDN....TGI...KSTVTLIQRHINWFNDHQTMLMFIFTGVTLFLLFLEI
Tanapox_virus ...................KLNFLKFNFN.NN..LSGNYTCKALN....L....FNKIEL.QLVTNWVTSEEKTVILYLLVTKLVVLWITI
Cotia_virus ..................KLNFLYNLSEHV..LTCSYKIK.....NN.YI...KNDIYL.KFTNKLFSDSEVDFIICTVYIIMLTIWIDI
Deerpox_virus ................YNLSSLLFNFS.TS..LTGNYTCEAASKFN.KI...KNIIML.QYTSEWFTKEEIEIVISMLSIFIILLWSEL
Yokapox_virus MLSKWHKSAK..LRLASENDMSLIIKYK.DI..LPGNYTCSDS....TGV...KSNIQLVFKETHWFNDYDTMLIFIFSGVTLVLLLQV
Taterapox_virus ILSKWHTSAR..LHSLSDNDVSLIIEYK.DI..LPGTYTCEDN....TGI...KSTVKLVQRHTNWFNDHHTMLMFIFTGITLFLLFLEI
Skunkpox_virus ILSKWHTSAR..LHSLADNDISLIMEYK.DI..LPGNYTCENN....KGI...KHTVKLIQRHTNWFNDHQTMLMFIFTGVTLVLLMSQI
Volepox_virus ILSKWHTSAR..LHSLADNDISLIMEYK.DI..LPGNYTCDNN....KGI...KYTVNLIQRHTNWFNDHQTMLMFIFTGITLLLLVAQI
Camelpox_virus ILSKWHTSAK..LHSLSDNDVSLIIEYK.DM..LPGTYTCEDN....TGI...KSTVKLVQRHTNWFNDHHTMLMFIFTGITLFLLFLEI
Moosepox_virus ................YNLSSLLFNFS.TS..LTGNYTCEAASKFN.KI...KNIIML.QYTSEWFTKEEIEIVISMLSIFIILLWSEL
Yaba_monkey_tumor_virus ...................LKFKFN.NN..LSGNYTCKTFD....V....FNKIEL.KLITEWITSEEKIIISSMLIAYLIISWMTI
Horsepox_virus ILSKWHTSAR..LHSLSDSDVSLIMEYK.DI..LPGTYTCGDN....TGI...KFTVKLVQRHTNWFNDYQTMLMFIFTGITLFLLFLEI
Alaskapox_virus ILSKWHTSAK..LHSLSDNDVSLIMEYK.DI..LPGTYTCEDN....TGI...KSNVTLIRRHTNWFNDHQIMLMFIFTGVTLFLLALEI
consensus>70 ....l..ey......l.g.Ytc........i...k..i.l.....nwf...e.........i...ll..ei

150 160 170 180 190 200 210 220
CD47_HUMAN GIKTLKYRSGGMDEKTIALLVA...GLVITVIVIVGAILFV.PGEYSLKNATGLGLIVTSTGILILLHYYVFSTAIGLTSFVIAILVIQV
Variola_virus AYTSISVVF...STNLGILQVF...GCIIAMIELCGAFLFY.PSMFTLRHIIGLLMMTLPSIFLIITKVFSFWLLCKLSCAVHLIIYYQL
Ectromelia_virus AYTSISVVF...STNLGILQVF...GCVIAMIELCGAFLFY.PSMFTLRHIIGLLMITLPSIFLIITKVFSFWLLCKLSCAVHLIIYYQL
Cowpox_virus TYTSISVVF...STNLGILQVF...GCVIAMIELCGAFLFY.PSMFTLRHIIGLLMMTLPSIFLIITKVFSFWLLCKLSCAVHLIIYYQL
Myxoma_virus CT..ITFKI...NNVSKLIHVY.SIALWMTLIMFVGQYMIGIDTDMLYVKVNGIILIQLSIFSSIFLQRILHK.KLIPSYLLNIVLGLKA
Swinepox_virus II..LIFKH...RNISNFIYIY.AISIWSTLIMLVGQYMISFVTELMYVYIQGIVLIQFSMLSFLFLQKYIYE.QFEYRYMFIIFIIIKV
Sheeppox_virus IATTIKFKL.SFKKDKKIILYLYYIGFVITLIMLLGQYMIGLNTNNIFIRILGTSFIQVSIFISIILQIIFKKKFKNTLSLITVFLLHF
Rabbit_fibroma_virus YT..ITSKT...SNISKLIHVY.SIALWMTIIMFVGQYMIGINTDMIYVKNGIIILIQISIISSVFLQRILHK.TIIPSYLLNIVMGLKV
Goatpox_virus IATTIKFKL.SFKKDKKIILYLYYIGFVITLIMLLGQYMIGLNTNNILIRILGTSFIQLSIFISIILQIIFKKKFKNTLSLITVFLLHF
Monkeypox_virus AYTSISVVF...STNLGILQVF...GCVIAMIELCGAFLFY.PSMFTLRHIIGLLMMTLPSIFLIITKVFSFWLLCKLSCAVHLIIYYQL
Akhmeta_virus TYTSISVVF...STNLGILQVF...GCVIAMIELCGAFLFY.PSMFTLRHIIGLLMMTLPSIFLIITKVFSFWLLCKLSCAVHLIIYYQL
Tanapox_virus LT..LSLKI...NKFKKVINLY.ATLIWVTILCLFIQYILRLNKKYSFIDIYGIMLISVLLFSSIGVNVLTLLKIKKNKYLIVI.IILKT
Cotia_virus FI..VTFRI...NNTKHIINYY.VSIILTTITTISGQYIIL.NEYF...ESYGISLIQLSIFIFVIIQIKLYT.YLNQSYFFLLANIQM
Deerpox_virus IT..ITFKL...NKLKRIIYLY.AVSLWITLILLIGQYMISISSIFTFVKVQGIILIQISILISMFVQIHLYK.YVENSYLIIGNLVLQV
Yokapox_virus IYTSISIRF...TNNVGILQFF...CVIIIMIELSGSLLFY.PSKFNMRHIIGLLMLTIPSIFLIVTKIFSFWVLNKLSFGVHVIIYYQL
Taterapox_virus AYTSISVVF...STNLGILQVF...GCIIAMIELCGAFLFY.PSMFTLRHIIGLLMMTLPSIFLIITKVFSFWLLCKLSCAVHLIIYYQL
Skunkpox_virus AYTSISVIY...STNLGILQVF...GCIIAMIEVSGAFLFY.PSMFTLRHIIGLLMMTLPSIFLIITKVFSFWILSKLSCGVHLIIYYQL
Volepox_virus AYTSISVIY...STNMGILQVF...GCIVVMIEVSGVFLFY.PPMFTLRHIIGLLMMTLPSIFLIITKVFSFWILSKLSCGVHLIIYYQL
Camelpox_virus AYTSTSVVF...STNLGILQVF...GCIIAMIELCGAFLFY.PSMFTLRHIIGLLMMTLPSIFLIITKVFSFWLLCKLSCAVHLIIYYQL
Moosepox_virus IT..ITFKL...NKLKRIIYLY.AVSLWITLIMLVGQYMISISSVFIFVKVQGIILIQISILISMFVQIHLYK.YVENSYLIIGNLVLQV
Yaba_monkey_tumor_virus LT..LSFKI...DRLKRVINLH.AILIWITILGVYIQYMLKLSKGYSFIDVCGIVLITVLLFSSIFVNVLTLLKIKKKCFIVI.IILKT
Horsepox_virus AYTSISVVF...STNLGILQVF...GCIIAMIELCGAFLFY.PSMFTLRHIIGLLMMTLPSIFLIITKVFSFWLLCKLSCAVHLIIYYQL
Alaskapox_virus MYTSISVVF...STNLGILQVF...GCVIAMVELCGAFLFY.PPMFTLRHIIGLLMMTLPSIFLIITKVFSFWLLCKLSCSVHLIIYYQL
consensus>70 ....i..........i...f......i.mi...g.fl......f....i.G..m.........i...v..f......s..v...i.....

230 240 250 260 270 280 290 300
CD47_HUMAN IAYILAVVGLSLCIAACIP.MHGPLLISGLSILALAQLLGLVYMKFVASNQKTIQPPRKAVEEPLNAFKESKGMMNDE.
Variola_virus AGYILTVLGLGLSLKECV...DGTLLLSGLGTIMVSEHFSLLFLVCFPSTQRDYY.........................
Ectromelia_virus AGYILTVLGLGLSLKECV...DGTLLLSGLGTIMVSEHFSLLFLVYFPSTQRDYY.........................
Cowpox_virus AGYILTVLGLGLSLKECV...DGTLLLSGLGTIMVSEHFSLLFLVCFPSTQRDYY.........................
Myxoma_virus VSYTGSTVVIALSFIGCYNKAYGYTYMYKLLFADILELISLIALYTLPLGTQATYKKLYLQSDETFTF..L.........
Swinepox_virus ILFLISSIIIILSFNGCKSIYGYLFMYKLVIDILSLSCLAISVITPLRKNINYKQLHIIQSDSFPFLLKSDYDENCI
Sheeppox_virus ISYIISLIMFTMSLIGCYNEIYGKVIVYKLSLVIIFEFISLLSLFIFSLNTSIKYRKLHIIEDENLLFSI........E
Rabbit_fibroma_virus LSFTGSTIVIALSFVGCYNKAYGYTYVYKLLFADVLELISLITLYVLPFGTQTTYKRLYLQSDETFTF..P.........
Goatpox_virus ISYIISLIMFTMSLIGCYNEIYGKVIVYKLLVIIFEFISLLSLFIFSLNTSIKYRKLHIIEDENLLFSI........E
Monkeypox_virus AGYILTVLGLGLSLKECV...DGTLLLSGLGTIMVSEHFGLLFLVCFPSTQRDYY.........................
Akhmeta_virus AGYILTVLGLGLSLKECV...DGTLLLSGLGTIMVSEHFSLLFLVCFPSTQRDYY.........................
Tanapox_virus VASILFFLVYLLILFYCYKNVFGLFLIYNLLIINIFELSYLVCLLIIPIELRIKYKKLIMIKSNLNL.............
Cotia_virus IFFITSTIIIIISKSECKYIYSCIYSYSIVFILIFSHFYMIILLIAPITYKVKYSNFDIILY..................
Deerpox_virus ISYLISCIILILSFVGCYNQIYIYLFIYKLLFINVLELCNLVLLFITPLGHNIIYKKLHVTTDDIESFSIG.........
Yokapox_virus AGYVLTILGLGLSLKECV..IDGSLLLSGLGIISISEHFSLLLLVCFQSIHREYY.........................
Taterapox_virus AGYILTVLGLGLSLKECV...DGPLLLSGLGTIMVSEHFSLLFLVCFPSTQRDYY.........................
Skunkpox_virus AGYILTVLGLGLSLKECV...DGTLLLSGLGTIMVSEHFSLLFLVCFPSAQRDYY.........................
Volepox_virus AGYILTVLGLGLSLKECV...DGTLLLSGLGTIMVSEHFSLLFLVCFPSTQRDYY.........................
Camelpox_virus AGYILTVLGLGLSLKECV...DGTLLLSGLGTIMVSEHFSLLFLVCFPSTQRDYY.........................
Moosepox_virus ISYLISCIILILSFVGCYNQIYIYLFIYKLLFINVLELCNLVLLFITPLGHNIIYKKLHVTTDDIESFSIG.........
Yaba_monkey_tumor_virus ITSVLFFLTTLVILFYCYKSIFGSLIYKLLIINVFELSHLVILFILPVEFKIEYKKLIILNSNLNL..............
Horsepox_virus AGYILTVLGLGLSLKECV...DGTLLLSGLGTIMVSEHFSLLFLVCFPSTQRDYY.........................
Alaskapox_virus SGYILTVLGLGLNLKECA...DGTLLLSGLGTIMVSEHFSLLFLVCFPSTQRDYY.........................
consensus>70 ..yi.......ls...C.....g.l....l..i.v.e...$..l...p.....y..........................
