# Supplementary material for: Structure of the human marker of self 5-transmembrane receptor CD47
Source: Nat Commun. 2021 Sep 1;12:5218. doi: 10.1038/s41467-021-25475-w (PMC8410850; doi:10.1038/s41467-021-25475-w)
Supplement: Supplementary file 10 — Reporting Summary [file 41467_2021_25475_MOESM10_ESM.pdf]

nature portfolio

Corresponding author(s): Gustavo Fenalti
Last updated by author(s): Aug 3, 2021

Reporting Summary

Nature Portfolio wishes to improve the reproducibility of the work that we publish. This form provides structure for consistency and transparency in reporting. For further information on Nature Portfolio policies, see our Editorial Policies and the Editorial Policy Checklist.

Statistics

For all statistical analyses, confirm that the following items are present in the figure legend, table legend, main text, or Methods section.

n/a	Confirmed	
☐	☒	The exact sample size (*n*) for each experimental group/condition, given as a discrete number and unit of measurement
☐	☒	A statement on whether measurements were taken from distinct samples or whether the same sample was measured repeatedly
☒	☐	The statistical test(s) used AND whether they are one- or two-sided<br>*Only common tests should be described solely by name; describe more complex techniques in the Methods section.*
☒	☐	A description of all covariates tested
☒	☐	A description of any assumptions or corrections, such as tests of normality and adjustment for multiple comparisons
☐	☒	A full description of the statistical parameters including central tendency (e.g. means) or other basic estimates (e.g. regression coefficient) AND variation (e.g. standard deviation) or associated estimates of uncertainty (e.g. confidence intervals)
☒	☐	For null hypothesis testing, the test statistic (e.g. *F*, *t*, *r*) with confidence intervals, effect sizes, degrees of freedom and *P* value noted<br>*Give P values as exact values whenever suitable.*
☒	☐	For Bayesian analysis, information on the choice of priors and Markov chain Monte Carlo settings
☒	☐	For hierarchical and complex designs, identification of the appropriate level for tests and full reporting of outcomes
☒	☐	Estimates of effect sizes (e.g. Cohen's *d*, Pearson's *r*), indicating how they were calculated

Our web collection on statistics for biologists contains articles on many of the points above.

Software and code

Policy information about availability of computer code

Data collection	X-ray diffraction datasets were collected at Diamond Light Source. HDX-MS data was collected on Waters HDX-Synpat G2si HDMS.
Data analysis	HKL2000 (version 7.2.0), Phenix (version 1.14), CCP4i (version 7.0.078), PHASER (version 2.8.3), Refmac (version 5.8.0257), PISA (version 2.1.1), CONTACT (within CCP4i 7.0.078), Coot (0.8.9.2), Pymol (1.8.2.2), UCSF Chimera (version 1.14), Maestro (version 2020.1), PropKa (version 3.1), Desmond (version 2.3), Schrodinger suite (version 2020.1), GROMACS (version 2018.1), Clustal Omega (www.ebi.ac.uk/Tools/msa/clustalo), Tcoffee (www.tcoffee.org), AliView (version 1.26), MEGAX suite (10.1.8), ConSurf (https://consurf.tau.ac.il), Blast (https://blast.ncbi.nlm.nih.gov), Dali server (http://ekhidna2biocenter.helsinki.fi/dali), ProteinLynx Global SERVER (version 3.0.2), DynamX (version 3.0), GraphPad Prism (version 8.0.2), CTFFIND4 (version 4.0), Relion (version 3.0), cryoSPARC (version 2.5), motioncor2 (version 1.1.0)

For manuscripts utilizing custom algorithms or software that are central to the research but not yet described in published literature, software must be made available to editors and reviewers. We strongly encourage code deposition in a community repository (e.g. GitHub). See the Nature Portfolio guidelines for submitting code & software for further information.

Data

Policy information about availability of data

All manuscripts must include a data availability statement. This statement should provide the following information, where applicable:

- Accession codes, unique identifiers, or web links for publicly available datasets
- A description of any restrictions on data availability
- For clinical datasets or third party data, please ensure that the statement adheres to our policy

Atomic coordinates and structure factors of the CD47/BRIL-B6H12 complex have been deposited in the Protein data Bank (www.rcsb.org) with accession code 7MYZ. The source data underlying Fig.4, Supplementary Fig.4, 5 and 6, and Supplementary Data 1, 2, 3 and 4 are provided as a Source Data File. The HDX-MS data

generated in this study have been deposited in the PRoteomics IDEntifications Database (PRIDE; https://www.ebi.ac.uk/pride/archive/projects/PXD026458). For Molecular Dynamics Simulations trajectories and analysis: the data is available upon request. The simulation setup and analysis procedure is detailed in the Methods section and can be easily replicated by anyone with access to MD simulation packages.

Field-specific reporting

Please select the one below that is the best fit for your research. If you are not sure, read the appropriate sections before making your selection.

[x] Life sciences [ ] Behavioural & social sciences [ ] Ecological, evolutionary & environmental sciences

For a reference copy of the document with all sections, see nature.com/documents/nr-reporting-summary-flat.pdf

Life sciences study design

All studies must disclose on these points even when the disclosure is negative.

Sample size	No statistical methods were used to predetermine sample size. The technical replicates for the HDX study were designed based on reasonable yield of recombinant receptor that could be obtained for each construct (WT and mutants). HDX-MS samples for constructs were obtained from independent exchange reactions and analyzed across a wide time range (20sec, 1min, 10min 60 min) in duplicate. The experiments were performed at least two times. Experiments were based on the recommendations provided in the "white paper" for HDX-MS measurements (https://www.nature.com/articles/s41592-019-0459-y).
Data exclusions	No data was excluded from the HDX analysis. Poor quality diffraction data from crystals with unit cell parameters damaged by radiation were excluded.
Replication	All attempts for sample replication in the HDX study were successful. Two independent samples of different protein constructs were tested at different time points in each experiment. The experiments were repeated at least two times freshly purified proteins.
Randomization	The HDX-MS experiments were design to qualitatively differentiate CD47 mutants; randomization is not relevant for this study. Experiments did not allocate experimental groups.
Blinding	Blinding is not relevant to this study as no subjective allocation was involved in any of the structural and HDX experiments.

Reporting for specific materials, systems and methods

We require information from authors about some types of materials, experimental systems and methods used in many studies. Here, indicate whether each material, system or method listed is relevant to your study. If you are not sure if a list item applies to your research, read the appropriate section before selecting a response.

Materials & experimental systems

n/a	Involved in the study	
[ ]	[x]	Antibodies
[ ]	[x]	Eukaryotic cell lines
[x]	[ ]	Palaeontology and archaeology
[x]	[ ]	Animals and other organisms
[x]	[ ]	Human research participants
[x]	[ ]	Clinical data
[x]	[ ]	Dual use research of concern

Methods

n/a	Involved in the study	
[x]	[ ]	ChIP-seq
[x]	[ ]	Flow cytometry
[x]	[ ]	MRI-based neuroimaging

Antibodies

Antibodies used	The Fab from the literature antibody B6H12 was used in this study for structural studies. The antibody is commercially available and its sequence available. CC-90002 is a clinical molecule developed by Bristol Myers Squib.
Validation	The mAb B6H12 and CC-90002 was expressed in ExpiCHO-S cells according to manufacturers instructions. Ab binding to recombinant human full length CD47 confirmed in biophysical experiments and in the crystal structure. The binding of the antibody B6H12 to human CD47 receptor has been described in various studies, recently by Pietsch et al., Blood Cancer Journal 2017 (Anti-Leukemic activity and tolerability of anti CD47 monoclonal antibodies). The development and characterization of CC-90002 is described by Narla et. al., Cancer Immunology, Immunotherapy 2021 (Modulation of CD47-SIRPα innate immune checkpoint axis with Fc-function detuned anti-CD47 therapeutic antibody).

Eukaryotic cell lines

Policy information about cell lines

Cell line source(s)	ExpiCHO-S cells (Thermo Fisher Scientific) was used for over-expression of recombinant CD47
Authentication	Cell line was not authenticated, only used for over-expression of recombinant protein
Mycoplasma contamination	Cell line was not tested for mycoplasma contamination
Commonly misidentified lines (See ICLAC register)	none used
